# A phylogenetic approach to inferring the order in which mutations arise during cancer progression

**DOI:** 10.1371/journal.pcbi.1010560

**Published:** 2022-12-02

**Authors:** Yuan Gao, Jeff Gaither, Julia Chifman, Laura Kubatko

**Affiliations:** 1 Division of Biostatistics, The Ohio State University, Columbus, Ohio, United States of America; 2 Institute for Genomic Medicine, Nationwide Children’s Hospital, Columbus, Ohio, United States of America; 3 Dept of Mathematics and Statistics, American University, Washington D. C., United States of America; 4 Dept of Statistics, The Ohio State University, Columbus, Ohio, United States of America; 5 Dept of Evolution, Ecology, and Organismal Biology, The Ohio State University, Columbus, Ohio, United States of America; Eidgenossische Technische Hochschule Zurich, SWITZERLAND

## Abstract

Although the role of evolutionary process in cancer progression is widely accepted, increasing attention is being given to the evolutionary mechanisms that can lead to differences in clinical outcome. Recent studies suggest that the temporal order in which somatic mutations accumulate during cancer progression is important. Single-cell sequencing (SCS) provides a unique opportunity to examine the effect that the mutation order has on cancer progression and treatment effect. However, the error rates associated with single-cell sequencing are known to be high, which greatly complicates the task. We propose a novel method for inferring the order in which somatic mutations arise within an individual tumor using noisy data from single-cell sequencing. Our method incorporates models at two levels in that the evolutionary process of somatic mutation within the tumor is modeled along with the technical errors that arise from the single-cell sequencing data collection process. Through analyses of simulations across a wide range of realistic scenarios, we show that our method substantially outperforms existing approaches for identifying mutation order. Most importantly, our method provides a unique means to capture and quantify the uncertainty in the inferred mutation order along a given phylogeny. We illustrate our method by analyzing data from colorectal and prostate cancer patients, in which our method strengthens previously reported mutation orders. Our work is an important step towards producing meaningful prediction of mutation order with high accuracy and measuring the uncertainty of predicted mutation order in cancer patients, with the potential to lead to new insights about the evolutionary trajectories of cancer.

## Author summary

Cancer evolves as a consequence of the accumulation of somatic mutations, and diverse clones are formed during this process, resulting in intratumoral heterogeneity (ITH). Similar cancer subtypes often display different landscapes of genetic alterations, and tumors that harbor the same mutations sometimes respond differently to therapy. Recent studies have provided evidence that mutation order is critical in cancer. With application to noisy single-cell sequencing data, we develop a computational framework to reconstruct the mutation order along a phylogeny with high accuracy and to measure the uncertainty in a unique way. We demonstrate that our method exhibits robust performance, both for predicting mutation order and for assessing uncertainty of predicted mutation order. We include applications to prostate cancer and colorectal cancer patients, where we identify mutation orders that have been reported to be important in cancer progression. Knowledge of the tumor evolutionary history, especially the mutation order, would greatly improve our understanding of a tumor’s ITH and aid in treatment decisions. Our work is a powerful computational tool that can be applied to address research questions in the field of cancer and has important translational applications for improving cancer diagnosis and personalized therapy.

This is a *PLOS Computational Biology* Methods paper.

## 1 Introduction

**Fig 1:**
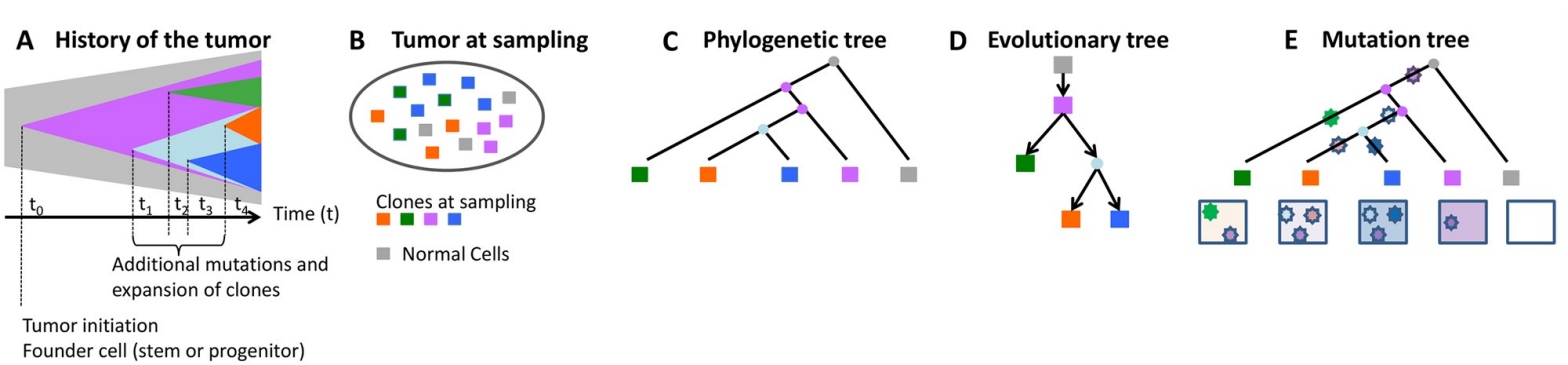
Pictorial representation of tumor evolution. (A—B) A pictorial representation of the evolution of a tumor from the initiating mutation to the heterogeneous tissue at the time of sampling, which consists of four different clones and normal tissue. (C) A phylogenetic tree with single cells as the tips. (D) A clonal lineage tree inferred from sampled cells where each node represents a subclone (cluster of cells). (E) A mutation tree inferred from sampled cells where each star represents the occurrence of one mutation. The box underneath each tip shows which mutations are present in the cell represented by the tip.

Cancer progression is a dynamic evolutionary process that occurs among the individual cells within each patient’s tumor. Cancer typically develops from genetic alterations to a single cell in normal tissue that endow a growth advantage over the surrounding cells, allowing that cell to replicate and to expand, resulting in the formation of a clonal population. Cells within this clonal population may then undergo their own somatic mutations, followed by replication and formation of subclones. During this complex process, several competitive and genetically diverse subpopulations may be formed, resulting in intratumoral heterogeneity, as depicted in Fig 1A and 1B [1–5]. An important consequence of this process of tumor evolution is that the order in which mutations arise within the tumor may have an impact on cancer progression (see, e.g., [5–7]), and previous work has been devoted to developing models for the accumulation of mutations within tumors, comparing model predictions with empirical evidence, and developing methods for inferring the order of mutations from data. Examples of such studies can be found in [4, 8–19] and vary in terms of the type of data and methods used (several of these are discussed below). Empirical evidence also points to the importance of mutation order in cancer progression. For example, Ortmann et al. [20] demonstrate that the type of malignancy and the response to treatment of myeloproliferative neoplasm patients are affected by the order in which somatic mutations arose within the patients’ tumors. Thus, while the importance of mutation order is strongly justified by both theory and empirical studies, study of mutation order is complicated because this cannot be observed directly as cancer initiation time is unknown in most patients and genomic data are most often collected at one snapshot in time. Consequently, use of computational methods that infer the order of mutations from DNA sequence data is the approach of choice for studying this phenomenon.

Most studies on cancer phylogenetics utilize bulk high-throughput sequencing data, but signals from bulk sequencing only reflect the overall characteristics of a population of sequenced cells, rather than the characteristics of individual cells. For example, some methods infer mutation order by comparing occurrence frequencies of mutations across bulk sequencing data from different tumor samples and patients [8, 10]. However, variation in the mutations among different cells in a tumor is difficult to evaluate from bulk sequencing data alone. Single-cell sequencing (SCS) is promising because it enables sequencing of individual cells, thus providing high-resolution data that can be used to infer the mutational history of cancer [21]. However, the high error probabilities associated with SCS data complicate the development of methods for inference of the mutational history. The whole-genome amplification (WGA) process used to produce SCS data results in a variety of errors, including allelic dropout (ADO) errors, false positives (FPs), non-uniform coverage distribution, and low coverage regions. ADO contributes a considerable number of false negatives (FNs) to point mutations [21].

Several recent studies have proposed various mathematical methods to infer mutation order (Fig 1C–1E) from data arising from somatic mutations (i.e., [16, 18, 22–25]). Among these, we focus specifically on the methods of Jahn et al. [22], Zafar et al. [16], and El-Kebir [18], called SCITE, SiFit and SPhyR, respectively, as these methods use single-cell data for inference of the order in which mutations arise along a phylogeny as part of their estimation procedure. SiFit estimates the mutation order by estimating the most likely mutation states of the tips and internal nodes of the phylogeny using a dynamic programming algorithm. SCITE estimates the mutational history with error models only but ignores the evolutionary process of somatic mutation. SPhyR estimates the mutational history by employing the k-Dollo evolutionary model. However, none of the above methods provide probabilistic information for the inferred mutation order along a given phylogeny, and thus uncertainty in the inferred mutational signatures along the phylogeny cannot be readily assessed using existing methods. In addition to methods using SCS data alone, methods that estimate mutational history by integrating both SCS data and bulk sequencing data have been developed (e.g., [19]), but such methods generally require sequencing of both single cells and bulk samples.

Here, we propose a novel method for mapping mutations onto a phylogenetic tree to allow inference of the order in which mutations arise within an individual tumor given SCS data from the tumor at a single time point. Our approach utilizes models for both the mutational process within the tumor and the errors that arise during SCS data collection in a Bayesian framework, thus allowing us to predict the mutation order as well as quantify the uncertainty in the inferred mutation order along the fixed tumor phylogeny. Our approach thus represents a conceptually distinct and practically important extension of earlier methods. We demonstrate the performance of our method by comparing it to existing methods. Finally, we apply our method to real data to estimate the mutation order for prostate and colorectal cancer patients. Our real data analyses confirm mutation orders that have been demonstrated to be critical in cancer initiation and progression.

## 2 Results

### 2.1 Overview of mutation order inference from single-cell sequencing data

A phylogenetic tree T displays the evolutionary relationships among a sample of *J* cells within a tumor. To infer the locations (branches) on which a set of somatic mutations are acquired within a given phylogenetic tree, we need to model the evolutionary process of these somatic mutations and quantify the technical errors that arise from the SCS data collection process. In this section, we describe the models that we apply for this problem. Indeed, one potential outcome of our approach to mapping mutations onto a fixed phylogeny may be the identification of mutations whose ordering across patients suggests an important role in the development, progression, or treatment of cancer.

#### 2.1.1 Notation and terminology

Consider somatic mutations of interest at *I* loci across the genome for a sample of *J* single cells. The *J* single cells are sampled from the tumor, and we assume that their phylogenetic relationships are given. In practice, we will often first estimate the phylogeny from the data. The mutation data can be either binary or ternary. For binary data, 0 denotes the absence of mutation and 1 means that mutation is present, while for ternary data, 0, 1 and 2 represent the homozygous reference (normal), heterozygous (mutation present) and homozygous non-reference (mutation present) genotypes, respectively.

The *I* somatic mutations evolve along the tumor phylogenetic tree T. Each tip in T represents one single cell *C*_*j*_, where *j* = 1, …, *J*. Let *C* = {*C*_1_, …, *C*_*J*_} be the set of the *J* single cells under comparison. T=(T,t) includes two parts: the tree topology *T* and a vector of branch lengths **t**. The tree topology *T* = (*V*, *E*) is a connected graph without cycles and is composed of nodes and branches, where *V* is the set of nodes and *E* is the set of branches. The root *r* of T represents the common ancestor (a normal cell without somatic mutations) for all the single cells under comparison. In the context of this paper, we will focus on rooted bifurcating trees. There are 2*J* − 2 branches in a rooted bifurcating tree with *J* tips, i.e., *E* = {*e*_1_, *e*_2_, …, *e*_2*J*−2_}. Let *v* and *w* be two nodes in the node set *V* that are connected by the branch *x* in the branch set *E* (i.e., *x* = {*v*, *w*}: *v* is the immediate ancestor node of *w*, and *x* connects *v* and *w*). Then the set *U*^*x*^(*w*), which includes the node *w* and all nodes descended from *w* in T, is called the *clade induced by w*. The branch *x* connects the ancestor node *v* and the clade induced by *w*, and we define branch *x* as the *ancestor branch of clade U^x^(w)*. *E*^*x*^(*w*) is a subset of *E* that includes branches connecting nodes in *U*^*x*^(*w*), and *C*^*x*^(*w*) are the tips in *U*^*x*^(*w*).

**Fig 2:**
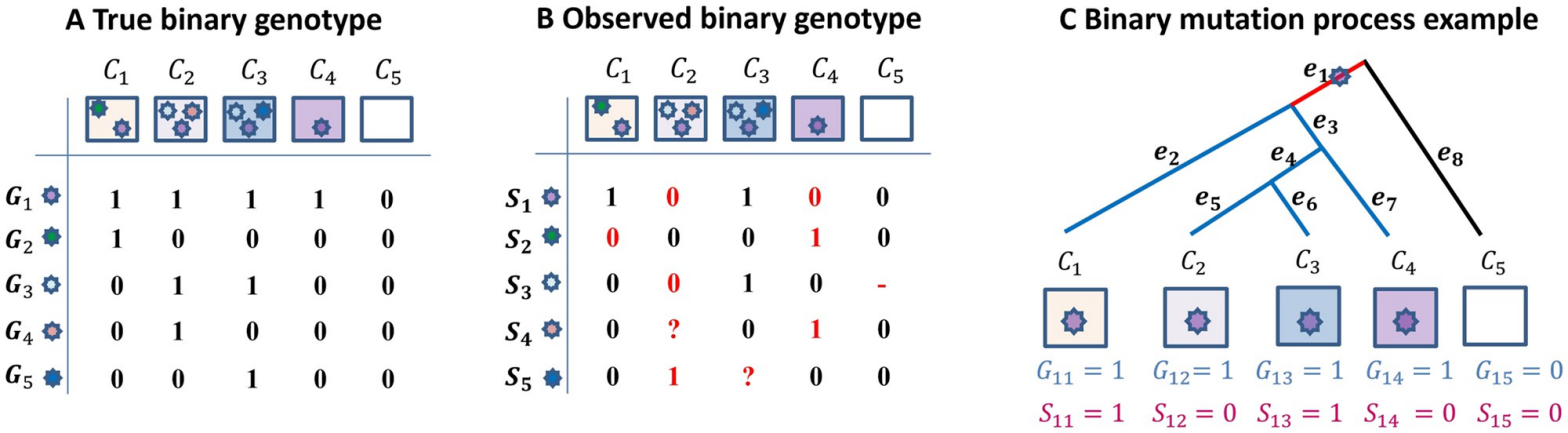
True binary data, observed binary data and binary mutation process example. (A) True binary mutation matrix of the sequenced tumor cells in the mutation tree in Fig 1E. Each row represents true genotypes for one genomic site in all cells and each column represents the true genotypes of multiple genomic sites for one single cell. (B) Observed mutation matrix with missing and ambiguous values (red), as well as mutation states that are misrecorded with respect to the true mutation matrix (red numbers; these are either false positives or false negatives). The red dash indicates a missing value since the sequencing process does not return signal at this site of this cell, and the red question mark represents an ambiguous value. Each row represents observed states for one genomic site in all cells and each column represents the observed states of multiple genomic sites for one single cell. (C) Binary mutation process example. A mutation is acquired on branch *e*_1_ (highlighted in red). The cell descending from branch *e*_8_ (highlighted in black) does not carry the mutation, while the cells descending from the blue branches carry the mutation.

Let *G*_*ij*_ denote the true genotype for the *i*^*th*^ genomic site of cell *C*_*j*_. The *i*^*th*^ genomic site will then have a vector **G**_*i*_ ∈ {0, 1}^*J*^ (for binary data) or {0, 1, 2}^*J*^ (for ternary data) representing its true genotype for all the *J* cells represented by the tips in the tree, where *i* = 1, …, *I*. Let *S*_*ij*_ denote the observed data for the *i*^*th*^ genomic site of cell *C*_*j*_. Due to the technical errors associated with SCS data, the observed data *S*_*ij*_ does not always equal the true genotype *G*_*ij*_. For both binary and ternary data, the observed state *S*_*ij*_ might be flipped with respect to the true genotype *G*_*ij*_ due to FP or FN. Missing states (“-”) or ambiguous states (“?”) may be present for some genomic sites as well. Fig 2 shows an example of true and observed binary genotype data for the mutations in Fig 1. In Fig 2, the observed state is highlighted in red if it is not consistent with the true genotype. The red numbers are those mutations with flipped observed mutation states relative to the true mutation states. The red dash (“-”) indicates a missing value, and the red question mark (“?”) represents an ambiguous value.

Mathematically, we represent the observed mutation states of the *J* single cells at *I* different genomic sites by a *I* × *J* mutation matrix **S** for convenience,

$$\begin{array}{c} S=(\begin{array}{c} S_{1}\\ S_{2}\\ \cdots \\ S_{I} \end{array})=(\begin{array}{ccc} S_{11} & \cdots & S_{1J}\\ S_{21} & \cdots & S_{2J}\\ \cdots & \cdots & \cdots \\ S_{I1} & \cdots & S_{IJ} \end{array}). \end{array}$$ (1)

Each entry (*i*, *j*) denotes the state observed for mutation *i* in cell *C*_*j*_, so **S**_*i*_ gives the observed data for genomic site *i* as a vector with *J* values corresponding to the *J* single cells. Column *j* represents the somatic mutations for cell *C*_*j*_. In phylogeny T, let B be the vector of locations (branches) on which the *I* mutations occur, i.e., $B=\{B_{1},\ldots ,B_{I}\}$, where *B*_*i*_ is the location (branch) on which mutation *i* is acquired. Note that *B*_*i*_ takes values in {*e*_1_, *e*_2_, …, *e*_2*J*−2_}.

#### 2.1.2 Somatic mutation process

To model the somatic mutation process, we consider continuous-time Markov processes, which we specify by assigning a rate to each possible transition between states [26]. The mutation processes along each branch of T are assumed to be independent of each other. We consider point mutations. Once a mutation *i* is acquired on a branch *x* ∈ *E*, all the branches in the set *E*^*x*^(*w*) will harbor mutation *i* but those branches in the set *E*∖(*x* ∪ *E*^*x*^(*w*)) will not carry this mutation (see Methods) [27]. As an example, Fig 2C depicts the observed and true binary genotype for mutation *i* = 1 shown in Fig 2A and 2B. The set of branches is *E* = {*e*_1_, …, *e*_8_} and the corresponding set of branch lengths is **t** = {*t*_1_, …, *t*_8_}. If mutation *i* is acquired on branch *e*_1_, the cell descending along branch *e*_8_ will not carry the mutation, while those descending from the blue branches would carry this mutation. The assumptions that all descendent branches retain a mutation and that the mutation arises only once correspond to an infinite sites model. Although it is common to assume an infinite sites model for studying tumor progression, the fit of the infinite sites model to the mutation process underlying cancer has been discussed (see, e.g., [27]). To evaluate the sensitivity of our method to this assumption, we include several simulations under finite sites models below.

#### 2.1.3 Observation of SCS errors

Real data in the observed mutation matrix are subject to errors. To account for FPs and FNs in the SCS data, our method applies the error model from Zafar et al. (2017) [16]. Following their notation, we let *α*_*ij*_ be the probability of a false positive error and *β*_*ij*_ be the probability of a false negative error for genomic site *i* of cell *C*_*j*_. For binary data, if the true genotype is 0, we may observe a 1, which is an FP. If the true genotype is 1, we may observe a 0, which is an FN. The conditional probabilities of the observed data given the true genotype at genomic site *i* of cell *C*_*j*_ can be stored in an error probability matrix **N**^*ij*^ (see Methods). It has been pointed out (see, e.g., [24]) that parametrizing error using FP and FN rates does not capture some plausible events, such as an amplification/sequencing error leading to the identification of a heterozygous mutant as a homozygote. Models parameterized by the ADO rate and the amplification/sequencing error rate, rather than by the FP and FN rates, have been proposed [24]. We note that such error models could easily be incorporated into our method.

#### 2.1.4 Inferring the locations and ordering of mutations in T

Once the observed matrix **S** = [**S**_1_…**S**_*I*_]^*T*^ of the *I* mutations has been collected, the next step is to infer the branch on which mutation *i* takes place, conditioning on **S**. Given the observed data matrix **S**, the tumor phylogenetic tree T, the error probability matrix **N** = {**N**^*ij*^|1 ≤ *i* ≤ *I*, 1 ≤ *j* ≤ *J*}, and the mutation process $Q_{\lambda }$, we can assign a posterior probability distribution $P(B_{i}|S,T,N,Q_{\lambda })$ to the location of mutation *i* using Bayes’ theorem, i.e, we can compute the probability that mutation *i* occurs on each of the 2*J* − 2 branches. To obtain a point estimate of the branch on which mutation *i* occurs, we pick the branch that maximizes this posterior probability, i.e., the maximum a posteriori (MAP) estimate.

We now consider the posterior probability distribution of the locations for the *I* mutations in the sample of *J* single cells over branches of T, which is a distribution on a set of cardinality (2*J* − 2)^*I*^. Under the independence assumption of the somatic mutations during the evolutionary process across sites, the posterior distribution for B is given by $P\left(B|S,T,N,Q_{\lambda }\right)=\prod_{i=1}^{I}P\left(B_{i}|S,T,N,Q_{\lambda }\right)$. From this distribution, we can extract information on the ordering of mutations by picking the MAP estimate, and measure the uncertainty of the inferred mutation order (Methods).

### 2.2 Simulation study

To evaluate the ability of our method, which we call MO (Mutation Order), to correctly identify the locations and the order of a set of mutations under different conditions, we conducted a series of simulation studies with data simulated under different assumptions. The goal was to assess the effect of data quality (complete or incomplete, high or low error probabilities), number of cells, branch lengths, number of mutations and type of genotype data on the performance of our method. We considered a total of 9 scenarios, with 100 replicates for each setting within each scenario. Scenarios 1—4 considered data simulated under various models implemented in the CellCoal software [28]. Scenarios 5 and 6 involved data generated under our model, with mutations placed on branches with varying probabilities. Scenarios 7 and 8 considered data simulated under the finite sites assumption (all other simulation settings use the infinite sites assumption). Finally, to assess the scalability of the method, we simulated scenario 9 under models implemented in CellCoal with 500 or 1000 cells in each replicate. Section A of the S1 Text provides information about computational requirements.

#### 2.2.1 Simulation method 1

The first method of simulation is conducted with CellCoal [28]. In each tree, mutations occur under the infinite sites diploid model. The simulation consists of the following steps:

1. Simulate 100 random tumor trees with *J* cells in each tree. Each tree is generated under the standard neutral coalescent, going backward in time.
2. Repeat steps (2.a)-(2.c) for each of the 100 simulated trees from step (1).
  - Simulate 1000 genomic sites, obtained from a population with an effective size of 10000, a growth rate of 0.001, mutation rate 10^−7^, and *I* mutations with genotyping errors occurring with probabilities *α* and *β* taking place along the sample genealogy according to an infinite sites diploid model.
  - Record the true and observed genotype data.
  - Randomly select tips in the tree with a prespecified missing data percentage and delete the observed data for these tips. Record the incomplete observed data.
3. Repeat step (2) with different choices of the number of mutations (*I*), error probabilities (*α* and *β*) and missing data percentages.

Given the above simulation procedure, there are several options at each step. In step (1), we need to specify the number of tips. We considered 10 tips (labeled scenario 1) or 50 tips (labeled scenario 2). We repeat step (3) 108 times, each with a unique choice of number of observed mutations (*I* ∈ {20, 40, 80}), genotyping error probability (here we consider values of *α* = {0, 0.05, 0.1} and *β* = {0, 0.1, 0.25, 0.5}) and missing data percentage (complete, 10% missing and 20% missing).

To evaluate MO’s performance when some mutations are lost, we subsequently introduce losses in scenarios 1 and 2 with loss rate *r* and maximum number *k* losses per mutation. For a mutation that has been lost at most *k* − 1 times, we randomly select a branch and introduce a loss for that mutation with probability *r* on the selected branch. Cells descending from the selected branch will lose the mutation with probability *r*. We use a varying number *k* ∈ {1, 2} maximum losses per mutation and loss probability *r* ∈ {0.1, 0.2}. We label the settings in which mutations can be lost as scenario 3 (10 tips) and scenario 4 (50 tips).

Finally, to assess the scalability of MO, we simulated scenario 9 with a large number of cells (500 and 1000 cells, with 1000 and 10,000 sites) as well as with a small number of cells (10 and 50 cells, with 20, 40 and 80 sites). Settings were similar to those specified for scenarios 1 and 2. For simplicity, we explored only one error rate setting (*α* = *β* = 0.1) for trees with a large number of cells. We explored three error rate settings (*α* = *β* = {0, 0.05, 0.1}) for trees with a small number of cells. We only analyzed the first 20 replicates in this scenario due to the high computational cost and prohibitive running times for competing tools.

#### 2.2.2 Simulation method 2

The second method of simulation assesses MO’s performance for scenarios in which mutations evolve with the rates λ_1_ and λ_2_. The simulation consists of the following steps:

1. Simulate 100 random bifurcating tumor trees with *J* cells in each tree. Each tree is generated by the recursive random splitting algorithm of the R package *ape* [29].
2. Repeat steps (2.a)-(2.d) on each of the 100 simulated trees from step (1).
  - For each mutation, simulate its location and generating mechanism on the tree based on the mutation process in Section 4. Record the perfect true ternary genotype data for the *J* cells of each mutation.
  - Convert the simulated true ternary genotype data (1 and 2) from step (1.a) into binary genotype data (1). Record the complete true genotype data.
  - Add noise to the true genotype data with error probabilities *α* and *β*, and record the observed data for each mutation. The noise is added to each cell independently.
  - Randomly select tips in the tumor tree with a prespecified missing data percentage and delete the observed data for these tips. Record the incomplete observed ternary and binary data.
3. Repeat step (2) with different choices of number of mutations (*I*), error probabilities (*α* and *β*) and missing data percentages.

Given the above simulation procedure, there are several options at each step. In step (1), we need to specify the number of tips and the branch length distribution in each tree. We let each tree have 10 tips (labeled scenario 5) or 50 tips (labeled scenario 6) and consider branch lengths that follow the exponential distribution with mean 0.2. Step (1) returns 100 simulated trees in each of scenarios 5 to 6.

In step (2.a), we specify the parameters, and consider λ_1_ = 10^−7^ and λ_2_ = 10^−2^ from Iwasa et al. [26]. For each mutation, we select its location on the tree and genotype based on the mutation process in Section 4. In step (2.c), we add noise to each tip with error probabilities *α* and *β* independently based on Expression (15). In step (2.d), we choose 10% or 20% of the tips and delete their data. These loci have missing values at the selected tips.

We repeat step (3) 108 times, each with a unique choice of the number of mutations (*I* ∈ {20, 40, 80}), genotyping error probability (*α* = {0, 0.05, 0.1} and *β* = {0, 0.1, 0.25, 0.5}) and missing data percentage.

#### 2.2.3 Simulation method 3

The third method of simulation assesses MO’s performance under the finite sites assumption. The simulation consists of the following steps:

1. Repeat steps (1.a)-(1.b) on each of the 100 simulated trees from Section 2.2.1.
  - Simulate mutations under the finite sites assumption for binary genotypes using the function “sim.history” in the *phytools* package in R [30].
  - Add noise to the true genotype data with error probabilities *α* and *β*, and record the observed data for each mutation. The noise is added to each cell independently.
2. Repeat step (1) with different choices for the transition rates and error probabilities (*α* and *β*).

In step (1), we use the labels scenario 7 (10 tips) and scenario 8 (50 tips). In step (1.a), we set the rate of mutating from 0 to 1 to be 100 and consider three different rates of mutating from 1 to 0 (1, 10 or 100). In step (1.b), we add noise to each tip with error probabilities *α* and *β* independently.

#### 2.2.4 Accuracy of MAP estimates

We assessed the accuracy of the MAP estimates in MO across the 100 trees within each simulation setting in several ways, including whether the mutation was inferred to occur on the correct branch (“location accuracy”), whether any pair of mutations were inferred to occur in the correct order (“order accuracy”), and whether a pair of mutations that occurred on adjacent branches were inferred to occur in the correct order (“adjacent order accuracy”). In evaluating both the order accuracy and adjacent order accuracy, if two sequential mutations were inferred to occur on the same branch, then it was counted as ordering the mutations incorrectly. In addition, pairs of mutations that occurred on the same branch were counted as successfully ordered for both the order accuracy and the adjacent order accuracy if they were inferred to occur on the same branch. The details of how the MAP estimates were assessed, including an example of the order accuracy and adjacent order accuracy, are given in the Methods section.

Tables A and B in the S1 Text show the location accuracy for scenarios 1 and 2, respectively, with each cell entry corresponding to a unique setting of error probabilities (*α* and *β*), type of genotype (binary and ternary) and missing data percentage (complete and incomplete data). In most cases, the location accuracy of MO is high except when the error probabilities are high. With the same type of genotype and same error probability setting, the accuracy decreases as the percentage of missing values increases. When *α* (or *β*) is fixed, accuracy decreases as *β* (or *α*) increases.

The results for order accuracy (Tables C and D in the S1 Text) and adjacent order accuracy (Tables E and F in the S1 Text) for scenarios 1 and 2 are similar. In addition to the same overall trend due to number of cells, data type, percentage of missing data and error probabilities, the order accuracy rates are higher than the corresponding adjacent order accuracy rates. The results for location accuracy, order accuracy and adjacent order accuracy of MO in scenarios 3 to 6 have similar patterns to those observed for scenarios 1 to 2.

#### 2.2.5 Credible set accuracy

The credible set accuracy of the inferred mutation branch was assessed as well. If the true mutation branch was within the credible set, we counted this as correct; otherwise, it was incorrect. We used 95% credible set for computation (Tables G and H in the S1 Text for scenarios 1 and 2, respectively). The credible set accuracy has the same overall trend as the accuracy of MAP estimates due to the number of cells, type of genotype, missing data percentage and error probabilities, though the accuracy is higher than that of the corresponding MAP estimates, especially for settings with large error probabilities and high missing data percentages. The overall trend for scenarios 3 to 6 is similar to scenarios 1 to 2.

#### 2.2.6 Comparison with competing approaches

To further assess the performance of MO, we compared its performance with the methods SCITE [22], SiFit [16] and SPhyR [18] for the simulation data in scenarios 1 to 9. SCITE can estimate the order of mutations for either binary or ternary genotype data. We used the maximum likelihood mutation order inferred by SCITE with 1,000,000 iterations given the true error probabilities. SiFit can only use binary genotype data when inferring mutation order. We estimated the most likely mutational profiles for the tips and the internal nodes by SiFit given the true phylogenetic tree, error probabilities and mutation rates. We then extracted the mutation order information from the output. SPhyR can estimate the mutational history for binrary genotype data. We estimate the order of mutations by SPhyR given the true error probabilities. We estimated the mutation order with MO conditional on the true tree and estimated tree, while using Monte Carlo integration to integrate over the distributions of the transition rates and error rates. The four methods were compared with respect to the order accuracy and adjacent order accuracy for the above simulation settings.

**Fig 3:**
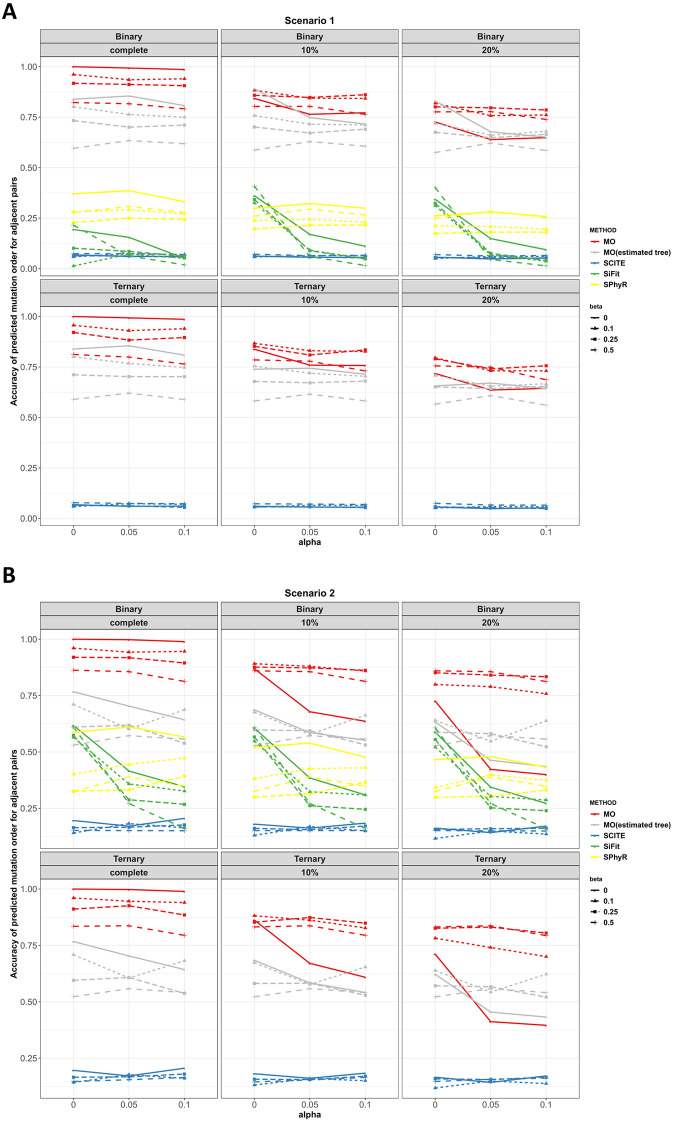
Adjacent order accuracy in scenarios 1 and 2 for MO, SCITE, SiFit and SPhyR when there are 20 mutations. Each panel includes the results from the specific type of genotype and missing data percentage. In each panel, red, gray, blue, green and yellow colors correspond to MO with the true tree, MO with the estimated tree, SCITE, SiFit and SPhyR, respectively. Each plotting symbol on the line represents a different *β*. The x-axis is the probability of a false positive error, *α*, and the y-axis is order accuracy.

**Fig 4:**
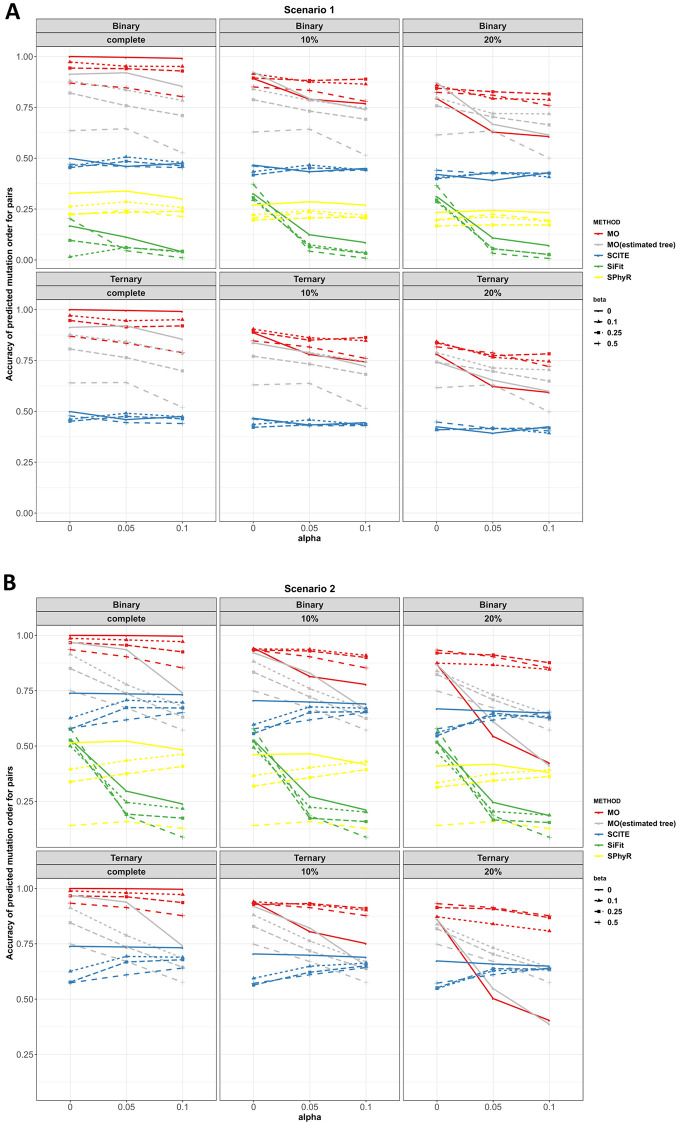
Order accuracy in scenarios 1 and 2 for MO, SCITE, SiFit and SPhyR when there are 20 mutations. Each panel includes the results from the specific type of genotype and missing data percentage. In each panel, red, gray, blue, green and yellow colors correspond to MO with the true tree, MO with the estimated tree, SCITE, SiFit and SPhyR, respectively. Each plotting symbol on the line represents a different *β*. The x-axis is the probability of a false positive error and the y-axis is order accuracy.

**Scenarios 1 to 4.** Figs 3 and 4 plot the adjacent order accuracy and the order accuracy for the four methods in scenarios 1 to 2, respectively. In scenarios 1 to 2, order accuracy and adjacent order accuracy decrease as data quality becomes worse for all four methods. MO is superior to SCITE and SPhyR in most settings in terms of adjacent order accuracy and order accuracy. In all the settings, SiFit has the worst performance with respect to order accuracy. However, SiFit has higher adjacent order accuracy than SCITE. In all settings, SiFit has worse performance since only a subset of the input mutations are inferred to occur on the tree (i.e., some observed mutations are inferred by SiFit to be due only to error, and thus SiFit does not map these mutations onto the phylogeny). Although the output partial mutation order from SiFit is mostly correct, the accuracy is low due to the small number of inferred mutation orders. MO thus dominates SiFit in scenarios 1 and 2. The order accuracy and adjacent order accuracy of SCITE decrease as the number of mutations increases when error probabilities are large.

**Fig 5:**
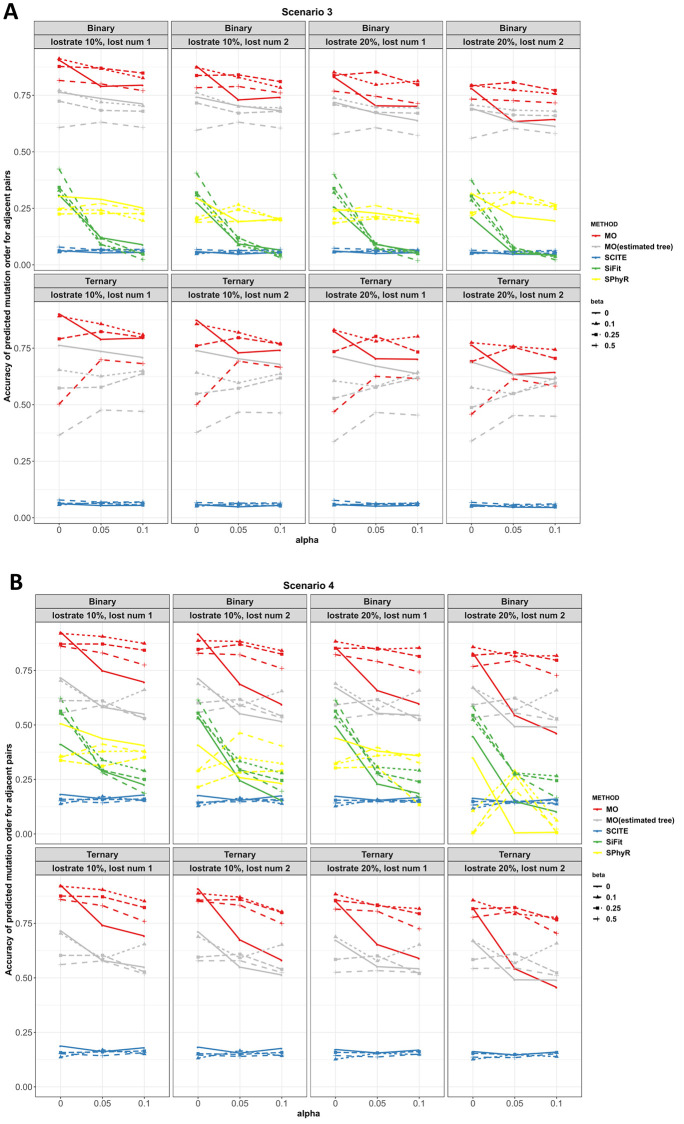
Adjacent order accuracy in scenarios 3 and 4 for MO, SCITE, SiFit and SPhyR when there are 20 mutations. Each panel includes the results from the specific type of genotype and lost mutations. In each panel, red, gray, blue, green and yellow colors correspond to MO with the true tree, MO with the estimated tree, SCITE, SiFit and SPhyR, respectively. Each plotting symbol on the line represents a different *β*. The x-axis is the probability of an error, *α*, and the y-axis is adjacent order accuracy.

**Fig 6:**
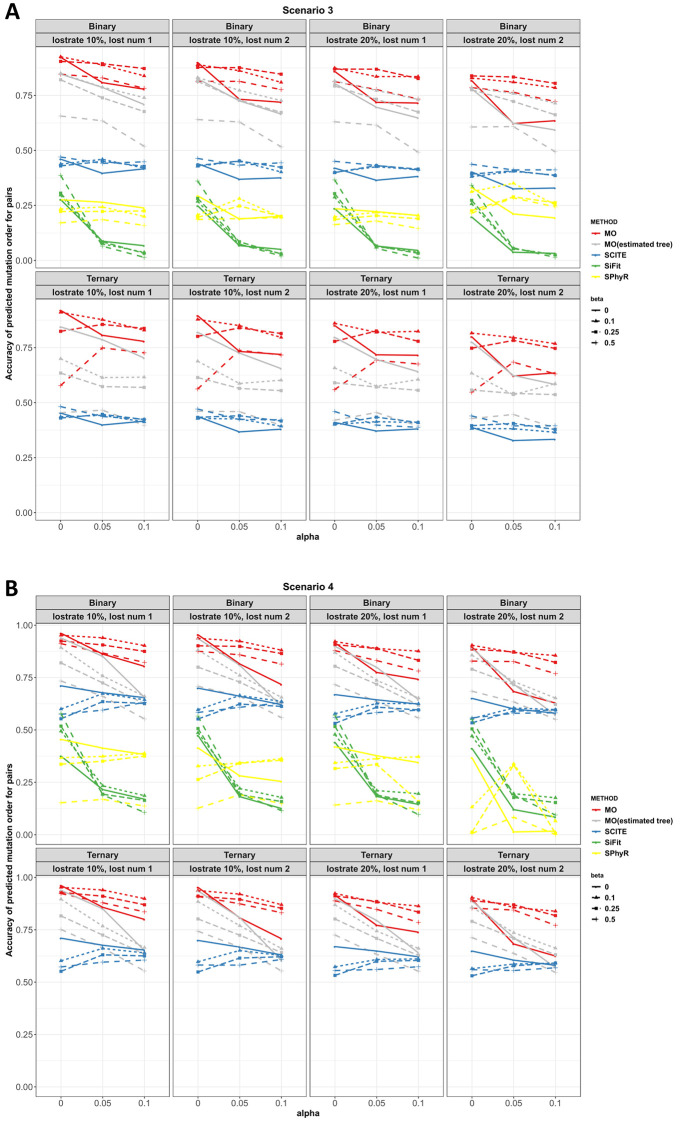
Order accuracy in scenarios 3 and 4 for MO, SCITE, SiFit and SPhyR when there are 20 mutations. Each panel includes the results from the specific type of genotype and lost mutations. In each panel, red, gray, blue, green and yellow colors correspond to MO with the true tree, MO with the estimated tree, SCITE, SiFit and SPhyR, respectively. Each plotting symbol on the line represents a different *β*. The x-axis is the probability of an error, *α*, and the y-axis is order accuracy.

Figs 5 and 6 plot the adjacent order accuracy and the order accuracy for scenarios 3 and 4, respectively. In scenarios 3 and 4, mutations arise under the infinite sites diploid model, as was the case for scenarios 1 and 2, but now a small proportion of the mutations are lost. Compared to the complete settings in scenarios 1 and 2, the performance of all the four methods is worse. However, the performance of the four methods is comparable to settings with missing values in scenarios 1 and 2. MO outperforms SCITE, SiFit and SPhyR in all settings in terms of adjacent order accuracy and order accuracy.

**Scenarios 5 and 6.** In scenarios 5 and 6, mutations are simulated under the mutation process defined in Section 4.2. In Figs F through K in the S1 Text, we observe that MO has higher accuracy than SCITE, SiFit and SPhyR in almost all settings in terms of both order accuracy and adjacent order accuracy.

**Scenarios 7 and 8.** In scenarios 7 and 8, mutations are simulated under the finite sites assumption. Because it is unclear how mutation order should be defined when mutations can arise multiple times along a phylogeny, we instead plot the location accuracy of MO and SiFit in Fig L in the S1 Text. When there are only 10 tips in the tree, most simulated mutations occur only once along the tree and do not mutate back to normal state and MO has higher accuracy than SiFit. However, when there are 50 tips, most mutations are back mutations that mutate back to normal state along the tree. SiFit performs better than MO when the rate of mutating from 1 to 0 is low. When the rate of mutating from 1 to 0 is high, neither MO nor SiFit identify the correct mutation location. MO is limited by its assumption that all mutations occur only once on the tree. Although SiFit can infer parallel/back mutations, it is not able to identify all the locations on which the mutations occur for the simulated data.

**Scenario 9.** We assessed accuracy on large scDNA-seq datasets with up to 1,000 cells and 10,000 sites for MO as well as the other methods considered, with the exception of SCITE when the data contained 10,000 sites as the program was still running after several days. The results are shown in Fig M in the S1 Text. MO continues to show higher accuracy than all of the other methods considered for these larger datasets.

### 2.3 Empirical examples

We apply MO to two experimental single-cell DNA sequencing datasets, one for prostate cancer [31] and one for metastatic colorectal cancer patients [32]. For the prostate cancer dataset, we retrieve publicly available data from the single-cell study of Su et al. (2018) [31], which includes 10 single-cell genomes for each patient. For the colorectal cancer dataset, we use the somatic single nucleotide variants (SNVs) after variant calling provided in the original study (16 SNVs for patient CRC1 and 36 SNVs for patient CRC2) of [32].

#### 2.3.1 Prostate cancer data

**Data analysis.** To infer tumor evolutionary trees for patients 1 and 2 (labeled P1 and P2), we used the SVDQuartets method [33] as implemented in PAUP* [34] using the aligned DNA sequences for all somatic mutations as input with the expected rank of the flattening matrix set to 4. We specified the normal cell sample as the outgroup. We used the maximum likelihood method to estimate the branch lengths.

We selected common tumor suppressor genes and oncogenes for both P1 and P2 identified by Su et al. [31]. In addition to these common cancer-associated genes across different cancers, we mapped mutations in prostate cancer-specific genes (genes that are more commonly mutated in prostate cancer patients) suggested by Barbieri et al. [35] and Tate et al. [36]. For both binary and ternary genotype data for these genes, we used MO to compute the posterior probability of mutation on each branch of the tumor phylogeny for each of the two patients.

Su et al. (2018) estimated the error probabilities to be (*α*, *β*) = (0.29, 0.02) for P1, and (*α*, *β*) = (0.31, 0.02) for P2. To examine the effect of informativeness of the prior distribution on the resulting inference, we considered two prior distributions for each parameter with mean equal to the estimated error probability from the empirical data and with either a large or a small variance as described in Section 4.8. For P1, we considered *α*|**S**_*i*_ ∼ *Beta*(0.29, 0.71) (larger variance) and *α*|**S**_*i*_ ∼ *Beta*(2.9, 7.1) (smaller variance). For P2, we considered *α*|**S**_*i*_ ∼ *Beta*(0.31, 0.69) (larger variance) and *α*|**S**_*i*_ ∼ *Beta*(3.1, 6.9) (smaller variance). For *β* for both P1 and P2, we considered *β*|**S**_*i*_ ∼ *Beta*(0.02, 0.98) (larger variance) and *β*|**S**_*i*_ ∼ *Beta*(0.2, 9.8) (smaller variance).

According to Iwasa et al. [26], the mutation rates for the first and second mutation were estimated to be λ_1_ = 10^−7^ and λ_2_ = 10^−2^, respectively. We used these values to specify the prior distributions for the transition rates. Similar to the sequencing error probabilities, we set two prior distributions for each transition rate with equal means but different variances. The distribution of the transition rate λ_1_ (0 → 1 for ternary genotype) was set as λ_1_|**S**_*i*_ ∼ *Gamma*(2, 5.0 × 10^−8^) (larger variance) and λ_1_|**S**_*i*_ ∼ *Gamma*(5, 2.0 × 10^−8^) (smaller variance). The distribution of the transition rate λ_2_ (1 → 2 for ternary genotype) was set as λ_2_|**S**_*i*_ ∼ *Gamma*(2, 5.0 × 10^−3^) (larger variance) and λ_2_|**S**_*i*_ ∼ *Gamma*(5, 2.0 × 10^−3^) (smaller variance). The estimated probabilities of mutation did not vary substantially when the prior distributions with larger or smaller variance were used for any of these parameters. The heatmaps of estimated probabilities with different prior distributions (larger or smaller variance) are in Figs R through Y in the S1 Text.

**Fig 7:**
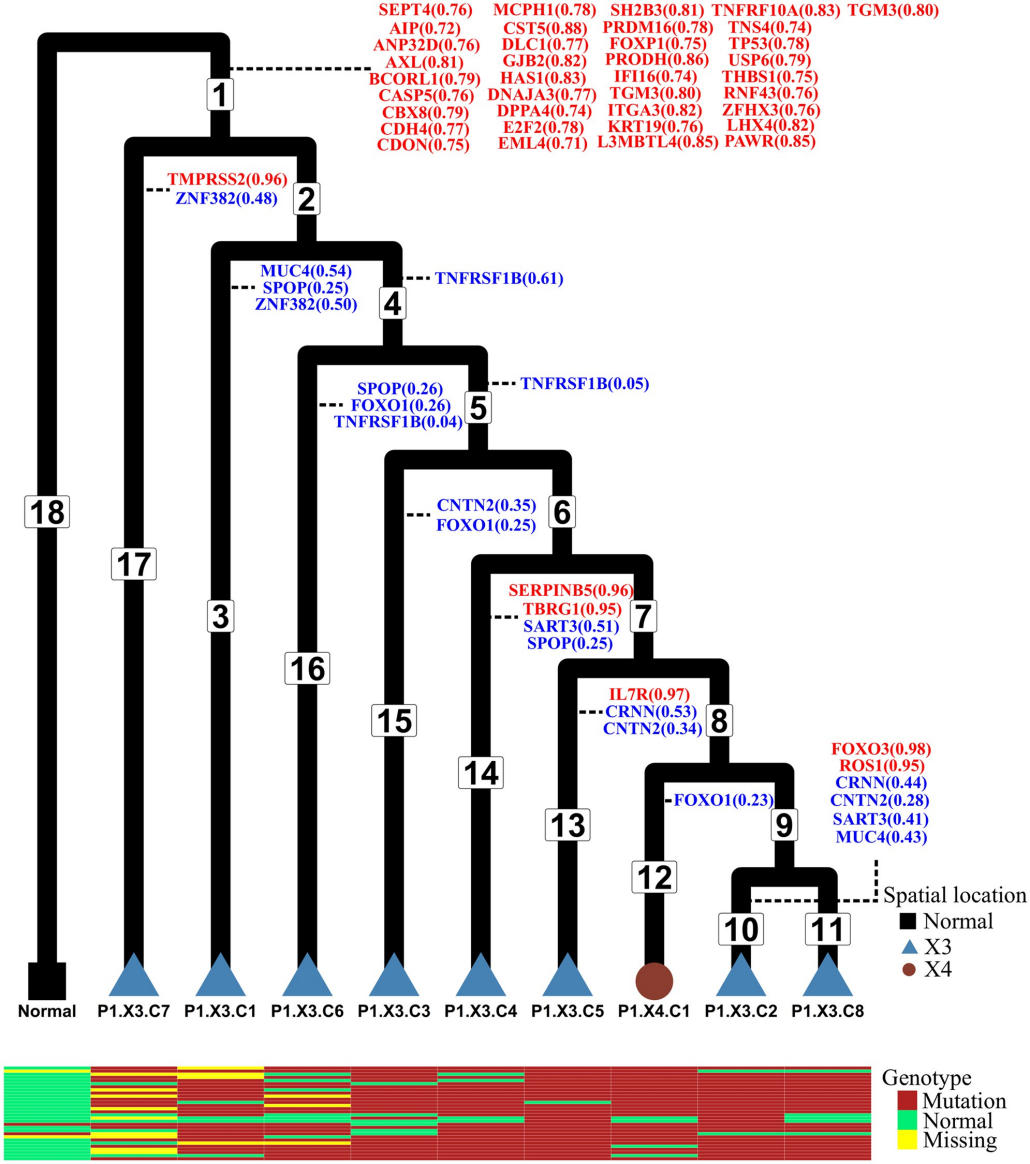
P1 tumor phylogenetic tree and inferred temporal order of the mutations. The normal cell is set as the outgroup. There are 18 branches in this tree. We do not assume the molecular clock when estimating the branch lengths. Branch lengths in this figure are not drawn to scale. The color and tip shape represent the spatial locations of the samples (normal tissue, location X3 or location X4; see [31]). The temporal order of the mutations is annotated on the branches of the tree. Mutations with very strong signals (probability of occurring on one branch is greater than 0.7) are highlighted in red, while mutations with moderate signals (probabilities that sum to more than 0.7 on two or three branches) are highlighted in blue. Mutation data for 30 genes corresponding to the first 30 rows in Figs R and S in the S1 Text for each tip are shown in the heatmap matrix at the bottom.

**Results.** Fig 7 and Fig O in the S1 Text show the tumor evolutionary tree estimated for P1 and P2, respectively. In both tumor trees, the trunk connects the tumor clone to the normal clone. We annotate the genes on their inferred mutation branches. The uncertainty in the inferred mutation locations is highlighted in colors. Mutations with strong signal (defined to be a posterior probability larger than 0.7 that the mutation occurred on a single branch) are colored red, while mutations with moderate signal (defined to be a total posterior probability larger than 0.7 on two or three branches) are colored blue. Note that the posterior probability on a branch measures the support in the data under the model and prior distribution for the placement of the mutation on that branch. Mutations colored red are those for which the placement on a single branch is strongly supported. Mutations colored blue are those for which there is strong support for the mutation having occurred on one of the indicated branches. This means that the precise placement of the mutation can be confidently limited to the branches indicated.

We also compare the estimated posterior probability distributions for mutations of common cancer-associated genes for patients P1 and P2, which are used to construct credible sets and to measure the uncertainty of the inferred mutation order. Figs R to U in the S1 Text are the posterior probability distribution heatmaps for patients P1 and P2 with different prior distributions (larger or smaller variance).

Figs V to Y in the S1 Text show heatmaps of the estimated posterior probabilities for prostate cancer-specific genes for patients P1 and P2 with different prior distributions (larger or smaller variance). In agreement with the findings of Su et al. (2018), we find that mutation of *TP53*, which is commonly associated with tumor initiation in many cancers (see, e.g., Yu et al. (2014) [37]), is inferred to occur on the trunk of the tree with high probability in patient P1, but not on the trunk of the tumor tree of patient P2. Gene *ZFHX3* has a high probability of having mutated on the trunk of the tree in both patients. In addition, the heatmap for patient P1 shows strong signal that *FOXP1* mutates on the trunk of the tumor tree, while *BRCA2* has a high probability of having mutated on the trunk of the tree for patient P2. Comparing the heatmaps of common cancer-associated genes with the prostate cancer-specific genes, mutations inferred to have occurred on the trunk of the tree tend to be those that are common across cancer types, while mutations known to have high frequency within prostate cancer are generally found closer to the tips of the tree in both patients.

#### 2.3.2 Metastatic colorectal cancer data

**Data analysis.** The original study of Leung et al. (2017) [32] reported 16 and 36 SNVs for patients CRC1 and CRC2 after variant calling. The normal cells in each patient were merged into one normal sample and used as the outgroup. We used SiFit [16] to estimate each colorectal patient’s tumor phylogeny, including branch lengths.

Leung et al. [32] reported error probabilities of (*α*, *β*) = (0.0152, 0.0789) and (*α*, *β*) = (0.0174, 0.1256) for CRC1 and CRC2, respectively. For each patient, we used these values to specify the same prior distributions across all sites. For CRC1, we considered *α*|**S**_*i*_ ∼ *Beta*(0.0152, 0.9848) (larger variance) and *α*|**S**_*i*_ ∼ *Beta*(0.15, 9.85) (smaller variance); and *β*|**s**_*i*_ ∼ *Beta*(0.078, 0.922) (larger variance) and *β*|**S**_*i*_ ∼ *Beta*(0.78, 9.22) (smaller variance). For CRC2, we considered *α*|**S**_*i*_ ∼ *Beta*(0.0174, 0.9826) (larger variance) and *α*|**S**_*i*_ ∼ *Beta*(0.174, 9.826) (smaller variance); and *β*|**S**_*i*_ ∼ *Beta*(0.1256, 0.8744) (larger variance) and *β*|**S**_*i*_ ∼ *Beta*(1.256, 8.744) (smaller variance). The prior distributions for the transition rates for CRC1 and CRC2 were estimated by SiFit. As was found for the prostate cancer patients, the estimated probabilities did not vary substantially when we used prior distributions with small or large variance.

**Results.** The inferred tumor trees and mutation order are depicted in Figs P and Q in the S1 Text. The posterior probabilities of the inferred mutation locations are indicated with colors as for the prostate cancer data. Figs Z and AA in the S1 Text are heatmaps for the posterior probability distribution of each mutation for patients CRC1 and CRC2 with different priors. For patient CRC1, mutations in *APC*, *KRAS* and *TP53* were inferred to have been acquired on the trunk of the tumor phylogeny with high posterior probability, in agreement with Leung et al. [32] and in agreement with past studies. The studies of Fearon and Vogelstein [38] and Powell et al. [39] have shown that the mutation order of these genes appears to be fixed in initializing colorectal cancer, providing further support for our findings. In addition, we identified mutations specific to metastatic cells [32], with three (*ZNF521*, *TRRAP*, *EYS*) inferred to occur on branch 97 in Fig P in the S1 Text. Support is found for placement of *RBFOX1* and *GATA1* in regions of metastatic aneuploid cells of the tree. Each supported placement is on a branch that leads to a clade of metastatic aneuploid cells, indicating the association of such cells with these mutations.

For CRC2, we identified strong signals on branch 36 in Fig Q in the S1 Text for several genes reported by Leung et al. [32] that are shared by primary and metastatic cells, including driver mutations in *APC*, *NRAS*, *CDK4* and *TP53*. We also identified an independent lineage of primary diploid cells (colored in pink in Fig Q in the S1 Text) that evolved in parallel with the rest of the tumor with moderate to strong signals for mutations in *ALK*, *ATR*, *EPHB6*, *NR3C2* and *SPEN* and that did not share the mutations listed in the previous sentence. Our analysis agreed with that of Leung et al. [32] in that we identified the subsequent formation of independent metastatic lineages. For example, on branches 56 and 58 we found moderate support for mutations in *FUS*; and strong support for mutation on branch 136 in *HELZ* and branch 78 in *PRKCB*. Many of the genes showing weaker or moderate support for mutation in these metastatic lineages agree with those identified by Leung et al. [32]. A primary difference between our result and that of Leung et al. [32] is that we identify mutation in *NR4A3* and *FUS* to have occurred along a different metastatic lineage than the mutations in *TSHZ3* and *PRKCB*.

## 3 Discussion

Development of computational tools based on a phylogenetic framework for use in studying cancer evolution provides tremendous insight into the mechanisms that lead to ITH, especially the role of the temporal order of mutations in cancer progression. For example, Ortmann et al. [20] have shown differences in clinical features and the response to treatment for patients with different mutation orders, indicating that inference of the order in which mutations arise within an individual’s tumor have direct implications in clinical oncology, for both diagnostic applications in measuring the extent of ITH and targeted therapy. SCS data provide an unprecedented opportunity to estimate mutation order at the highest resolution. However, such data are subject to extensive technical errors that arise during the process of whole-genome amplification.

To analyze such data, we introduce MO, a novel Bayesian approach for reconstructing the ordering of mutational events from the imperfect mutation profiles of single cells. MO is designed to infer the temporal order of a collection of mutations of interest based on a phylogeny of cell lineages that allows modeling of the errors at each tip. MO can infer the mutation order that best fits single-cell data sets that are subject to technical noise, including ADO, false positive errors, and missing data. MO could be extended to work on clonal trees and models that include errors in observed data for multiple cells in a tip instead of a single cell. In addition, MO could be straightforwardly modified to account for the accelerated mutation rates common in late-stage cancers, to allow for back or parallel mutation, or to allow for variation in mutation rates across sites. MO also provides flexibility in choosing the evolutionary model, for which we develop a model of evolution that accounts for the effects of point mutations in single-cell data sets. MO is robust to variation in error rates and performs accurately even with a small number of single cells. Our simulation results indicate that as the number of informative single cells increases, MO provides more accurate estimation.

MO improves the accuracy of mutation order prediction by modeling both the evolutionary process within the tumor and the errors that arise during SCS data collection. Another important difference between MO and existing methods, such as SCITE [22] and SiFit [16], is the unique mechanism for quantifying uncertainty in the inferred order along the phylogeny. Options available within SCITE [22] allow for estimation of the posterior probability distribution across orders. SiFit [16], on the other hand, reports the mutation order without such information. In contrast, because MO uses a probabilistic model for inferring mutation locations along a tree, it is able to provide an estimate of uncertainty in the inferred locations conditioning on the tumor phylogeny, thus capturing a source of uncertainty that differs from what SCITE and SiFit provide.

MO performs accurately, as is evident from a comprehensive set of simulation studies that take into account different aspects of modern SCS data sets by examining a wide range of error probabilities, fractions of missing data, branch lengths, and numbers of cells in each tree. The simulation studies also demonstrate that MO outperforms the state-of-the-art methods, especially when the number of cells in the phylogeny is large. We also use MO to reconstruct the mutation order for data from prostate cancer patients and colorectal cancer patients, and MO is able to accurately reconstruct the mutation order that has been reported to be important in cancer progression. MO is robust to the technical errors that arise during whole-genome amplification. MO is able to not only provide insight into the ordering of cancer-associated mutations, but also the level of certainty in the order. MO does not provide estimates of transition rates and error probabilities, but rather integrates over uncertainty in these parameters.

As SCS data collection becomes more advanced, enabling hundreds of cells to be analyzed in parallel at reduced cost and increased throughput, MO is poised to analyze the resulting large-scale data sets to make meaningful inference of the mutation order during tumor progression for individual patients. MO thus represents an important step forward in understanding the role of mutation order in cancer evolution and as such may have important translational applications for improving cancer diagnosis, treatment, and personalized therapy. Once the associations between inferred mutation order and clinical outcomes are established, future research can explore the cause of clinical outcomes given specific mutation orders with the goal of developing novel targeted treatments. This will allow clinical providers to make decisions concerning treatment based on the mutation landscapes of patients. Although the current study focuses on cancer, MO can potentially also be applied to single-cell mutation profiles from a wide variety of fields, including immunology, neurobiology, and microbiology. These applications are expected to provide new insights into our understanding of cancer and other human diseases.

## 4 Methods

### 4.1 MO overview

The input mutation data of MO can be either binary or ternary. For binary data, we define 0 and 1 as the absence of mutation and presence of mutation, while for ternary data, 0, 1 and 2 represent the homozygous reference (normal), heterozygous (mutation present) and homozygous non-reference (mutation present) genotypes, respectively. MO estimates mutation order with models at two levels in a Bayesian framework: the mutational process within the tumor, and the errors that arise during SCS data collection. MO provides a unique means to quantify the uncertainty in the inferred mutation order along a tumor phylogeny.

### 4.2 Somatic mutation process

#### Binary genotype data

For binary genotype data, the mutation process can be modeled by the 2 × 2 transition rate matrix

$$\begin{array}{c} \;\;\;0\;1\\ Q_{\lambda }=\begin{array}{cc} \begin{array}{c} 0\\ 1 \end{array} & \left(\begin{array}{cc} -\lambda & \lambda \\ 0 & 0 \end{array}\right) \end{array}, \end{array}$$ (2)

where λ denotes the instantaneous transition rate per genomic site. The transition probability matrix *P*(*t*) along a branch of length *t* is then computed by matrix exponentiation of the product of $Q_{\lambda }$ and the branch length *t*, which gives

$$\begin{array}{c} \;\;0\;\;1\;\;\;\;\;0\;\;\;\;\;1\\ P\left(t\right)=\begin{array}{cc} \begin{array}{c} 0\\ 1 \end{array} & \left(\begin{array}{cc} P_{00}\left(t\right) & P_{01}\left(t\right)\\ P_{10}\left(t\right) & P_{11}\left(t\right) \end{array}\right) \end{array}=\begin{array}{cc} \begin{array}{c} 0\\ 1 \end{array} & \left(\begin{array}{cc} \text{exp}\left(-\lambda t\right) & 1-\text{exp}\left(-\lambda t\right)\\ 0 & 1 \end{array}\right) \end{array}. \end{array}$$ (3)

Note that *P*_01_(*t*) is the probability that mutation *i* is acquired along a branch of length *t*. Under this model and recalling that each mutation evolves independently along different branches in T, the marginal probability that mutation *i* is acquired on branch *x* ∈ *E*, denoted by $P(B_{i}=x|T,Q_{\lambda })$, is thus given by

$$\begin{array}{c} P\left(B_{i}=x|T,Q_{\lambda }\right)=\frac{\left[\prod_{B\in [E\backslash \left(x\cup E^{x}\left(w\right)\right)]}P_{00}\left(t_{B}\right)]P_{01}\left(t_{x}\right)[\prod_{B\in E^{x}\left(w\right)}P_{11}\left(t_{B}\right)\right]}{\sum_{z\in E}([\prod_{B\in [E\backslash \left(z\cup E^{z}\left(h\right)\right)]}P_{00}\left(t_{B}\right)]P_{01}\left(t_{z}\right)[\prod_{B\in E^{z}\left(h\right)}P_{11}\left(t_{B}\right)])}, \end{array}$$ (4)

where *t*_*B*_ is length of branch *B*. In the numerator, the first term is a product of probabilities over all branches without the mutation, the second term is the probability that the mutation is acquired on branch *x*, and the third term is a product of probabilities over all branches with the mutation, i.e., all branches in *E*^*x*^(*w*). The denominator is needed to create a valid probability distribution over all possible branches, and is obtained by summing the numerator over all valid branches *z* ∈ *E*. The $P(B_{i}=x|T,Q_{\lambda })$ term is normalized by the denominator because we exclude two possibilities: a mutation is not acquired on any branch in T, or a mutation is acquired more than once on different branches in T.

As an example, Fig 2C depicts the observed and true binary genotype for mutation *i* = 1 shown in Fig 2A and 2B. The set of branches is *E* = {*e*_1_, …, *e*_8_} and the corresponding set of branch lengths would be **t** = {*t*_1_, …, *t*_8_}. If mutation *i* is acquired on branch *e*_1_, the cell descending along branch *e*_8_ will not carry the mutation, while those descending from the blue branches would carry this mutation. The marginal probability that mutation *i* = 1 is acquired on branch *e*_1_ would be proportional to its numerator, i.e., $P\left(B_{1}=e_{1}|T,Q_{\lambda }\right)\propto P_{00}\left(t_{8}\right)P_{01}\left(t_{1}\right)\left[P_{11}\left(t_{2}\right)P_{11}\left(t_{3}\right)P_{11}\left(t_{4}\right)P_{11}\left(t_{5}\right)P_{11}\left(t_{6}\right)P_{11}\left(t_{7}\right)\right]$.

#### Ternary genotype data

The mutation model for ternary data is complex and includes three possible ways that mutation *i* occurs on a branch *x* in T:

1. The status of mutation *i* transitions from 0 → 1 on a branch *x* and there is no further mutation at this genomic site in T.
2. The status of mutation *i* transitions directly from 0 → 2 on a branch *x* in T.
3. The status of mutation *i* transitions from 0 → 1 on a branch *x* and then transitions from 1 → 2 on a branch *y* ∈ *E*^*x*^(*w*) in T.

We let *B*_*i*_ be the location at which mutation *i* occurs, $B_{i}^{0\to 1}$ would be the branch on which mutation status transitions from 0 to 1, $B_{i}^{0\to 2}$ is the branch on which mutation status transitions from 0 to 2, and $B_{i}^{1\to 2}$ is the branch on which mutation status transitions from 1 to 2. If the mutation *i* occurs on branch *x*, all cells in *C*^*x*^(*w*) will carry 1 or 2 mutations. In other words, *G*_*ij*_ = 1 or 2 for all *C*_*j*_ ∈ *C*^*x*^(*w*) and *G*_*ij*_ = 0 for all *C*_*j*_ ∈ *C*∖*C*^*x*^(*w*). We define the transition rate matrix $Q_{\lambda }$ as

$$\begin{array}{c} \;\;\;\;0\;\;\;1\;\;2\\ Q_{\lambda }=\begin{array}{cc} \begin{array}{c} 0\\ 1\\ 2 \end{array} & \left(\begin{array}{ccc} -\left(\lambda_{1}+\lambda_{1}\lambda_{2}\right) & \lambda_{1} & \lambda_{1}\lambda_{2}\\ 0 & -\lambda_{2} & \lambda_{2}\\ 0 & 0 & 0 \end{array}\right) \end{array}, \end{array}$$ (5)

where λ_1_ and λ_2_ denote the instantaneous transition rates per genomic site of the transitions 0 → 1 and 1 → 2, respectively. Studies have provided evidence that direct mutation of 0 → 2 at rate λ_1_ λ_2_ is possible in principle, although it is extremely rare [26]. If λ_2_ is 0 in Expression (5), the model will be reduced to the infinite sites diploid model. The transition probability matrix $P\left(t\right)=\text{exp}\left(Q_{\lambda }t\right)$ is then given by

$$\begin{array}{c} 0\;\;\;\;\;\;\;\;\;\;1\;\;\;\;\;\;\;\;\;\;\;\;\;2\\ P\left(t\right)=\begin{array}{cc} \begin{array}{c} 0\\ 1\\ 2 \end{array} & \left(\begin{array}{ccc} \;\text{exp}\;\left(-\left(\lambda_{1}+\lambda_{1}\lambda_{2}\right)t\right) & \frac{\lambda_{1}\left(\;\text{exp}\;\left(-\left(\lambda_{1}+\lambda_{1}\lambda_{2}\right)t\right)-\;\text{exp}\;\left(-\lambda_{2}t\right)\right)}{\lambda_{2}-\left(\lambda_{1}+\lambda_{1}\lambda_{2}\right)} & \frac{\left(\lambda_{1}+\lambda_{1}\lambda_{2}\right)\;\text{exp}\;\left(-\left(\lambda_{1}+\lambda_{1}\lambda_{2}\right)t\right)+\lambda_{1}\;\text{exp}\;\left(-\lambda_{2}t\right)}{\lambda_{2}-\left(\lambda_{1}+\lambda_{1}\lambda_{2}\right)}+1\\ 0 & \;\text{exp}\;\left(-\lambda_{2}t\right) & 1-\;\text{exp}\;\left(-\lambda_{2}t\right)\\ 0 & 0 & 1 \end{array}\right) \end{array}. \end{array}$$ (6)

The marginal probability that mutation *i* occurs on branch *x* ∈ *E* for the three possible conditions is thus given by

$$\begin{array}{c} \begin{array}{c} P\left(B_{i}^{0\to 1}=x|T,Q_{\lambda }\right)=\frac{Q(B_{i}^{0\to 1}=x)}{\sum_{z_{1}\in E}\left[Q\left(B_{i}^{0\to 1}=z_{1}\right)+Q\left(B_{i}^{0\to 2}=z_{1}\right)+\sum_{z_{2}}Q\left(B_{i}^{0\to 1}=z_{1},B_{i}^{1\to 2}=z_{2}\right)\right]}, \end{array} \end{array}$$ (7)

$$\begin{array}{c} \begin{array}{c} P\left(B_{i}^{0\to 2}=x|T,Q_{\lambda }\right)=\frac{Q(B_{i}^{0\to 2}=x)}{\sum_{z_{1}\in E}\left[Q\left(B_{i}^{0\to 1}=z_{1}\right)+Q\left(B_{i}^{0\to 2}=z_{1}\right)+\sum_{z_{2}}Q\left(B_{i}^{0\to 1}=z_{1},B_{i}^{1\to 2}=z_{2}\right)\right]}, \end{array} \end{array}$$ (8)

$$\begin{array}{c} \begin{array}{cc} P(B_{i}^{0\to 1}=x,B_{i}^{1\to 2}= & y|T,Q_{\lambda })=\\ & \frac{Q(B_{i}^{0\to 1}=x,B_{i}^{1\to 2}=y)}{\sum_{z_{1}\in E}\left[Q\left(B_{i}^{0\to 1}=z_{1}\right)+Q\left(B_{i}^{0\to 2}=z_{2}\right)+\sum_{z_{2}}Q\left(B_{i}^{0\to 1}=z_{1},B_{i}^{1\to 2}=z_{2}\right)\right]},\; \end{array} \end{array}$$ (9)

where

$$\begin{array}{c} \begin{array}{c} Q\left(B_{i}^{0\to 1}=x\right)=[\prod_{B\in [E\backslash \left(x\cup E^{x}\left(w\right)\right)]}P_{00}\left(t_{B}\right)]P_{01}\left(t_{x}\right)[\prod_{B\in E^{x}\left(w\right)}P_{11}\left(t_{B}\right)], \end{array} \end{array}$$ (10)

$$\begin{array}{c} \begin{array}{c} Q\left(B_{i}^{0\to 2}=x\right)=[\prod_{B\in [E\backslash \left(x\cup E^{x}\left(w\right)\right)]}P_{00}\left(t_{B}\right)]P_{02}\left(t_{x}\right)[\prod_{B\in E^{x}\left(w\right)}P_{22}\left(t_{B}\right)], \end{array} \end{array}$$ (11)

$$\begin{array}{c} \begin{array}{cc} Q(B_{i}^{0\to 1}=x,B_{i}^{1\to 2}=y)= & [\prod_{B\in [E\backslash \left(x\cup E^{x}\left(w\right)\right)]}P_{00}\left(t_{B}\right)]P_{01}\left(t_{x}\right)\\ & \left[\prod_{B\in [E^{x}\left(w\right)\backslash \left(y\cup E^{y}\left(b\right)\right)]}P_{11}\left(t_{B}\right)\right]\\ & P_{12}\left(t_{y}\right)[\prod_{B\in E^{y}\left(b\right)}P_{22}\left(t_{B}\right)]. \end{array} \end{array}$$ (12)

We normalize the marginal probabilities to exclude scenarios in which mutations are acquired more than once or in which mutations are not acquired in T. As an example, Fig A in the S1 Text depicts the same mutation as in Fig 2, but considers ternary data, leading to the following:

1. The marginal probability that mutation *i* transitions from 0 → 1 on branch *e*_1_ is $P\left(B_{i}^{0\to 1}=e_{1}|T,Q_{\lambda }\right)\propto P_{00}\left(t_{8}\right)P_{01}\left(t_{1}\right)\left[P_{11}\left(t_{2}\right)P_{11}\left(t_{3}\right)P_{11}\left(t_{4}\right)P_{11}\left(t_{5}\right)P_{11}\left(t_{6}\right)P_{11}\left(t_{7}\right)\right]$.
2. The marginal probability that mutation *i* transitions from 0 → 2 on branch *e*_1_ is $P\left(B_{i}^{0\to 2}=e_{1}|T,Q_{\lambda }\right)\propto P_{00}\left(t_{8}\right)P_{02}\left(t_{1}\right)\left[P_{22}\left(t_{2}\right)P_{22}\left(t_{3}\right)P_{22}\left(t_{4}\right)P_{22}\left(t_{5}\right)P_{22}\left(t_{6}\right)P_{22}\left(t_{7}\right)\right]$.
3. The marginal probability that mutation *i* transitions from 0 → 1 on *e*_1_, and from 1 → 2 on *e*_3_ is $P\left(B_{i}^{0\to 1}=e_{1},B_{i}^{1\to 2}=e_{3}|T,Q_{\lambda }\right)\propto P_{00}\left(t_{8}\right)P_{01}\left(t_{1}\right)P_{11}\left(t_{2}\right)P_{12}\left(t_{3}\right)P_{22}\left(t_{4}\right)P_{22}\left(t_{5}\right)P_{22}\left(t_{6}\right)P_{22}\left(t_{7}\right)$.

The probability $P(B_{i}^{0\to 1}=e_{1},B_{i}^{1\to 2}=e_{3}|T,Q_{\lambda })$ is the marginal probability that two mutations at the same site along the genome occur on two branches *e*_1_ and *e*_3_, respectively. After the first mutation occurs on branch *e*_1_, the second mutation can occur on any branch except *e*_1_ and *e*_8_.

### 4.3 Quantification of SCS errors

#### Quantification of SCS errors for binary data

For binary data, if the true genotype is 0, we may observe a 1, which is a false positive error. If the true genotype is 1, we may observe a 0, which is a false negative error. The conditional probabilities of the observed data given the true genotype at genomic site *i* of cell *C*_*j*_ are

$$\begin{array}{c} \;\;\;\;\;S_{ij}=0\;S_{ij}=1\\ \begin{array}{c} N^{ij}=\begin{array}{cc} \begin{array}{c} G_{ij}=0\\ G_{ij}=1 \end{array} & \left(\begin{array}{cc} 1-\alpha_{ij} & \alpha_{ij}\\ \beta_{ij} & 1-\beta ij \end{array}\right) \end{array} \end{array}, \end{array}$$ (13)

where $N_{01}^{ij}=P\left(S_{ij}=1|G_{ij}=0\right)=\alpha_{ij}$, and other entries are defined similarly. Under the assumption that sequencing errors are independent, if mutation *i* is acquired on branch *x*, we can precisely quantify the effect of SCS technical errors for mutation *i* as

$$\begin{array}{c} P\left(S_{i}|B_{i}=x,T,N^{i}\right)=\prod_{j=1}^{J}P\left(S_{ij}|G_{ij}\right), \end{array}$$ (14)

where **N**^*i*^ = {**N**^*i*1^, …, **N**^*iJ*^}. Using the example in Fig 2, the error probability of the observed genotype conditioning on the mutation *i* = 1 occurring on branch *e*_1_ would be $P\left(S_{1}|B_{1}=e_{1},T,N^{1}\right)=N_{11}^{11}N_{10}^{12}N_{11}^{13}N_{10}^{14}N_{00}^{15}$, where **N**^1^ = {**N**^11^, …, **N**^15^} for this binary data example.

#### Quantification of SCS errors for ternary data

For ternary data, the conditional probabilities of the observed data given the true genotype are given by

$$\begin{array}{c} \;\;\;\;\;S_{ij}=0\;\;S_{ij}=1\;S_{ij}=2\\ \begin{array}{c} N^{ij}=\begin{array}{cc} \begin{array}{c} G_{ij}=0\\ G_{ij}=1\\ G_{ij}=2 \end{array} & \left(\begin{array}{ccc} 1-\alpha_{ij}-\alpha_{ij}\beta_{ij}/2 & \alpha_{ij} & \alpha_{ij}\beta_{ij}/2\\ \beta_{ij}/2 & 1-\beta_{ij} & \beta_{ij}/2\\ 0 & 0 & 1 \end{array}\right) \end{array} \end{array}, \end{array}$$ (15)

where $N_{01}^{ij}=P\left(S_{ij}=1|G_{ij}=0\right)=\alpha_{ij}$, and the other entries are defined similarly. Under the same assumptions as for binary genotype data, we can precisely quantify the effect of SCS technical errors as in Eq (14) if mutation *i* is acquired on branch *x*. Using the example in Fig A in the S1 Text, the error probabilities for the three possible ways that mutation *i* = 1 may arise on branch *e*_1_ are

1. The error probability under the condition that the true mutation transitions from 0 → 1 on branch *e*_1_ is $P\left(S_{1}|B_{i}^{0\to 1}=e_{1},T,N^{1}\right)=N_{12}^{11}N_{10}^{12}N_{11}^{13}N_{10}^{14}N_{00}^{15}$.
2. The error probability under the condition that the true mutation transitions from 0 → 2 on branch *e*_1_ is $P\left(S_{1}|B_{i}^{0\to 2}=e_{1},T,N^{1}\right)=N_{22}^{11}N_{20}^{12}N_{21}^{13}N_{20}^{14}N_{00}^{15}$.
3. The error probability under the condition that the true mutation transitions from 0 → 1 on branch *e*_1_, and transitions from 1 → 2 on branch *e*_3_ is $P\left(S_{1}|B_{i}^{0\to 1}=e_{1},B_{i}^{1\to 2}=e_{3},T,N^{1}\right)=N_{12}^{11}N_{20}^{12}N_{21}^{13}N_{20}^{14}N_{00}^{15}$.

And **N**^1^ = {**N**^11^, …, **N**^15^} for this ternary data example. The term $P(S_{1}|B_{i}^{0\to 1}=e_{1},B_{i}^{1\to 2}=e_{3},T,N^{1})$ gives the error probability for the case in which the two mutations at the same genomic site occur on branches *e*_1_ and *e*_3_.

### 4.4 Missing and ambiguous data

In real data, missing and ambiguous states are observed and must be taken into account. For each mutation *i*, we exclude cells with missing states, and a subtree $T_{i}$ from T is extracted. The number of tips *J*_*i*_ in subtree $T_{i}$ is less than or equal to *J*. Let *E*_*i*_ be the set of branches in subtree $T_{i}$. The probability that mutation *i* occurs on branch *x* is then given by $P\left(B_{i}=x|T,Q_{\lambda }\right)=P\left(B_{i}=x|T_{i},Q_{\lambda }\right)$, where $P(B_{i}=x|T_{i},Q_{\lambda })$ is computed based on branches in the subtree $T_{i}$, and $P(B_{i}=x|T_{i},Q_{\lambda })$ is 0 for those branches *x* ∈ *E*∖*E*_*i*_. We quantify the effect of the SCS technical errors as

$$\begin{array}{c} \begin{array}{cc} P(S_{i}|B_{i}=x,T,N^{i}) & =\prod_{j=1}^{J_{i}}(\sum_{S_{ijk}}w_{ijk}P\left(S_{ijk}|G_{ijk}\right)), \end{array} \end{array}$$ (16)

where *w*_*ijk*_ is the weight for each possible observed state at a mutation site. For a site with an observed state that is not missing or ambiguous, *w*_*ijk*_ is 1 for the observed state and 0 for all other states. For an ambiguous site, we can assign equal weight for each possible state, or we can assign weight based on sequencing information (e.g., sequence depth) or other biological characteristics.

### 4.5 Inferring the location of a mutation in T

Given the observed data matrix **S**, the tumor phylogenetic tree T, the error probability matrix **N** = {**N**^*ij*^|1 ≤ *i* ≤ *I*, 1 ≤ *j* ≤ *J*}, and the mutation process $Q_{\lambda }$, we can assign a posterior probability distribution $P(B_{i}|S,T,N,Q_{\lambda })$ to the location of mutation *i* using Bayes’ theorem,

$$\begin{array}{c} \begin{array}{cc} P(B_{i}=x|S,T,N,Q_{\lambda }) & =\frac{P\left(S_{i}|B_{i}=x,T_{i},N^{i}\right)P\left(B_{i}=x|T_{i},Q_{\lambda }\right)}{P(S_{i}|T_{i},N^{i},Q_{\lambda })}. \end{array} \end{array}$$ (17)

For mutation *i*, $P\left(B_{i}=x|S_{i},T_{i},N^{i},Q_{\lambda }\right)\propto P\left(S_{i}|B_{i}=x,T_{i},N^{i}\right)P\left(B_{i}=x|T_{i},Q_{\lambda }\right)$ and $P(B_{i}=x|S_{i},T_{i},N^{i},Q_{\lambda })$ is computed for all *x* in set *E*_*i*_. For example, there are 8 branches in the tree in Fig 2, so the branch on which mutation *i* = 1 occurs, *B*_1_, can be any of the 8 branches. For the binary example, the posterior probability that mutation *i* = 1 occurs on *e*_1_ is $P\left(B_{1}=e_{1}|S_{1},T_{1},N^{1},Q_{\lambda }\right)\propto P_{00}\left(t_{8}\right)P_{01}\left(t_{1}\right)\left[P_{11}\left(t_{2}\right)P_{11}\left(t_{3}\right)P_{11}\left(t_{4}\right)P_{11}\left(t_{5}\right)P_{11}\left(t_{6}\right)P_{11}\left(t_{7}\right)\right]\cdot N_{11}^{11}N_{10}^{12}N_{11}^{13}N_{10}^{14}N_{00}^{15}$. In this way, the posterior probability that the mutation occurs on each of the 8 branches can be computed, giving the probability distribution for the location of mutation *i* = 1, i.e. $P(B_{1}=x|S_{1},T_{1},N^{1},Q_{\lambda })$ for *x* ∈ {*e*_1_, …, *e*_8_}.

To summarize this probability distribution, we construct a (1 − *θ*) × 100% credible set for the location of mutation *i* as follows. First, the branches are ranked by their posterior probabilities, and then branches are added to the credible set in the order of decreasing posterior probability until the sum of their probabilities reaches (1 − *θ*). The number of branches in the credible set is informative about the level of certainty associated with the inferred location for the mutation. To obtain a point estimate, we pick the branch that maximizes the posterior probability, i.e., the maximum a posteriori (MAP) estimate. The MAP estimator for the location of mutation *i* is given by

$$\begin{array}{c} {\hat{B}}_{i_{MAP}}=\underset{B_{i}\in \left\{e_{1},\ldots ,e_{2J-2}\right\}}{\text{argmax}}\;P\left(B_{i}|S_{i},T,N^{i},Q_{\lambda }\right). \end{array}$$ (18)

For the example in Fig 2, the branch with the largest posterior probability is ${\hat{B}}_{1_{MAP}}$ for mutation *i* = 1.

### 4.6 Inferring the mutation order in T

We now consider the joint posterior probability distribution of the locations for the *I* mutations in the sample of *J* single cells. Based on the assumption of independence among the *I* mutations being considered, the posterior distribution for B is given by

$$\begin{array}{c} P\left(B|S,T,N,Q_{\lambda }\right)=\prod_{i=1}^{I}P\left(B_{i}|S_{i},T,N^{i},Q_{\lambda }\right), \end{array}$$ (19)

where **N**^*i*^ = {**N**^*i*1^, …, **N**^*iJ*^}. From this distribution, we can extract information on the ordering of mutations of interest. For example, if we are interested in the order of mutation *i* = 1 and mutation *i* = 2 in Fig 2, the joint posterior probability distribution that mutation *i* = 1 occurs on branch *x* ∈ *E* and mutation *i* = 2 occurs on branch *y* ∈ *E* can be used to find the probability that mutation *i* = 1 occurs earlier in the tree than mutation *i* = 2. Note that $P^{B_{1}=x,B_{2}=y}=P\left(B_{1}=x,B_{2}=y|S,T,N,Q_{\lambda }\right)=P\left(B_{1}=x|S,T,N,Q_{\lambda }\right)\cdot P\left(B_{2}=y|S,T,N,Q_{\lambda }\right)$. This joint distribution can be represented in a matrix given by

$$\begin{array}{c} \;\;\;B_{1}=e_{1}\;\;B_{1}=e_{2}\;\ldots \;B_{1}=e_{8}\\ \begin{array}{c} \begin{array}{cc} \begin{array}{c} B_{2}=e_{1}\\ B_{2}=e_{2}\\ \vdots \\ B_{2}=e_{8} \end{array} & \left(\begin{array}{cccc} P^{B_{1}=e_{1},B_{2}=e_{1}} & P^{B_{1}=e_{2},B_{2}=e_{1}} & \ldots & P^{B_{1}=e_{8},B_{2}=e_{1}}\\ P^{B_{1}=e_{1},B_{2}=e_{2}} & P^{B_{1}=e_{2},B_{2}=e_{2}} & \ldots & P^{B_{1}=e_{8},B_{2}=e_{2}}\\ \vdots & \vdots & \ddots & \vdots \\ P^{B_{1}=e_{1},B_{2}=e_{8}} & P^{B_{1}=e_{2},B_{2}=e_{8}} & \ldots & P^{B_{1}=e_{8},B_{2}=e_{8}} \end{array}\right) \end{array} \end{array}. \end{array}$$

Adding entries of the matrix for which branch *B*_1_ is earlier in the tree than branch *B*_2_ thus gives the probability that mutation 1 occurs before mutation 2. To measure the uncertainty of the ordering of the mutations, we rank all possible mutation orders by their posterior probabilities, and construct a (1 − *θ*) × 100% credible set by adding orders with decreasing probability until the sum exceeds 1 − *θ*. The MAP estimator for the order of *I* mutations is thus given by

$$\begin{array}{c} {\hat{B}}_{MAP}=\underset{B\in {\left\{e_{1},\ldots ,e_{2J-2}\right\}}^{I}}{\text{argmax}}\;P\left(B|S,T,N,Q_{\lambda }\right). \end{array}$$ (20)

### 4.7 Assessment of accuracy of point estimates in simulation study

We assess the performance of MO in three aspects in simulation study as below.

#### Location accuracy

We first evaluate how accurately MO can estimate the branch on which the mutation occurs. In each setting, we quantify the mutation location accuracy by

$$\begin{array}{c} \frac{\text{total}\;\text{number}\;\text{of}\;\text{mutations}\;\text{which}\;\text{have}\;\text{the}\;\text{correct}\;\text{inferred}\;\text{branch}}{\text{total}\;\text{number}\;\text{of}\;\text{mutations}}, \end{array}$$ (21)

where the inferred branch is correct if it is the same as the true mutation branch, and both the numerator and denominator are evaluated among the 100 trees in each setting.

#### Order accuracy

The second way to evaluate MO is to compare the order accuracy of all pairs of mutations among the 100 trees without considering if the branches are adjacent, and is measured by

$$\begin{array}{c} \frac{\text{number}\;\text{of}\;\text{all}\;\text{paired}\;\text{mutations}\;\text{with}\;\text{the}\;\text{correct}\;\text{inferred}\;\text{order}}{\text{number}\;\text{of}\;\text{all}\;\text{true}\;\text{paired}\;\text{mutations}}, \end{array}$$ (22)

where both the numerator and denominator are evaluated among the 100 trees.

#### Adjacent order accuracy

The third way to evaluate MO is to compare the adjacent order accuracy. In each simulated tree, if one pair of mutations are acquired on two adjacent branches, we compare if they have the same inferred mutation order on any two adjacent branches. In each setting, the adjacent order accuracy is measured by

$$\begin{array}{c} \frac{\text{number}\;\text{of}\;\text{all}\;\text{adjacent}\;\text{paired}\;\text{mutations}\;\text{with}\;\text{the}\;\text{correct}\;\text{inferred}\;\text{order}}{\text{number}\;\text{of}\;\text{all}\;\text{true}\;\text{adjacent}\;\text{paired}\;\text{mutations}}, \end{array}$$ (23)

where both the numerator and denominator are evaluated among the 100 trees.

#### Example

**Fig 8:**
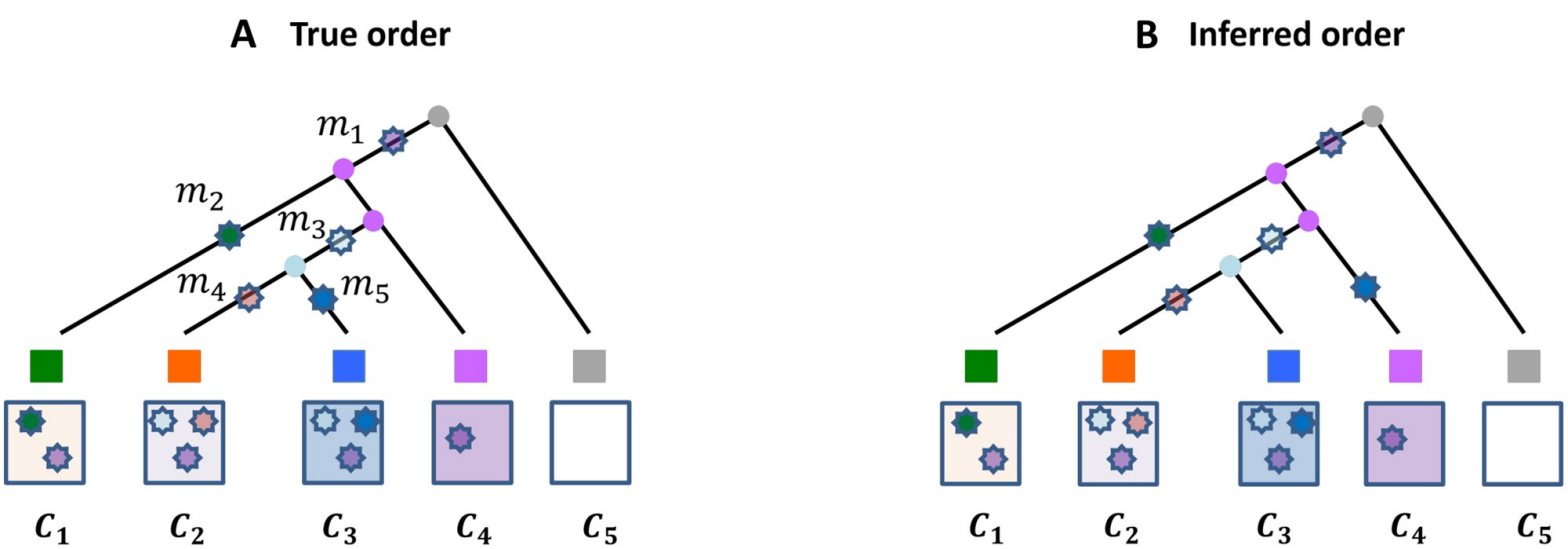
Example measures of accuracy (A) True mapping of mutations along a fixed phylogenetic tree; (B) Inferred mapping of mutations along the same phylogeny. The adjacent order accuracy is 2/3, while the order accuracy is 5/6.

Fig 8 provides an example of order accuracy and adjacent order accuracy. Note that for the true mapping of mutations onto the phylogeny (left), there are 3 pairs of ancestor and descendant mutations that happen on adjacent branches: (*m*_1_, *m*_2_), (*m*_3_, *m*_4_), and (*m*_3_, *m*_5_). In the inferred mapping of mutations onto the phylogeny (right), two pairs of mutations occur on adjacent branches:(*m*_1_, *m*_2_) and (*m*_3_, *m*_4_). The adjacent order accuracy is thus 2/3.

Similarly, there are 6 pairs of mutations that have ordered relationship regardless of whether they occur on adjacent branches for the true mapping of mutations onto the phylogeny: (*m*_1_, *m*_2_), (*m*_1_, *m*_3_), (*m*_1_, *m*_4_), (*m*_1_, *m*_5_), (*m*_3_, *m*_4_), and (*m*_3_, *m*_5_). In the inferred mapping of mutations, four pairs of mutations have the correct order: (*m*_1_, *m*_2_), (*m*_1_, *m*_3_), (*m*_1_, *m*_4_), (*m*_1_, *m*_5_), and (*m*_3_, *m*_4_). Thus, the adjacent order accuracy is 5/6.

### 4.8 Incorporating uncertainty in the parameters

Both the binary and ternary models involve the use of transition rates. While some information about mutation rates is available in the literature, we incorporate uncertainty in these rates by assigning prior distributions. Specifically, we assume gamma priors to describe the distribution of transition rate for each genomic site, λ_1_ ∼ *Gamma*(λ_11_, λ_12_) and λ_2_ ∼ *Gamma*(λ_21_, λ_22_). We assume beta priors for the false positive probability *α*_*ij*_ and the false negative probability *β*_*ij*_ for each genomic site in each cell, $\alpha_{ij}∼Beta\left(\alpha_{ij}^{1},\alpha_{ij}^{2}\right)$ and $\beta_{ij}∼Beta\left(\beta_{ij}^{1},\beta_{ij}^{2}\right)$. The parameters in these prior distributions are specified using information obtained during sequencing in the case of *α*_*ij*_ and *β*_*ij*_, and using information from the literature for the mutation rate parameters (λ_1_ and λ_2_). To carry out inference, we use Monte Carlo integration to integrate over the distributions of these parameters.

## Supporting information

S1 Text The Supporting information includes details about computational time and scalability, description of an alternative modeling strategy, and supplemental figures and tables. **Fig A. Three possible ways that a mutation may arise on branch *e*_1_.** In (a), the status of mutation *i* = 1 transitions from 0 → 1 on branch *e*_1_ and there is no further mutation at this genomic site. In (b), the status of mutation *i* = 1 transitions directly from 0 → 2 on branch *e*_1_. In (c), the status of mutation *i* = 1 transitions from 0 → 1 on a branch *e*_1_ and then transitions from 1 → 2 on branch *e*_3_. **Fig B. Adjacent order accuracy in scenarios 1 and 2 of MO, SCITE, SiFit and SPhyR when there are 40 mutations.** Each panel includes the results from the specific type of genotype and missing data percentage. In each panel, red, gray, blue, green and yellow colors correspond to MO with the true tree, MO with the estimated tree, SCITE, SiFit and SPhyR, respectively. **Fig C. Adjacent order accuracy in scenarios 1 and 2 of MO, SCITE, SiFit and SPhyR when there are 80 mutations.** Each panel includes the results from the specific type of genotype and missing data percentage. In each panel, red, gray, blue, green and yellow colors correspond to MO with the true tree, MO with the estimated tree, SCITE, SiFit and SPhyR, respectively. **Fig D. Order accuracy in scenarios 1 and 2 of MO, SCITE, SiFit and SPhyR when there are 40 mutations.** Each panel includes the results from the specific type of genotype and missing data percentage. In each panel, red, gray, blue, green and yellow colors correspond to MO with the true tree, MO with the estimated tree, SCITE, SiFit and SPhyR, respectively. **Fig E. Order accuracy in scenarios 1 and 2 of MO, SCITE, SiFit and SPhyR when there are 80 mutations.** Each panel includes the results from the specific type of genotype and missing data percentage. In each panel, red, gray, blue, green and yellow colors correspond to MO with the true tree, MO with the estimated tree, SCITE, SiFit and SPhyR, respectively. **Fig F. Adjacent order accuracy in scenarios 5 and 6 of MO, SCITE, SiFit and SPhyR when there are 20 mutations.** Mutations are simulated under the mutation process defined in Section 4.2. Each panel includes the results from the specific type of genotype and missing data percentage. In each panel, red, gray, blue, green and yellow colors correspond to MO with the true tree, MO with the estimated tree, SCITE, SiFit and SPhyR, respectively. **Fig G. Adjacent order accuracy in scenarios 5 and 6 of MO, SCITE, SiFit and SPhyR when there are 40 mutations.** Mutations are simulated under the mutation process defined in Section 4.2. Each panel includes the results from the specific type of genotype and missing data percentage. In each panel, red, gray, blue, green and yellow colors correspond to MO with the true tree, MO with the estimated tree, SCITE, SiFit and SPhyR, respectively. **Fig H. Adjacent order accuracy in scenarios 5 and 6 of MO, SCITE, SiFit and SPhyR when there are 80 mutations.** Mutations are simulated under the mutation process defined in Section 4.2. Each panel includes the results from the specific type of genotype and missing data percentage. In each panel, red, gray, blue, green and yellow colors correspond to MO with the true tree, MO with the estimated tree, SCITE, SiFit and SPhyR, respectively. **Fig I. Order accuracy in scenarios 5 and 6 of MO, SCITE, SiFit and SPhyR when there are 20 mutations.** Mutations are simulated under the mutation process defined in Section 4.2. Each panel includes the results from the specific type of genotype and missing data percentage. In each panel, red, gray, blue, green and yellow colors correspond to MO with the true tree, MO with the estimated tree, SCITE, SiFit and SPhyR, respectively. **Fig J. Order accuracy in scenarios 5 and 6 of MO, SCITE, SiFit and SPhyR when there are 40 mutations.** Mutations are simulated under the mutation process defined in Section 4.2. Each panel includes the results from the specific type of genotype and missing data percentage. In each panel, red, gray, blue, green and yellow colors correspond to MO with the true tree, MO with the estimated tree, SCITE, SiFit and SPhyR, respectively. **Fig K. Order accuracy in scenarios 5 and 6 of MO, SCITE, SiFit and SPhyR when there are 80 mutations.** Mutations are simulated under the mutation process defined in Section 4.2. Each panel includes the results from the specific type of genotype and missing data percentage. In each panel, red, gray, blue, green and yellow colors correspond to MO with the true tree, MO with the estimated tree, SCITE, SiFit and SPhyR, respectively. **Fig L. Location accuracy in scenarios 7 and 8 of MO and SiFit.** Mutations were simulated under the finite sites assumption and error rates, shown on the x-axis, are assumed to be equal. **Fig M. Adjacent order accuracy (top) and order accuracy (bottom) in scenario 9 with 500 cells (left column) or 1,000 cells (right column).** We did not obtain results for SCITE for the setting with 10,000 sites even after running each replicate for several days. MO has higher adjacent order accuracy and order accuracy than SiFit and SPhyR, as well as over SCITE for datasets with 1,000 sites **Fig N. Speed comparison.** The speed of the different methods for the simulation data in Scenario 9 is compared. (a) Each plotting symbol represents a different number of mutations. The x-axis is the probability of error, *β*. The y-axis is the computation time for 100 replicates in each setting. Note the logarithmic time scale on the y-axis. (b) Each plotting symbol represents a different genotype. The x-axis is the number of mutations in scenario 9. The y-axis is the computation time for 100 replicates in each setting. Note the logarithmic time scale on the y-axis. **Fig O. P2 tumor phylogenetic tree and inferred temporal order of the mutations.** Normal.R0 and Normal.L0 are normal cells from the right side and the left side of tissue, respectively. There are 18 branches in this tree. We do not assume the molecular clock when estimating the branch lengths, and branch lengths in this figure are not drawn to scale. The color and tip shape represent the spatial locations of the samples (normal tissue, left-side locations L3 or L4, or right-side location R3; see [31]). The temporal order of the mutations is annotated on the branches of the tree. Mutations with very strong signals (probability of occurring on one branch is greater than 0.7) are marked in red, while mutations with moderate signals (probabilities that sum to more than 0.7 on two or three branches) are marked in blue. The probability of a mutation on the indicated branch is annotated in the parentheses after each gene. Mutation data for 30 genes corresponding to the first 30 rows in Figs T and U for each tip are shown in the heatmap matrix at the bottom. **Fig P. CRC1 tumor phylogenetic tree and inferred temporal order of the mutations.** The color and tip shape represent the spatial locations of the samples (Normal—normal tissue; PA—primary aneuploid; PD—primary diploid; MA—metastatic aneuploid; MD—metastatic diploid; see [32]). The temporal order of the mutations is annotated on the branches of the tree. Mutations with very strong signals (probability of occurring on one branch greater than 0.7) are marked in red, while genes with moderate signals (probabilities that sum to more than 0.7 on two or three branches) are marked in blue. The probability of a mutation on the indicated branch is annotated in the parentheses after each gene. The branch lengths are not scaled. Mutation data for the 16 genes corresponding to each tip are shown in the heatmap matrix at the bottom. **Fig Q. CRC2 tumor phylogenetic tree and inferred temporal order of the mutations.** The color and tip shape represent the spatial locations of the samples (Normal—normal tissue; PA—primary aneuploid; PD—primary diploid; MA—metastatic aneuploid; MD—metastatic diploid; see [32]). The temporal order of the mutations is annotated on the branches of the tree. Mutations with very strong signals (probability of occurring on one branch greater than 0.7) are marked in red, while mutations with moderate signals (probabilities that sum to more than 0.7 on two or three branches) are marked in blue. The probability of a mutation on the indicated branch is annotated in the parentheses after each gene. The branch lengths are not scaled. Mutation data for the 36 genomic sites corresponding to each tip are shown in the heatmap matrix at the bottom. **Fig R. Heatmap of posterior probabilities that each mutation occurs on each branch for common tumor suppressor genes or oncogenes for prostate cancer patient P1 with prior distributions that have larger variances.** Color indicates the magnitude of the probability, with red indicating probability close to 1 and blue indicating probability close to 0. For P1, prior of *α* is set as *α* ∼ *Beta*(0.29, 0.71) (larger variance). The prior of *β* is set as *β* ∼ *Beta*(0.02, 0.98) (larger variance). Distribution of transition rate λ_1_ is set as λ_1_ ∼ *Gamma*(2, 5.0 × 10^−8^) (larger variance). Distribution of transition rate λ_2_ is set as λ_2_ ∼ *Gamma*(2, 5.0 × 10^−3^) (larger variance). **Fig S. Heatmap of posterior probabilities that each mutation occurs on each branch for common tumor suppressor genes or oncogenes for prostate cancer patient P1 with prior distributions that have smaller variances.** Color indicates the magnitude of the probability, with red indicating probability close to 1 and blue indicating probability close to 0. For P1, prior of *α* is set as *α* ∼ *Beta*(2.9, 7.1) (smaller variance). The prior of *β* is set as *β* ∼ *Beta*(0.2, 9.8) (smaller variance). Distribution of transition rate λ_1_ is set as λ_1_ ∼ *Gamma*(5, 2.0 × 10^−8^) (smaller variance). Distribution of transition rate λ_2_ is set as λ_2_ ∼ *Gamma*(5, 2.0 × 10^−3^) (smaller variance). **Fig T. Heatmap of posterior probabilities that each mutation occurs on each branch for common tumor suppressor genes or oncogenes for prostate cancer patient P2 with prior distributions that have larger variances.** Color indicates the magnitude of the probability, with red indicating probability close to 1 and blue indicating probability close to 0. For P2, prior of *α* is set as *α* ∼ *Beta*(0.31, 0.69) (larger variance). The prior of *β* is set as *β* ∼ *Beta*(0.02, 0.98) (larger variance). Prior distributions for the transition rate parameters are as in Fig R (larger variance). **Fig U. Heatmap of posterior probabilities that each mutation occurs on each branch for common tumor suppressor genes or oncogenes for prostate cancer patient P2 with prior distributions that have smaller variances.** Color indicates the magnitude of the probability, with red indicating probability close to 1 and blue indicating probability close to 0. For P2, prior of *α* is set as *α* ∼ *Beta*(3.1, 6.9) (smaller variance). The prior of *β* is set as *β* ∼ *Beta*(0.2, 9.8) (smaller variance). Prior distributions for the transition rate parameters are as in Fig S (smaller variance). **Fig V. Heatmap of posterior probabilities that each mutation occurs on each branch for prostate cancer-specific genes for prostate cancer patient P1 with prior distributions that have large variances.** This heatmap is for the prostate cancer-specific genes. Color indicates the magnitude of the probability, with red indicating probability close to 1 and blue indicating probability close to 0. Prior distributions for the parameters are as in Fig R (larger variance). **Fig W. Heatmap of posterior probabilities that each mutation occurs on each branch for prostate cancer-specific genes for prostate cancer patient P1 with prior distributions that have small variances.** Color indicates the magnitude of the probability, with red indicating probability close to 1 and blue indicating probability close to 0. Prior distributions for the parameters are as in Fig S (smaller variance). **Fig X. Heatmap of posterior probabilities that each mutation occurs on each branch for prostate cancer-specific genes for prostate cancer patient P2 with prior distributions that have large variances.** Color indicates the magnitude of the probability, with red indicating probability close to 1 and blue indicating probability close to 0. Prior distributions for the parameters are as in Fig T (larger variance). **Fig Y. Heatmap of posterior probabilities that each mutation occurs on each branch for prostate cancer-specific genes for prostate cancer patient P2 with prior distributions that have small variances.** Color indicates the magnitude of the probability, with red indicating probability close to 1 and blue indicating probability close to 0. Prior distributions for the parameters are as in Fig U (smaller variance). **Fig Z. Heatmap of probabilities on each branch for mutations in patient CRC1 and CRC2 with prior distributions that have large variances.** Color indicates the magnitude of the probability, with red indicating probability close to 1 and blue indicating probability close to 0. Prior distributions for the parameters are set with larger variance. **Fig AA. Heatmap of probabilities on each branch for mutations in patient CRC1 and CRC2 with prior distributions that have small variances.** Color indicates the magnitude of the probability, with red indicating probability close to 1 and blue indicating probability close to 0. Prior distributions for the parameters are set with smaller variance. **Table A. Location accuracy of MO in scenario 1.** Each cell corresponds to unique *α*, *β*, type of genotype and missing data percentage. **Table B. Location accuracy of MO in scenario 2.** Each cell corresponds to unique *α*, *β*, type of genotype and missing data percentage. **Table C. Order accuracy of MO in scenario 1.** Each cell corresponds to unique *α*, *β*, type of genotype and missing data percentage. **Table D. Order accuracy of MO in scenario 2.** Each cell corresponds to unique *α*, *β*, type of genotype and missing data percentage. **Table E. Adjacent order accuracy of MO in scenario 1.** Each cell corresponds to unique *α*, *β*, type of genotype and missing data percentage. **Table F. Adjacent order accuracy of MO in scenario 2.** Each cell corresponds to unique *α*, *β*, type of genotype and missing data percentage. **Table G. Credible set accuracy of MO in scenario 1.** Each cell corresponds to unique *α*, *β*, type of genotype and missing data percentage. **Table H. Credible set accuracy of MO in scenario 2.** Each cell corresponds to unique *α*, *β*, type of genotype and missing data percentage. **Table I. Location accuracy of MO when the mutation process is modeled using the multinomial distribution in scenario 1 and 2 for binary data.** Each cell corresponds to a unique *α*, *β*, number of mutations, missing data percentage and scenario setting.
(PDF)
