# Supplementary material for: A phylogenetic approach to inferring the order in which mutations arise during cancer progression
Source: PLoS Comput Biol. 2022 Dec 2;18(12):e1010560. doi: 10.1371/journal.pcbi.1010560 (PMC9750018; doi:10.1371/journal.pcbi.1010560)
Supplement: S1 Text — Fig A. Three possible ways that a mutation may arise on branch e1. In (a), the status of mutation i = 1 transitions from 0 → 1 on branch e1 and there is no further mutation at this genomic site. In (b), the status of mutation i = 1 transitions directly from 0 → 2 on branch e1. In (c), the status of mutation i = 1 transitions from 0 → 1 on a branch e1 and then transitions from 1 → 2 on branch e3. Fig B. Adjacent order accuracy in scenarios 1 and 2 of MO, SCITE, SiFit and SPhyR when there are 40 mutations. Each panel includes the results from the specific type of genotype and missing data percentage. In each panel, red, gray, blue, green and yellow colors correspond to MO with the true tree, MO with the estimated tree, SCITE, SiFit and SPhyR, respectively. Fig C. Adjacent order accuracy in scenarios 1 and 2 of MO, SCITE, SiFit and SPhyR when there are 80 mutations. Each panel includes the results from the specific type of genotype and missing data percentage. In each panel, red, gray, blue, green and yellow colors correspond to MO with the true tree, MO with the estimated tree, SCITE, SiFit and SPhyR, respectively. Fig D. Order accuracy in scenarios 1 and 2 of MO, SCITE, SiFit and SPhyR when there are 40 mutations. Each panel includes the results from the specific type of genotype and missing data percentage. In each panel, red, gray, blue, green and yellow colors correspond to MO with the true tree, MO with the estimated tree, SCITE, SiFit and SPhyR, respectively. Fig E. Order accuracy in scenarios 1 and 2 of MO, SCITE, SiFit and SPhyR when there are 80 mutations. Each panel includes the results from the specific type of genotype and missing data percentage. In each panel, red, gray, blue, green and yellow colors correspond to MO with the true tree, MO with the estimated tree, SCITE, SiFit and SPhyR, respectively. Fig F. Adjacent order accuracy in scenarios 5 and 6 of MO, SCITE, SiFit and SPhyR when there are 20 mutations. Mutations are simulated under the mutation p [file pcbi.1010560.s001.pdf]

Supplementary material to "A Phylogenetic Approach to Inferring the Order in Which Mutations Arise during Cancer Progression"

Yuan Gao[1], Jeff Gaither[2], Julia Chifman[3], Laura Kubatko[4,5*],

1 Division of Biostatistics, The Ohio State University, Columbus, Ohio, United States of America

2 Institute for Genomic Medicine, Nationwide Children's Hospital, Columbus, Ohio, United States of America

3 Dept of Mathematics and Statistics, American University, Washington D. C., United States of America

4 Dept of Statistics, The Ohio State University, Columbus, Ohio, United States of America

5 Dept of Evolution, Ecology, and Organismal Biology, The Ohio State University, Columbus, Ohio, United States of America

* lkubatko@stat.osu.edu

A Computation time and scalability

We compared the speed of each method by recording the running times for the simulation data. The required computation times for the simulated data of Scenario 9 are shown in Figure N(a) in S1 text. The time required for MO ranges from under 10 seconds (for trees with 10 tips) to approximately 30 seconds (for trees with 50 tips when ternary data are used and 80 mutations are considered). The computational speed for Scenarios 1 to 8 is similar to Scenario 9. When the number of cells is large, SiFit and SPhyR are faster (Figure N(b) in S1 text), which is not surprising as the maximum likelihood and Dollo parsimony scores are substantially cheaper to compute than the posterior probabilities. In terms of the number of mutations, the time required

for MO increases linearly with the number of mutations when the size of the tree is fixed. All jobs were run on the same node of the OSU College of Arts and Sciences Unity Cluster with 200 Gb of RAM.

B Modeling the mutation process with the multinomial distribution

In addition to the model for the mutation process described in Section 4, other models can be used for the mutation process as well. For example, we could consider the marginal probability of mutation i occurring on each branch as being proportional to the branch length. The probabilities associated with mutation on each branch can be modeled using the multinomial distribution, where each branch corresponds to a category and the probabilities are proportional to the branch length. The results using this model are quite similar to the results with the model introduced in Section 4. With the same error probability setting, the accuracy decreases as the percentage of missing values increases. When the error probability α (or β) is fixed, accuracy tends to decrease as β (or α) increase. Comparing the results of scenarios 1 and 2 in Table I in S1 text, the accuracy of MAP estimation in scenario 1 (each tree has 10 cells) tends to be slightly lower than that in scenario 2 (each tree has 50 cells) within the same setting, although the difference is small.

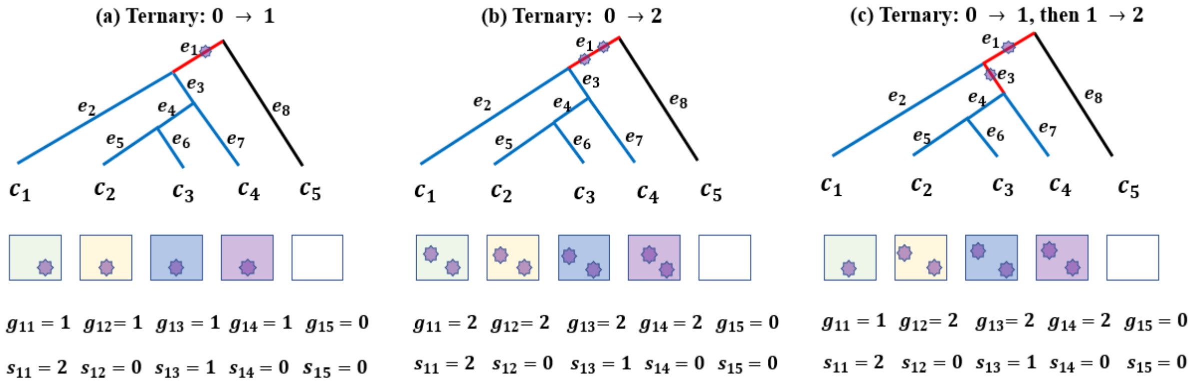

Fig A. Three possible ways that a mutation may arise on branch e_1. In (a), the status of mutation $i = 1$ transitions from $0 \to 1$ on branch e_1 and there is no further mutation at this genomic site. In (b), the status of mutation $i = 1$ transitions directly from $0 \to 2$ on branch e_1. In (c), the status of mutation $i = 1$ transitions from $0 \to 1$ on a branch e_1 and then transitions from $1 \to 2$ on branch e_3.

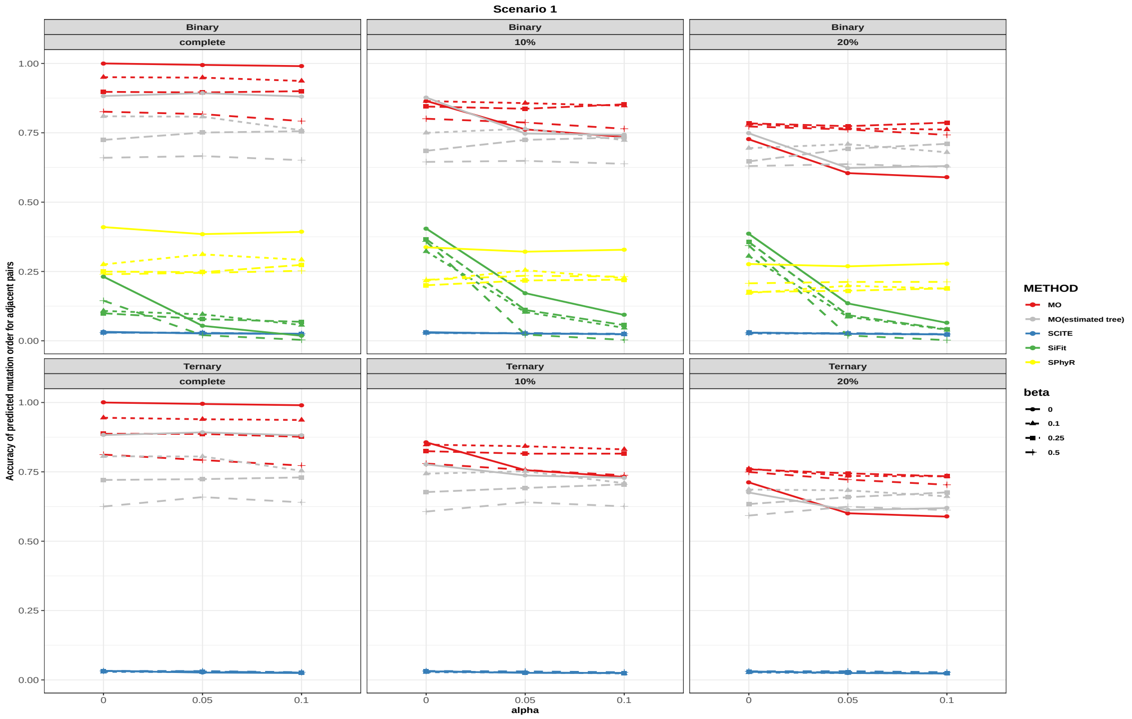

(a)

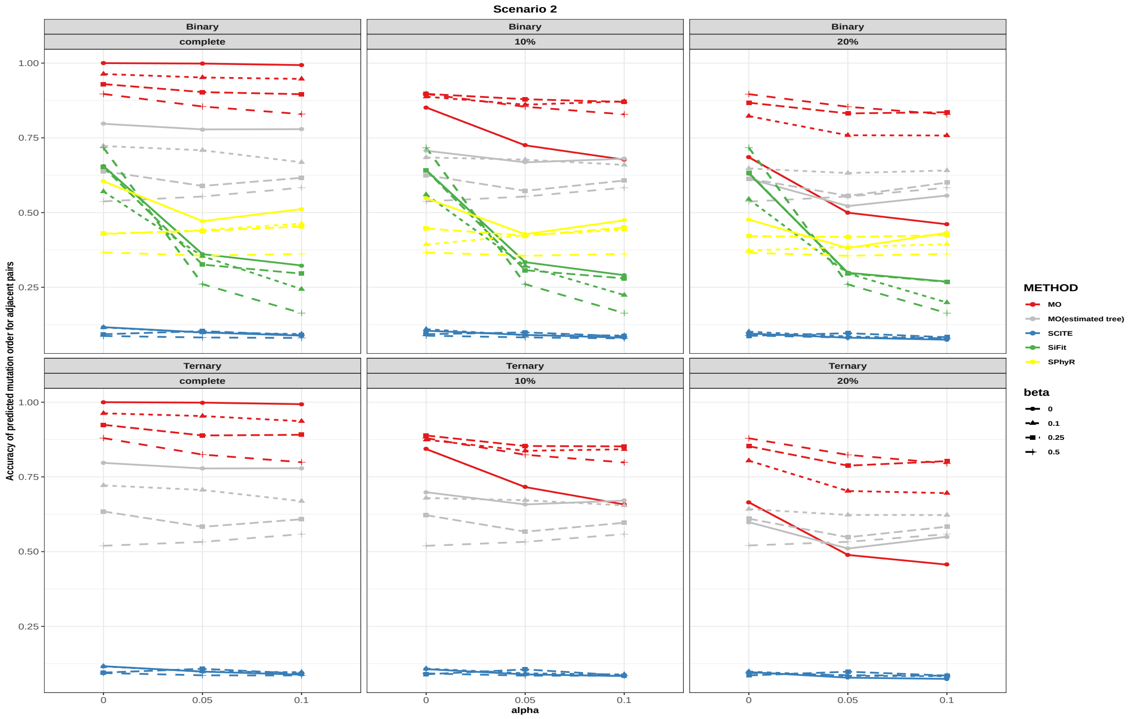

(b)

Fig B. Adjacent order accuracy in scenarios 1 and 2 of MO, SCITE, SiFit and SPhyR when there are 40 mutations. Each panel includes the results from the specific type of genotype and missing data percentage. In each panel, red, gray, blue, green and yellow colors correspond to MO with the true tree, MO with the estimated tree, SCITE, SiFit and SPhyR, respectively.

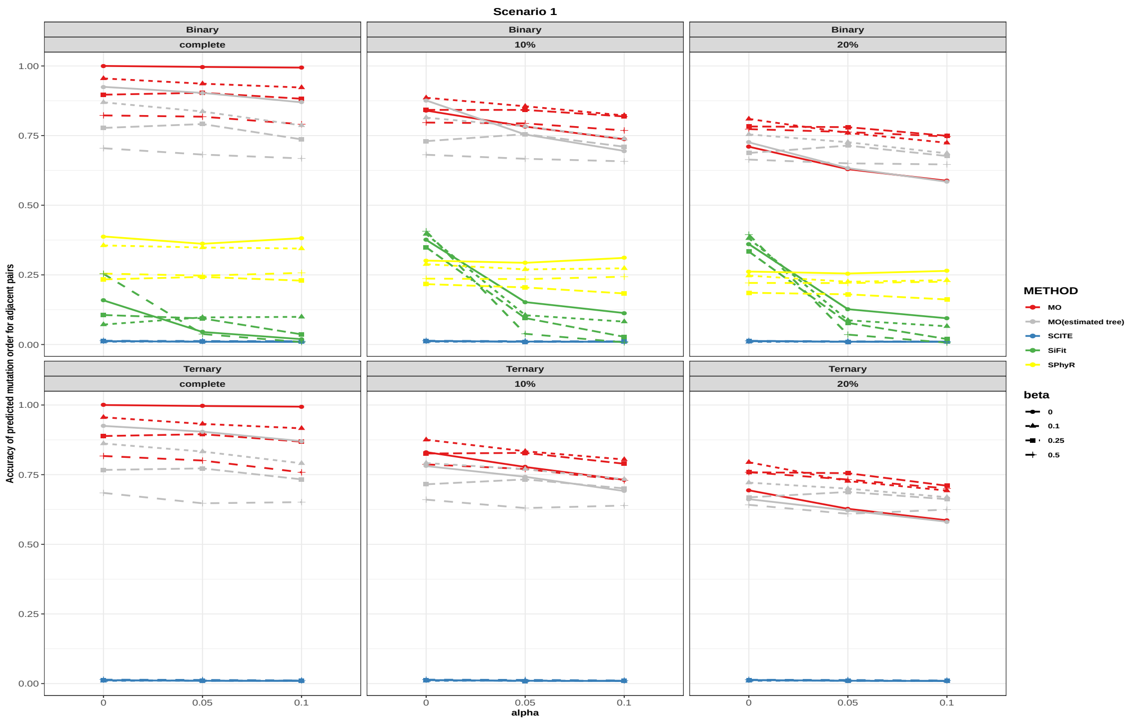

(a)

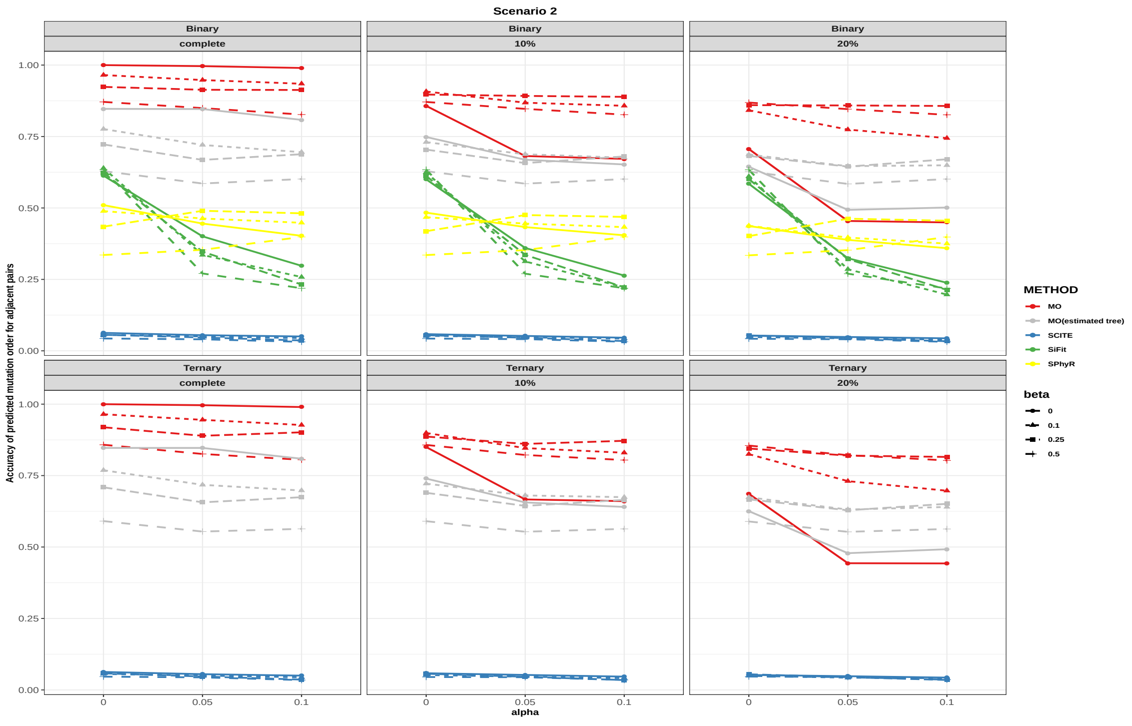

(b)

Fig C. Adjacent order accuracy in scenarios 1 and 2 of MO, SCITE, SiFit and SPhyR when there are 80 mutations. Each panel includes the results from the specific type of genotype and missing data percentage. In each panel, red, gray, blue, green and yellow colors correspond to MO with the true tree, MO with the estimated tree, SCITE, SiFit and SPhyR, respectively.

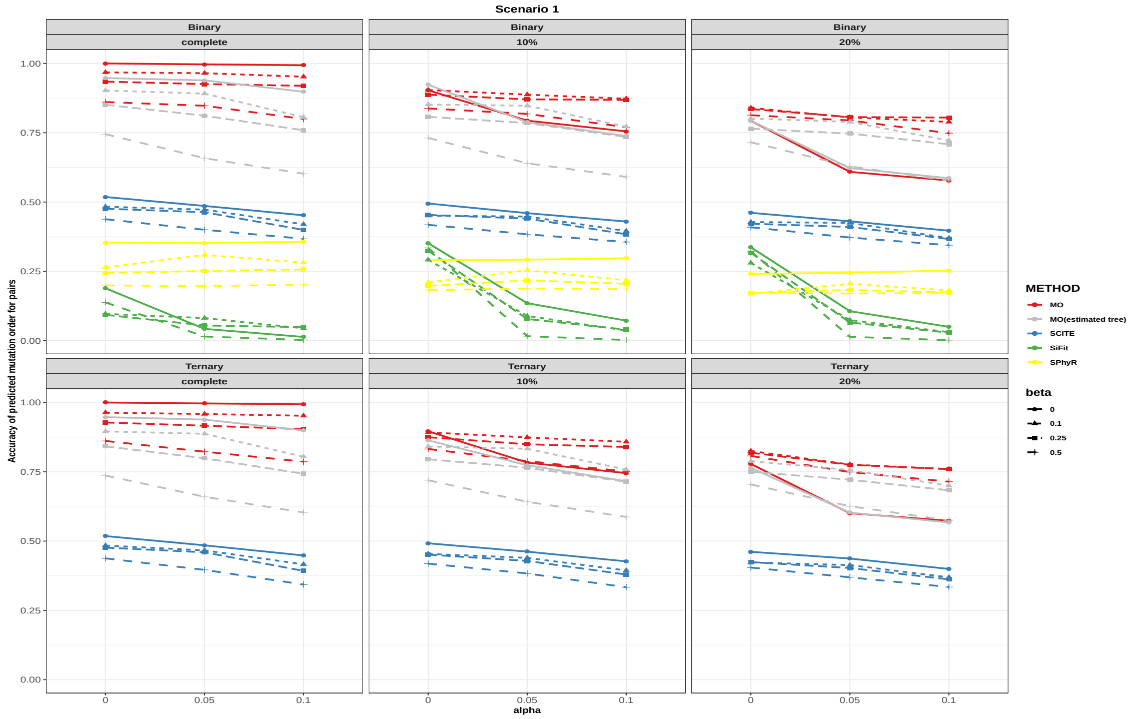

(a)

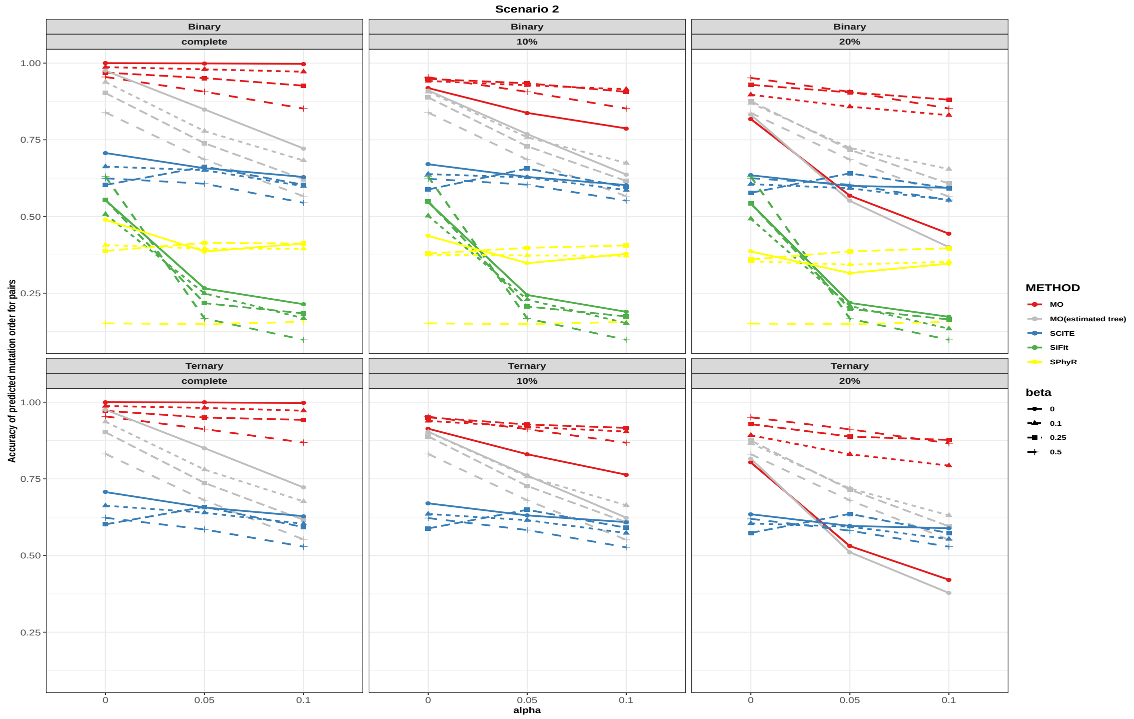

(b)

Fig D. Order accuracy in scenarios 1 and 2 of MO, SCITE, SiFit and SPhyR when there are 40 mutations. Each panel includes the results from the specific type of genotype and missing data percentage. In each panel, red, gray, blue, green and yellow colors correspond to MO with the true tree, MO with the estimated tree, SCITE, SiFit and SPhyR, respectively.

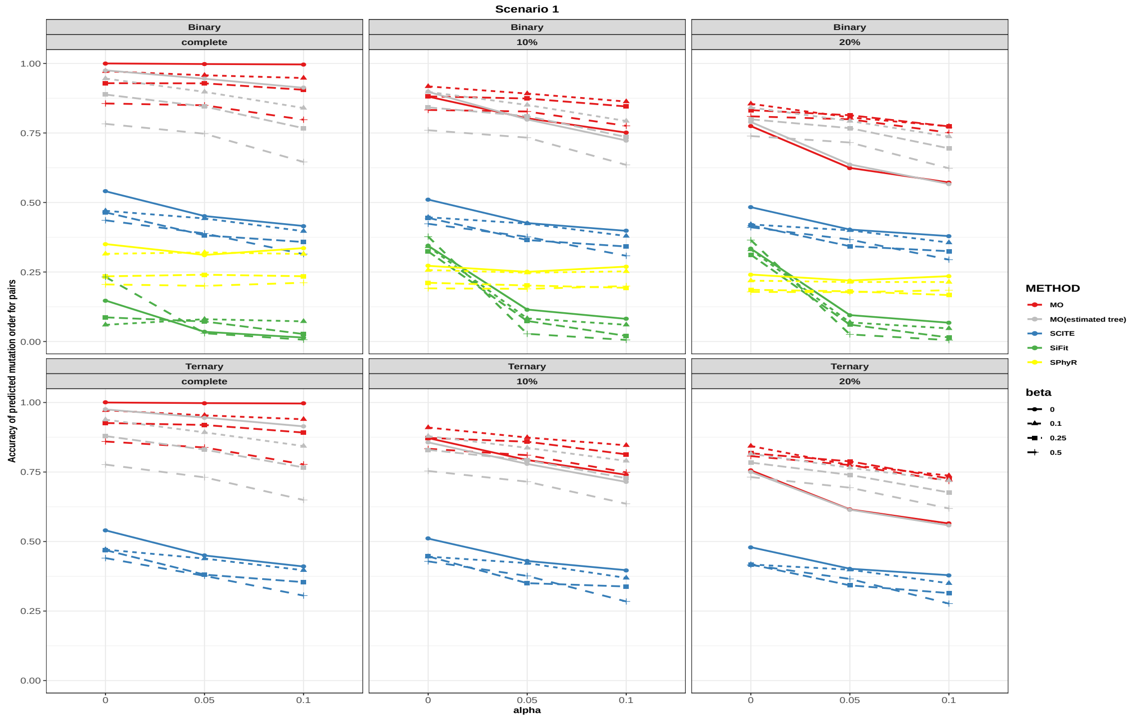

(a)

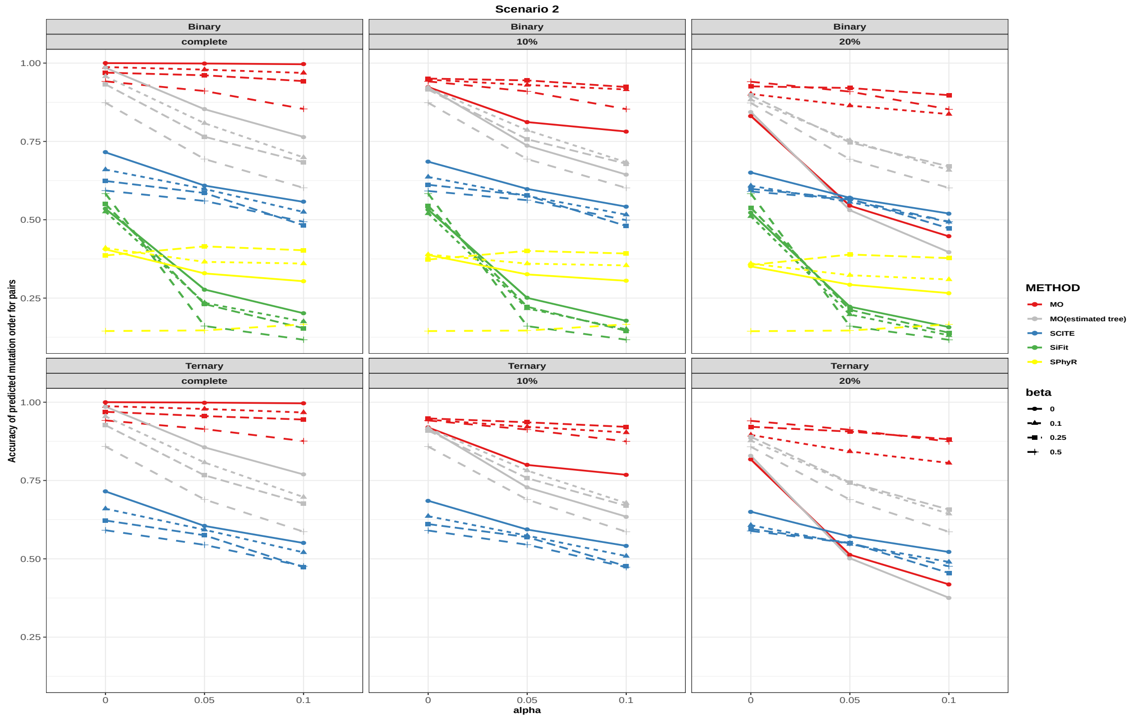

(b)

Fig E. Order accuracy in scenarios 1 and 2 of MO, SCITE, SiFit and SPhyR when there are 80 mutations. Each panel includes the results from the specific type of genotype and missing data percentage. In each panel, red, gray, blue, green and yellow colors correspond to MO with the true tree, MO with the estimated tree, SCITE, SiFit and SPhyR, respectively.

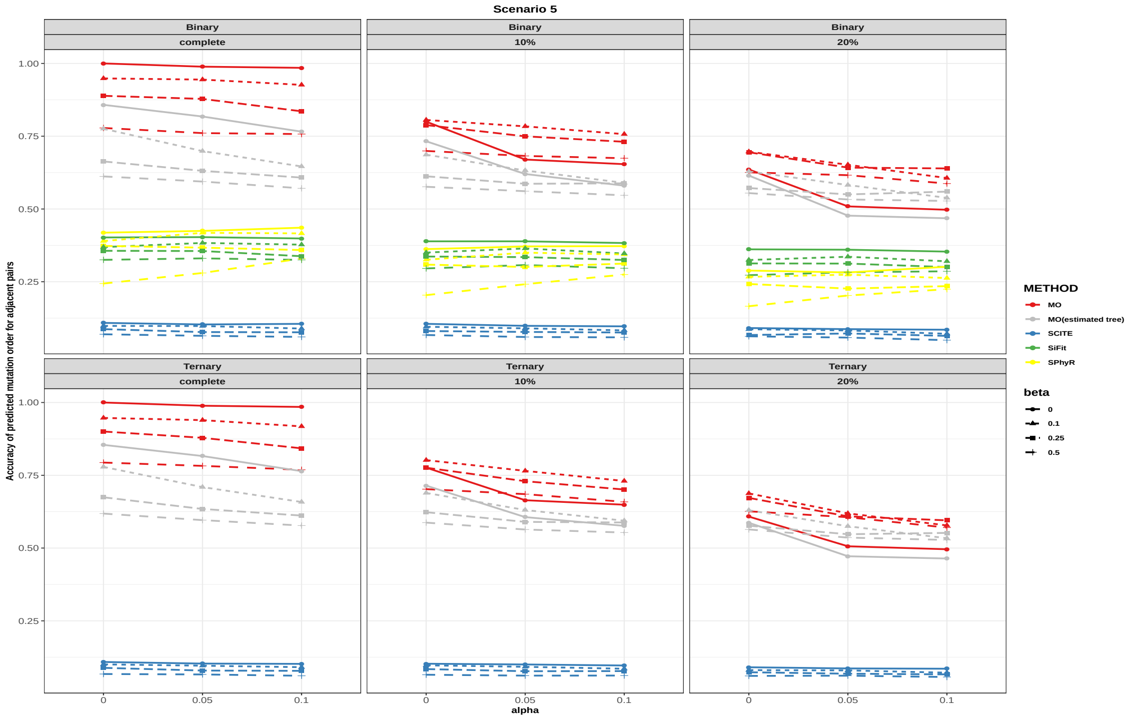

(a)

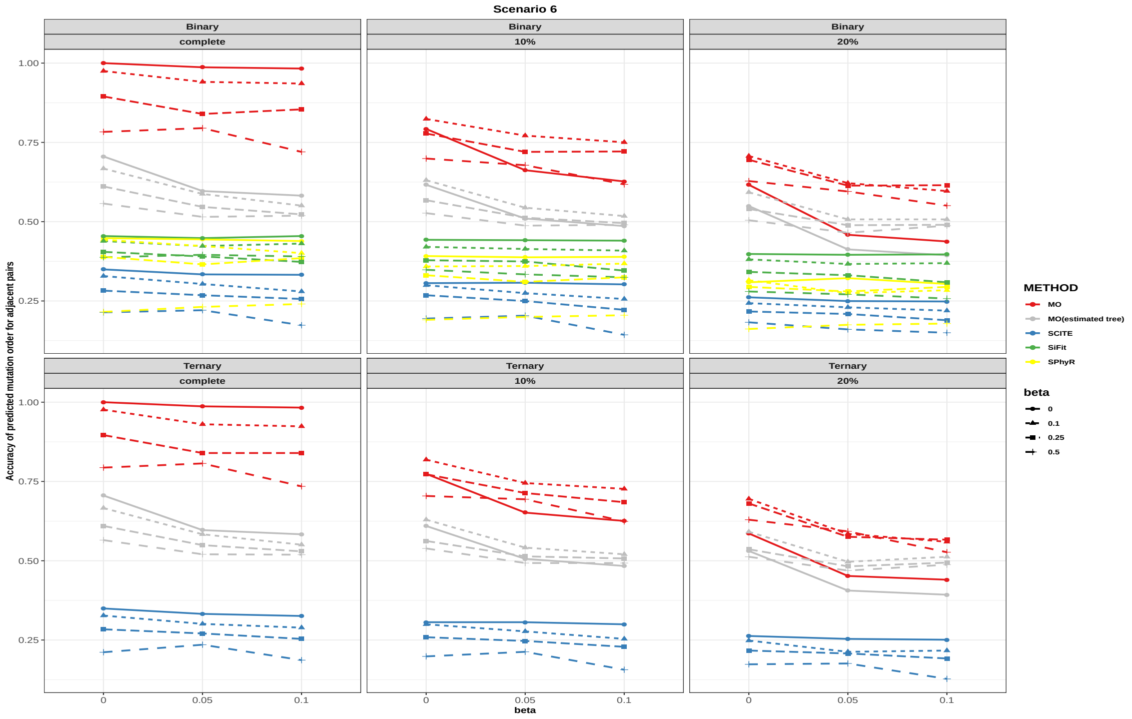

(b)

Fig F. Adjacent order accuracy in scenarios 5 and 6 of MO, SCITE, SiFit and SPhyR when there are 20 mutations. Mutations are simulated under the mutation process defined in Section 4.2. Each panel includes the results from the specific type of genotype and missing data percentage. In each panel, red, gray, blue, green and yellow colors correspond to MO with the true tree, MO with the estimated tree, SCITE, SiFit and SPhyR, respectively.

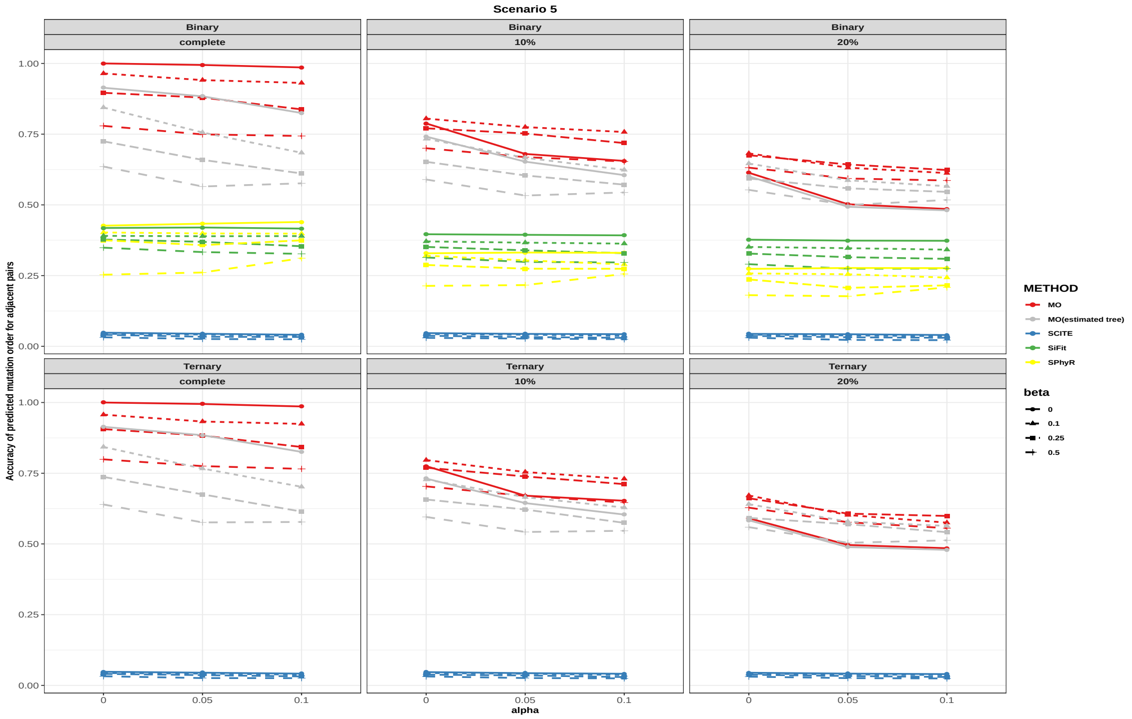

(a)

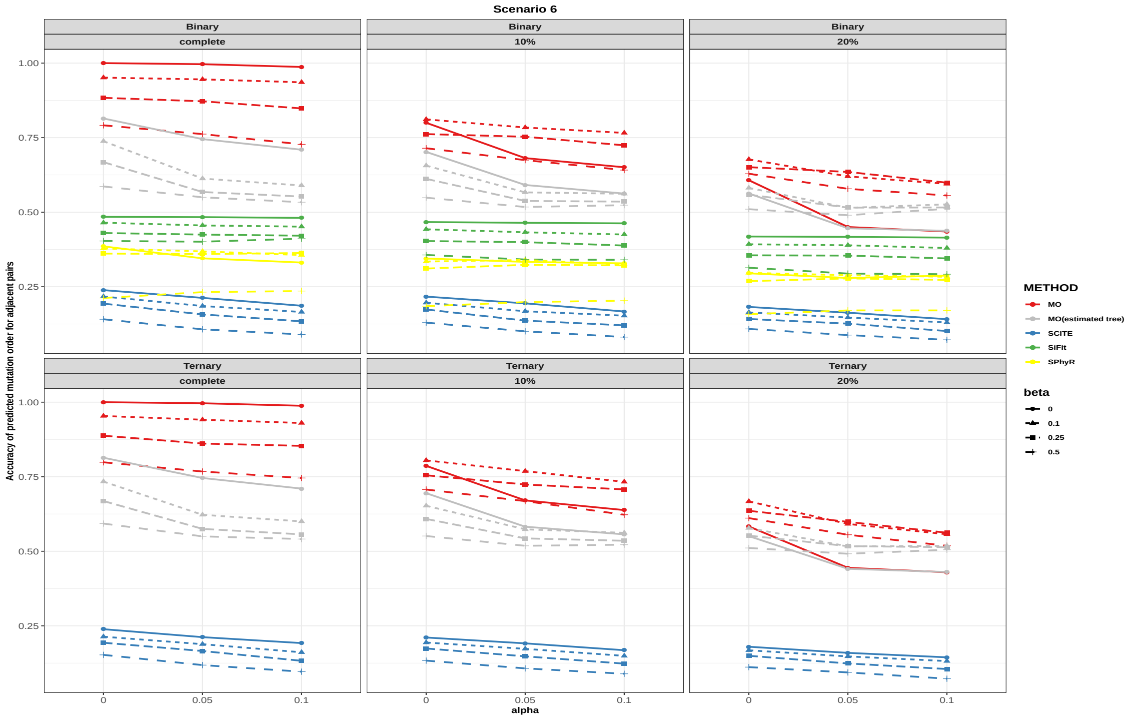

(b)

Fig G. Adjacent order accuracy in scenarios 5 and 6 of MO, SCITE, SiFit and SPhyR when there are 40 mutations. Mutations are simulated under the mutation process defined in Section 4.2. Each panel includes the results from the specific type of genotype and missing data percentage. In each panel, red, gray, blue, green and yellow colors correspond to MO with the true tree, MO with the estimated tree, SCITE, SiFit and SPhyR, respectively.

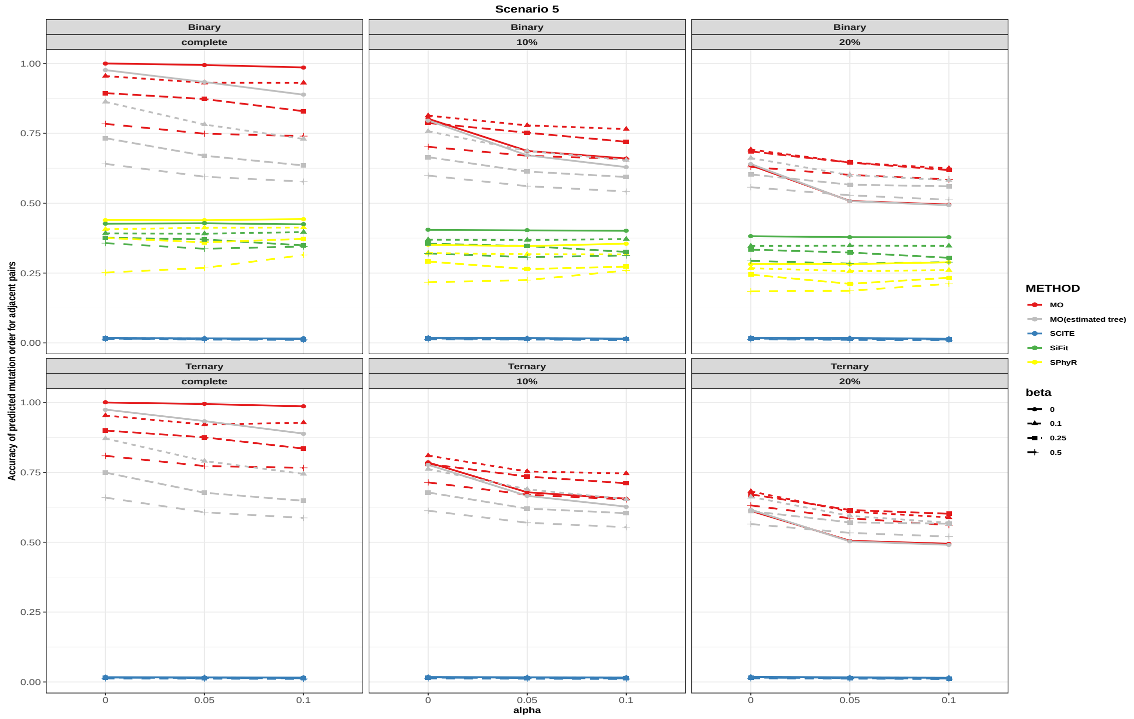

(a)

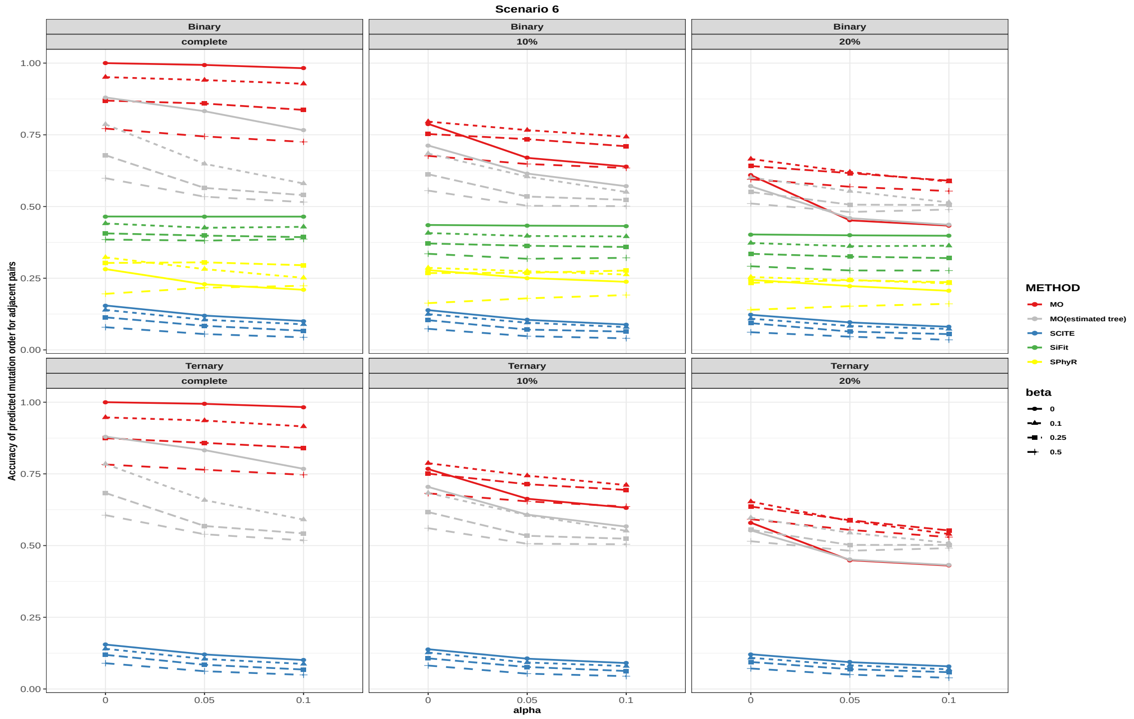

(b)

Fig H. Adjacent order accuracy in scenarios 5 and 6 of MO, SCITE, SiFit and SPhyR when there are 80 mutations. Mutations are simulated under the mutation process defined in Section 4.2. Each panel includes the results from the specific type of genotype and missing data percentage. In each panel, red, gray, blue, green and yellow colors correspond to MO with the true tree, MO with the estimated tree, SCITE, SiFit and SPhyR, respectively.

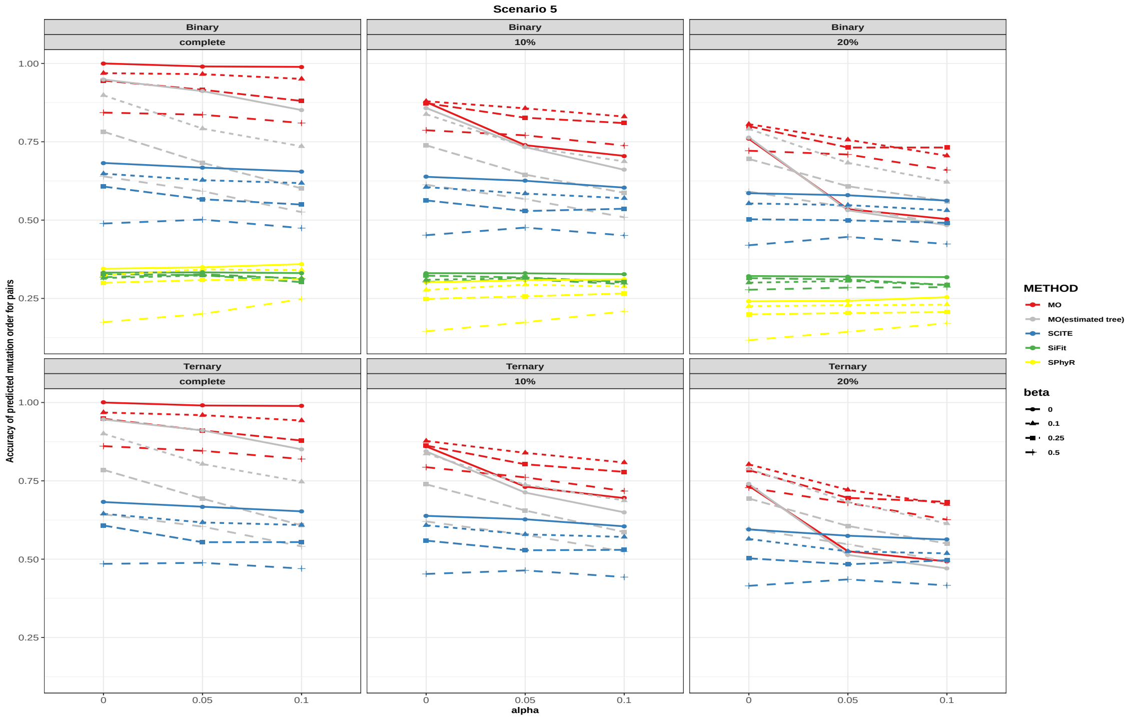

(a)

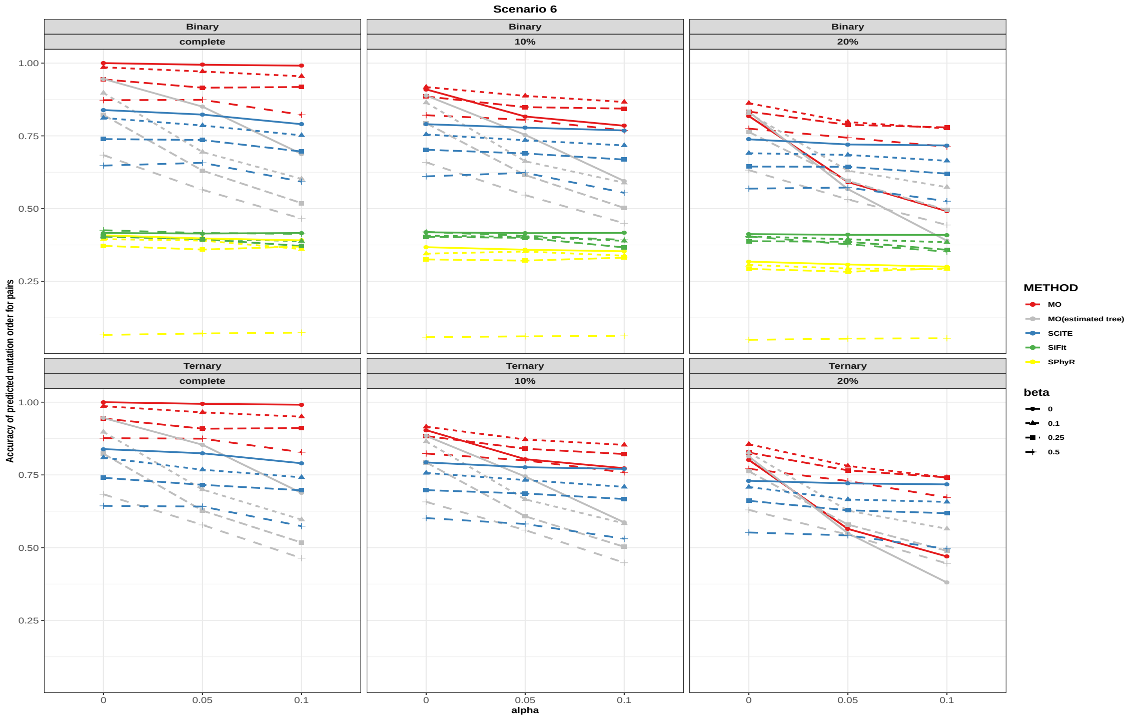

(b)

Fig I. Order accuracy in scenarios 5 and 6 of MO, SCITE, SiFit and SPhyR when there are 20 mutations. Mutations are simulated under the mutation process defined in Section 4.2. Each panel includes the results from the specific type of genotype and missing data percentage. In each panel, red, gray, blue, green and yellow colors correspond to MO with the true tree, MO with the estimated tree, SCITE, SiFit and SPhyR, respectively.

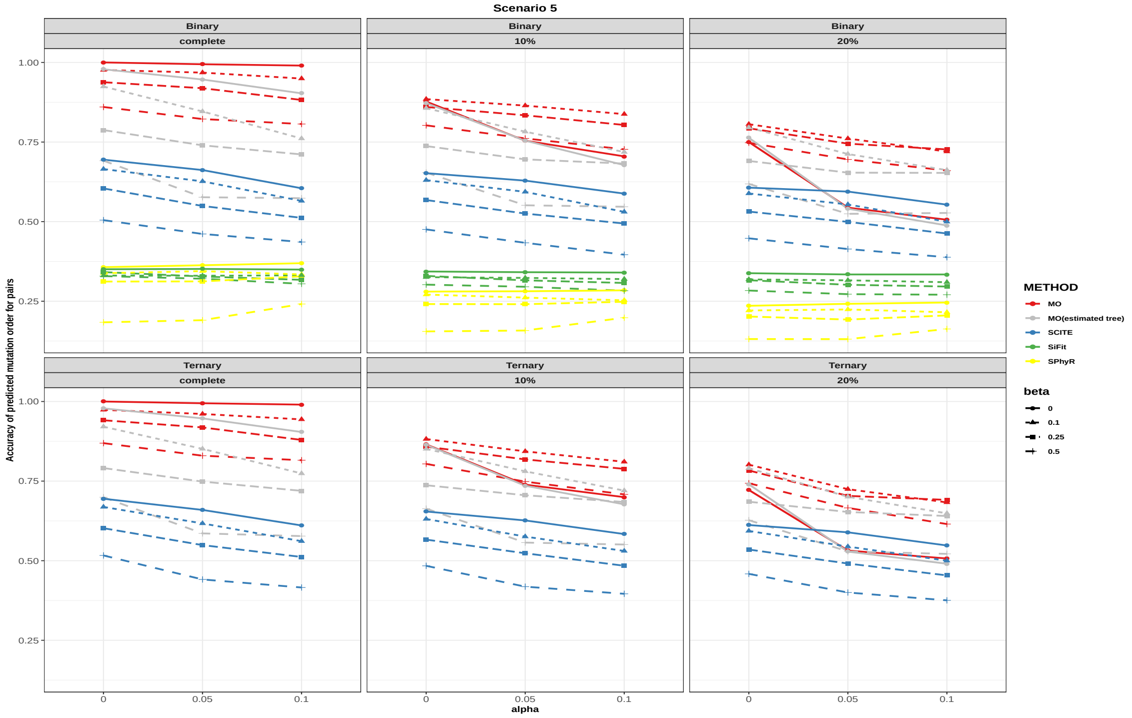

(a)

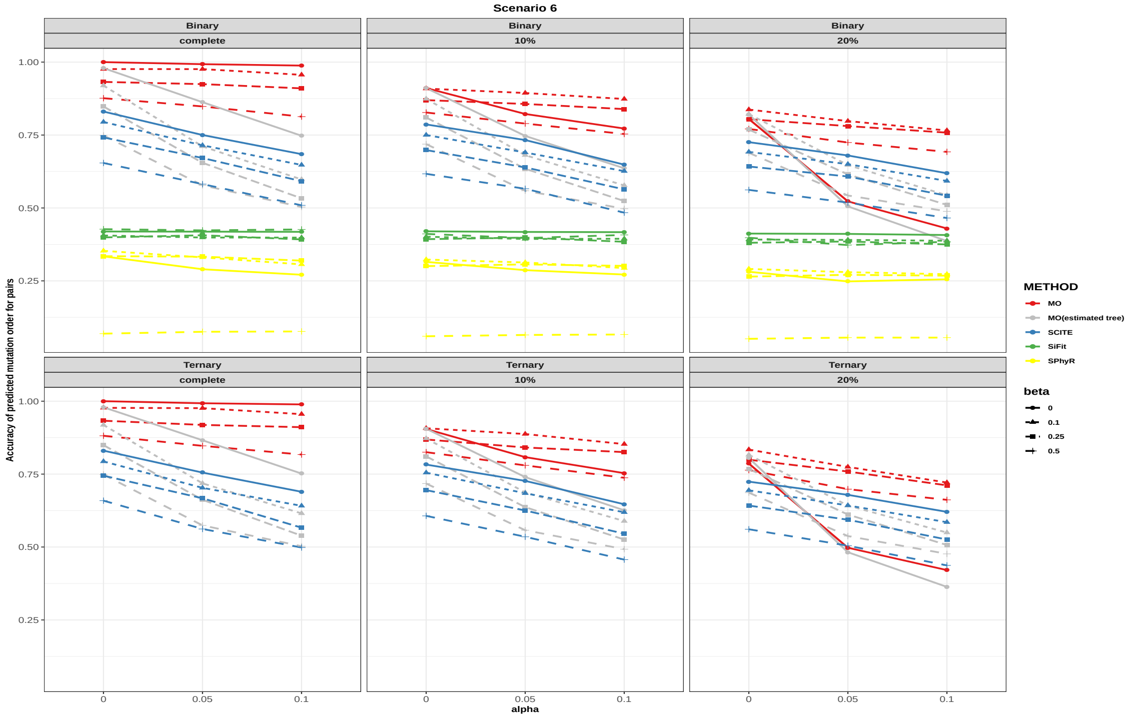

(b)

Fig J. Order accuracy in scenarios 5 and 6 of MO, SCITE, SiFit and SPhyR when there are 40 mutations. Mutations are simulated under the mutation process defined in Section 4.2. Each panel includes the results from the specific type of genotype and missing data percentage. In each panel, red, gray, blue, green and yellow colors correspond to MO with the true tree, MO with the estimated tree, SCITE, SiFit and SPhyR, respectively.

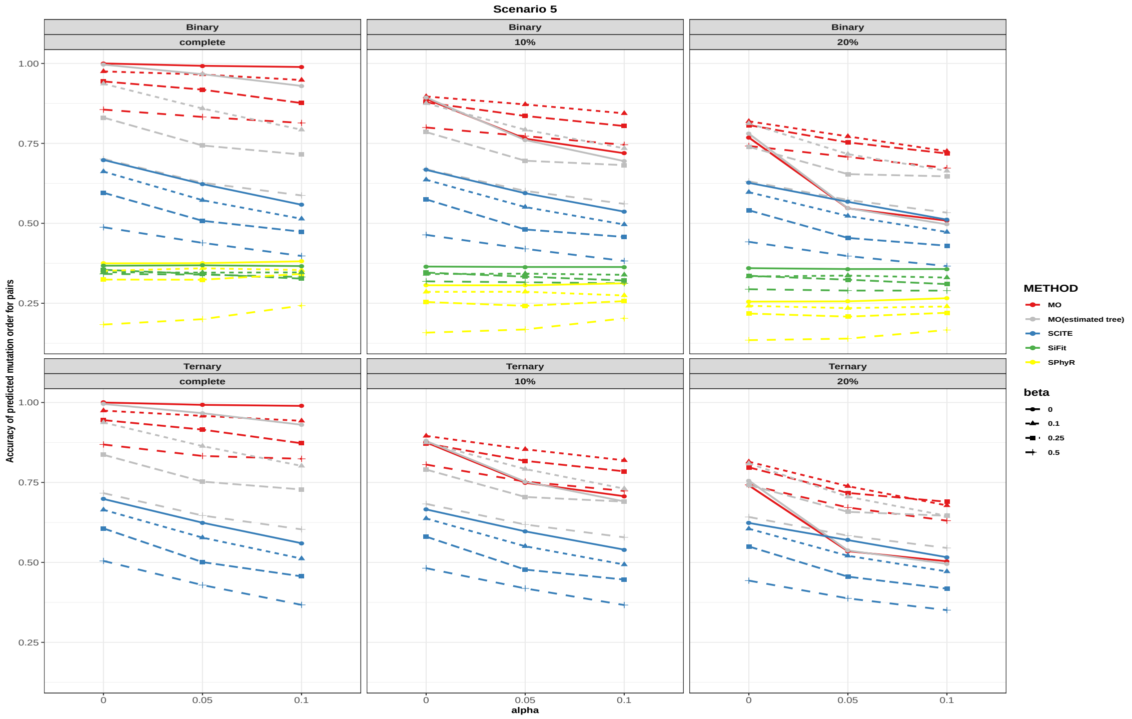

(a)

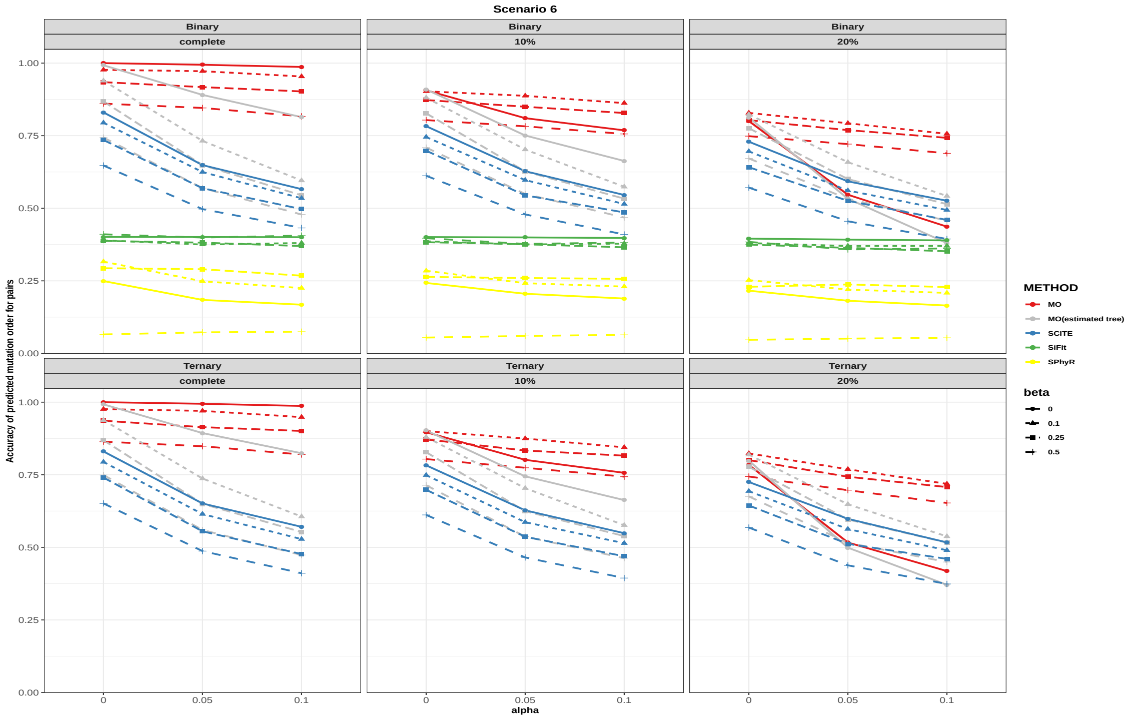

(b)

Fig K. Order accuracy in scenarios 5 and 6 of MO, SCITE, SiFit and SPhyR when there are 80 mutations. Mutations are simulated under the mutation process defined in Section 4.2. Each panel includes the results from the specific type of genotype and missing data percentage. In each panel, red, gray, blue, green and yellow colors correspond to MO with the true tree, MO with the estimated tree, SCITE, SiFit and SPhyR, respectively.

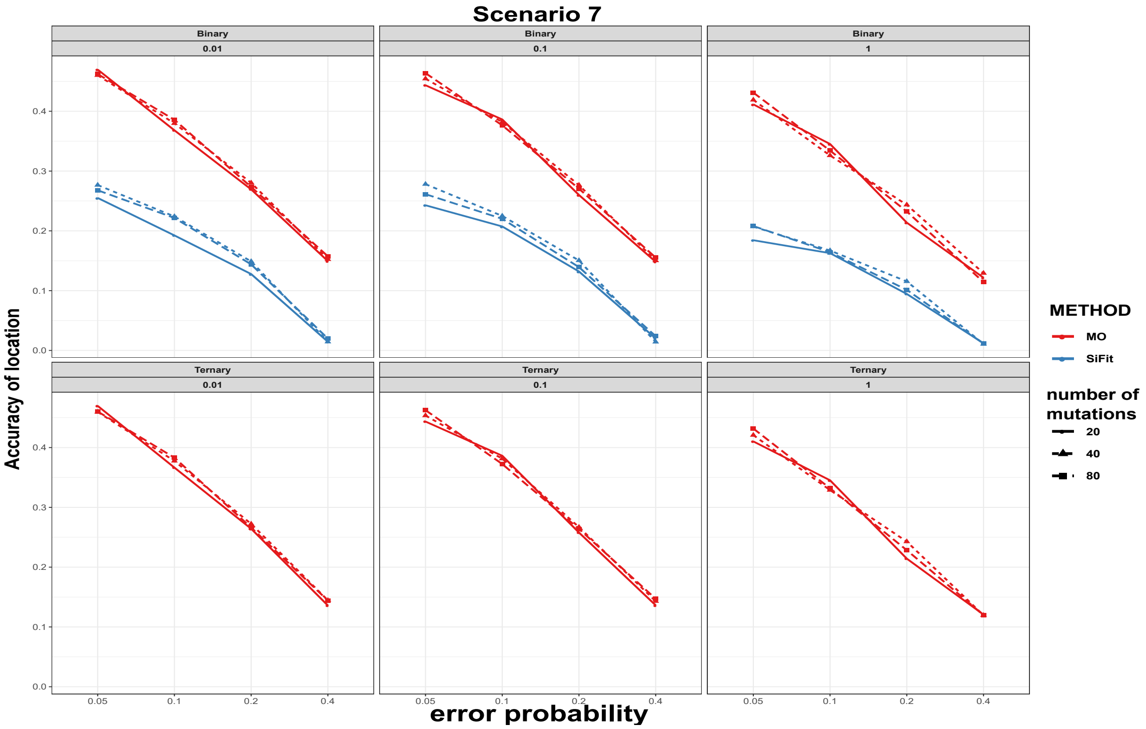

(a)

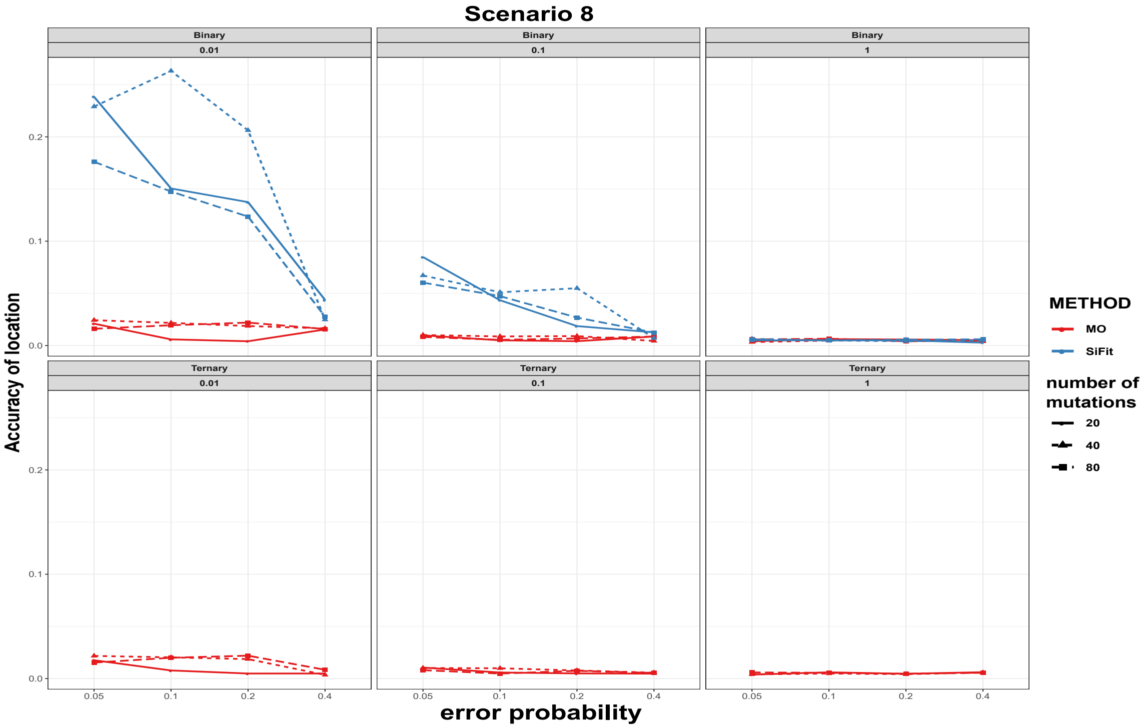

(b)

Fig L. Location accuracy in scenarios 7 and 8 of MO and SiFit. Mutations were simulated under the finite sites assumption and error rates, shown on the x-axis, are assumed to be equal.

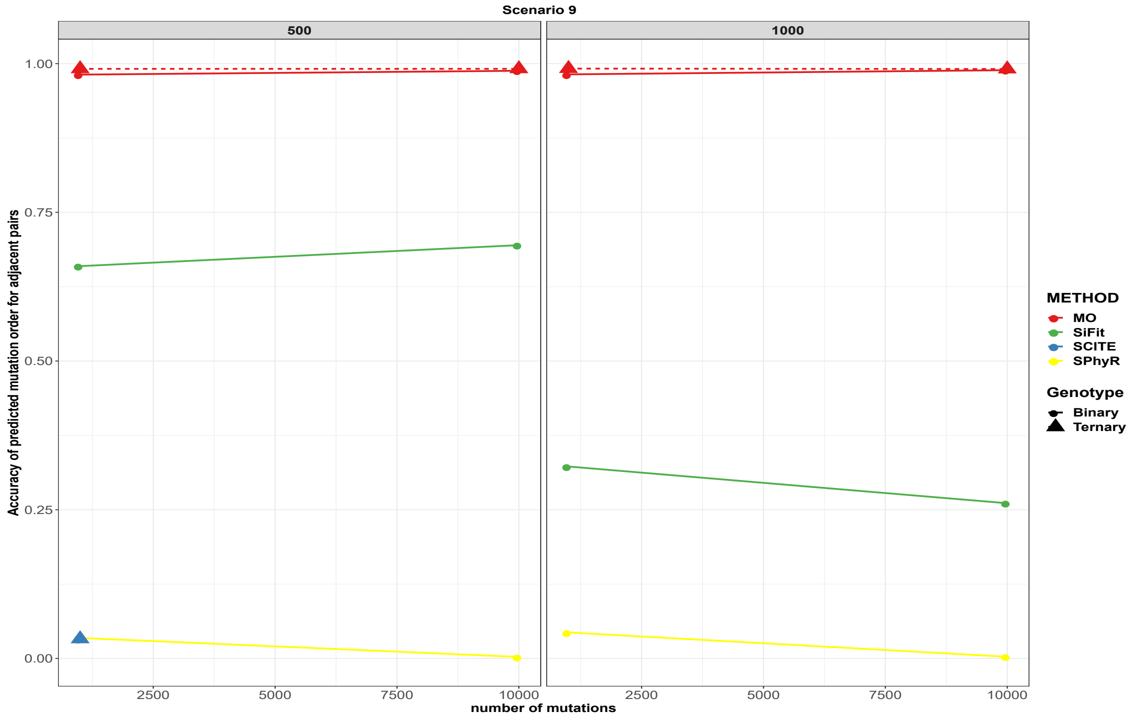

(a)

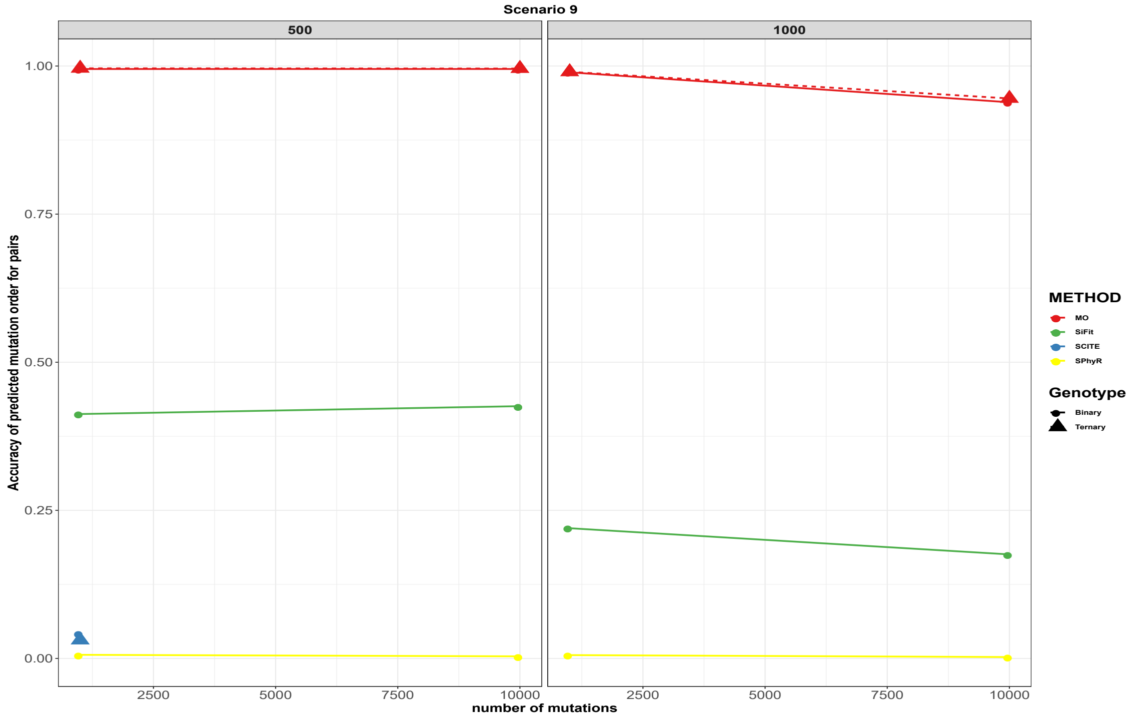

(b)

Fig M. Adjacent order accuracy (top) and order accuracy (bottom) in scenario 9 with 500 cells (left column) or 1,000 cells (right column). We did not obtain results for SCITE for the setting with 10,000 sites even after running each replicate for several days. MO has higher adjacent order accuracy and order accuracy than SiFit and SPhyR, as well as over SCITE for datasets with 1,000 sites.

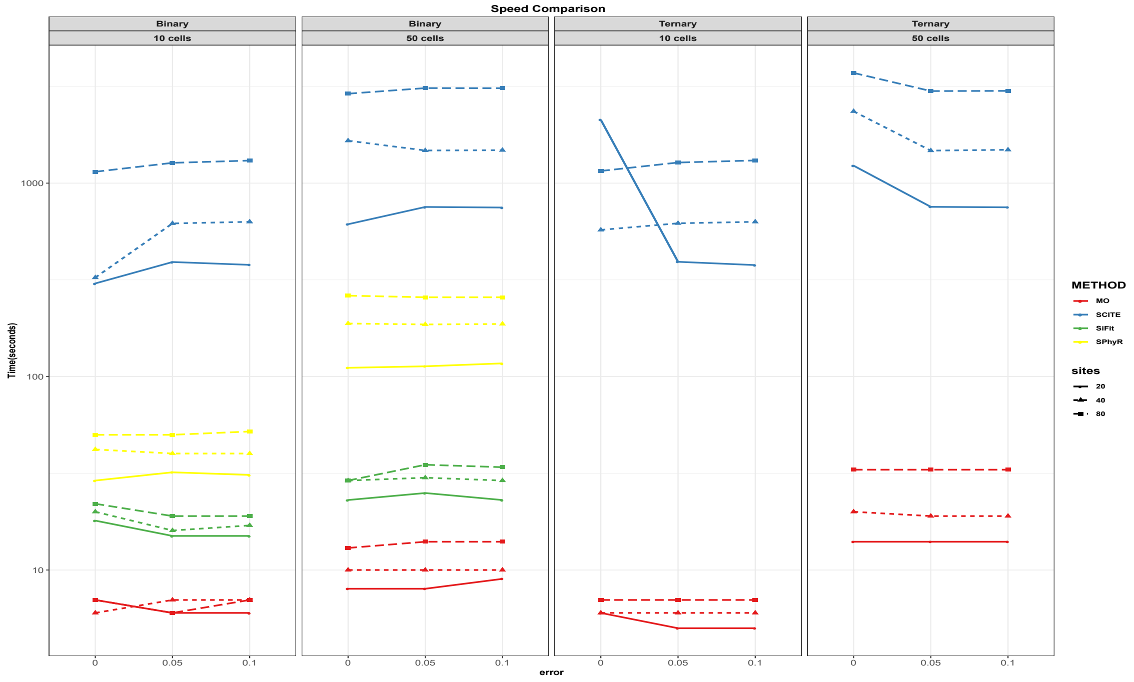

(a)

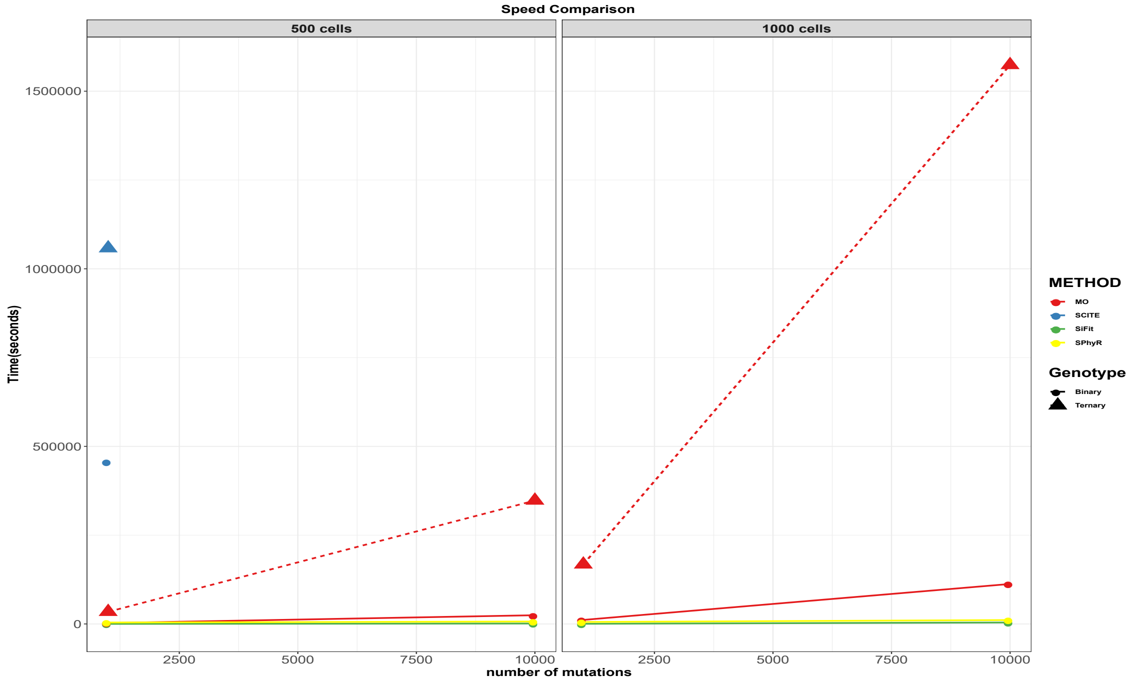

(b)

Fig N. Speed comparison The speed of the different methods for the simulation data in Scenario 9 is compared. (a) Each plotting symbol represents a different number of mutations. The x-axis is the probability of error, β. The y-axis is the computation time for 100 replicates in each setting. Note the logarithmic time scale on the y-axis. (b) Each plotting symbol represents a different genotype. The x-axis is the number of mutations in scenario 9. The y-axis is the computation time for 100 replicates in each setting. Note the logarithmic time scale on the y-axis.

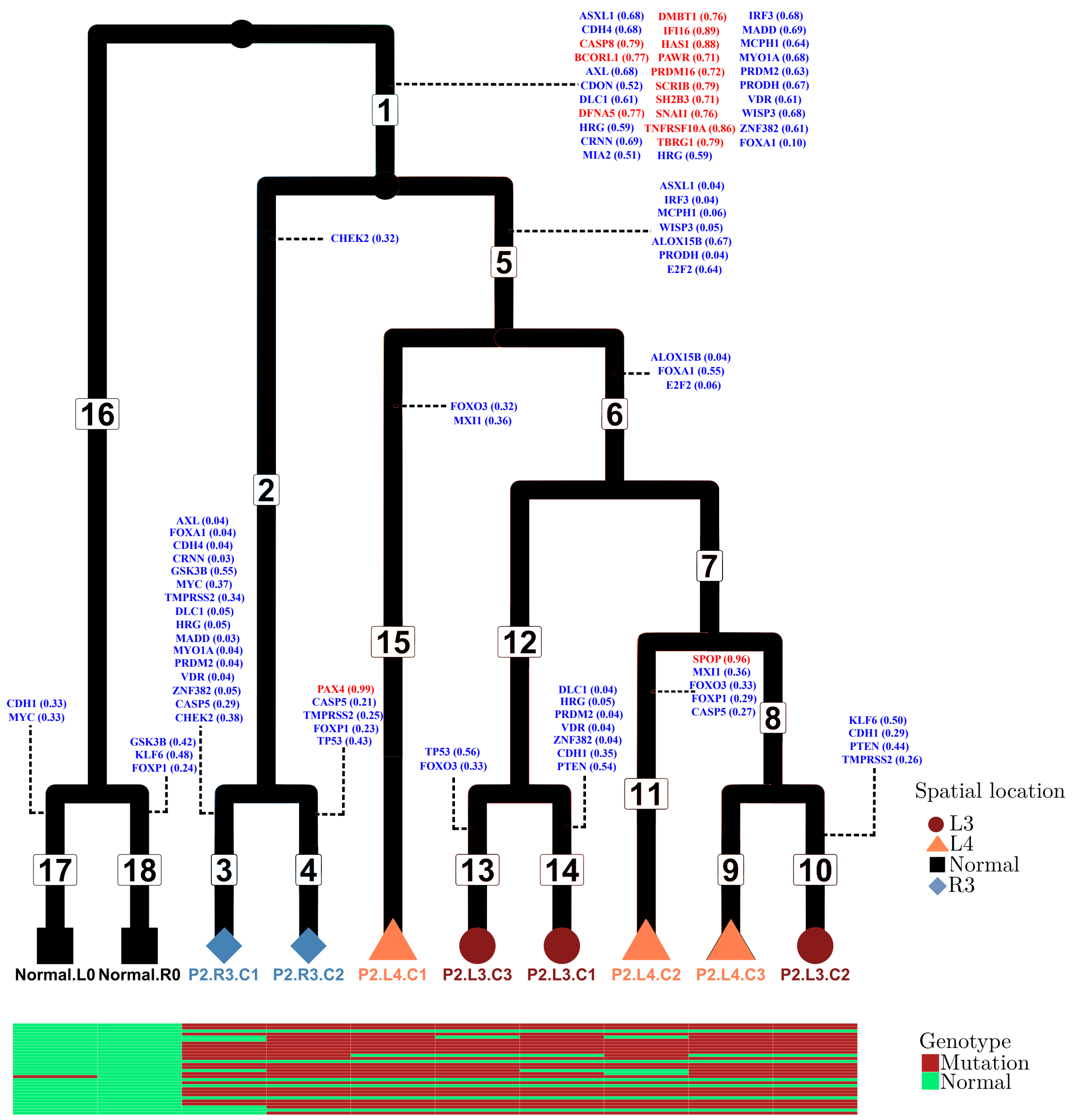

Fig O. P2 tumor phylogenetic tree and inferred temporal order of the mutations. Normal.R0 and Normal.L0 are normal cells from the right side and the left side of tissue, respectively. There are 18 branches in this tree. We do not assume the molecular clock when estimating the branch lengths, and branch lengths in this figure are not drawn to scale. The color and tip shape represent the spatial locations of the samples (normal tissue, left-side locations L3 or L4, or right-side location R3; see [1]). The temporal order of the mutations is annotated on the branches of the tree. Mutations with very strong signals (probability of occurring on one branch is greater than 0.7) are marked in red, while mutations with moderate signals (probabilities that sum to more than 0.7 on two or three branches) are marked in blue. The probability of a mutation on the indicated branch is annotated in the parentheses after each gene. Mutation data for 30 genes corresponding to the first 30 rows in Figs T and U for each tip are shown in the heatmap matrix at the bottom.

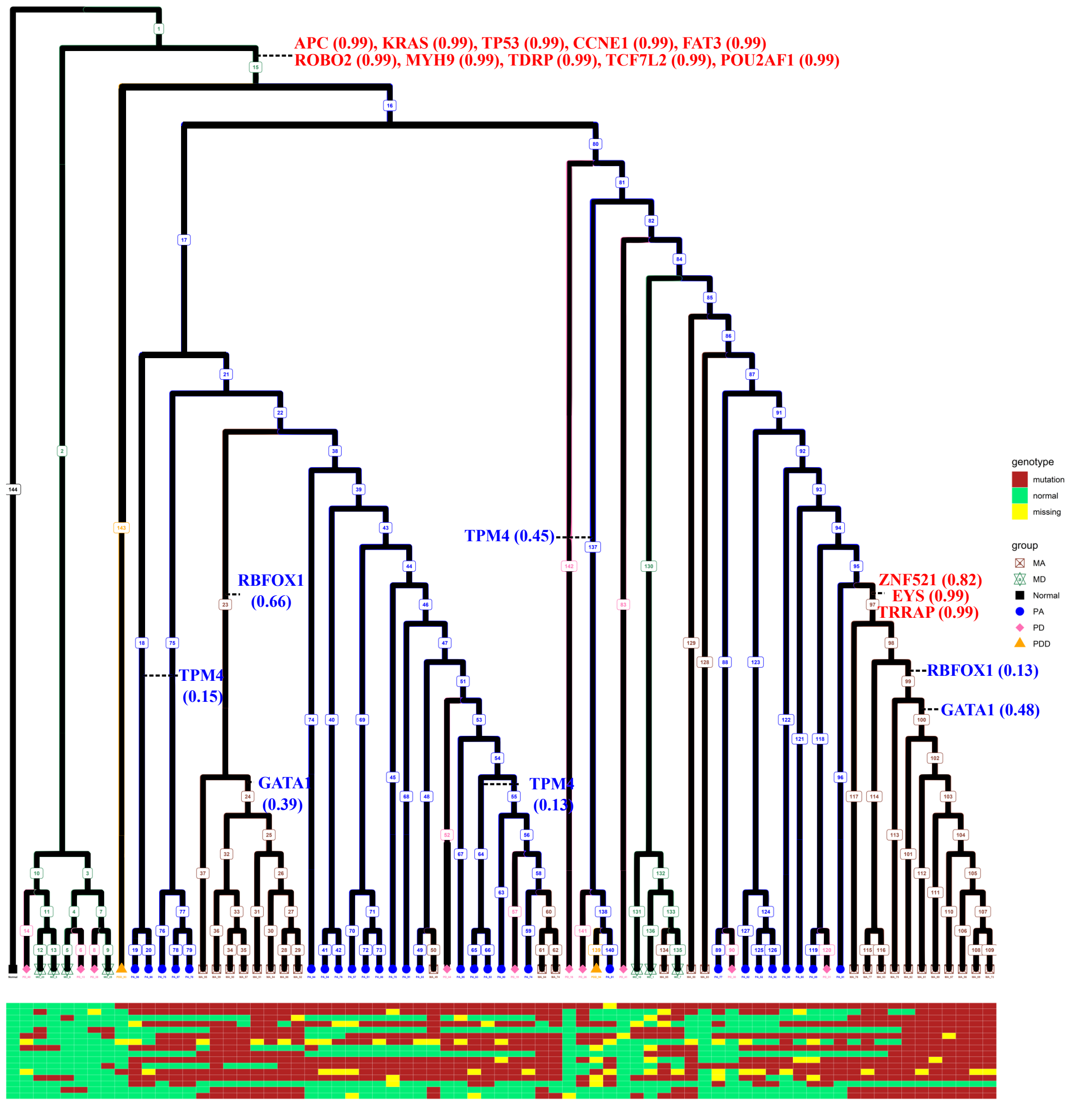

Fig P. CRC1 tumor phylogenetic tree and inferred temporal order of the mutations. The color and tip shape represent the spatial locations of the samples (Normal - normal tissue; PA - primary aneuploid; PD - primary diploid; MA - metastatic aneuploid; MD - metastatic diploid; see [2]). The temporal order of the mutations is annotated on the branches of the tree. Mutations with very strong signals (probability of occurring on one branch greater than 0.7) are marked in red, while genes with moderate signals (probabilities that sum to more than 0.7 on two or three branches) are marked in blue. The probability of a mutation on the indicated branch is annotated in the parentheses after each gene. The branch lengths are not scaled. Mutation data for the 16 genes corresponding to each tip are shown in the heatmap matrix at the bottom.

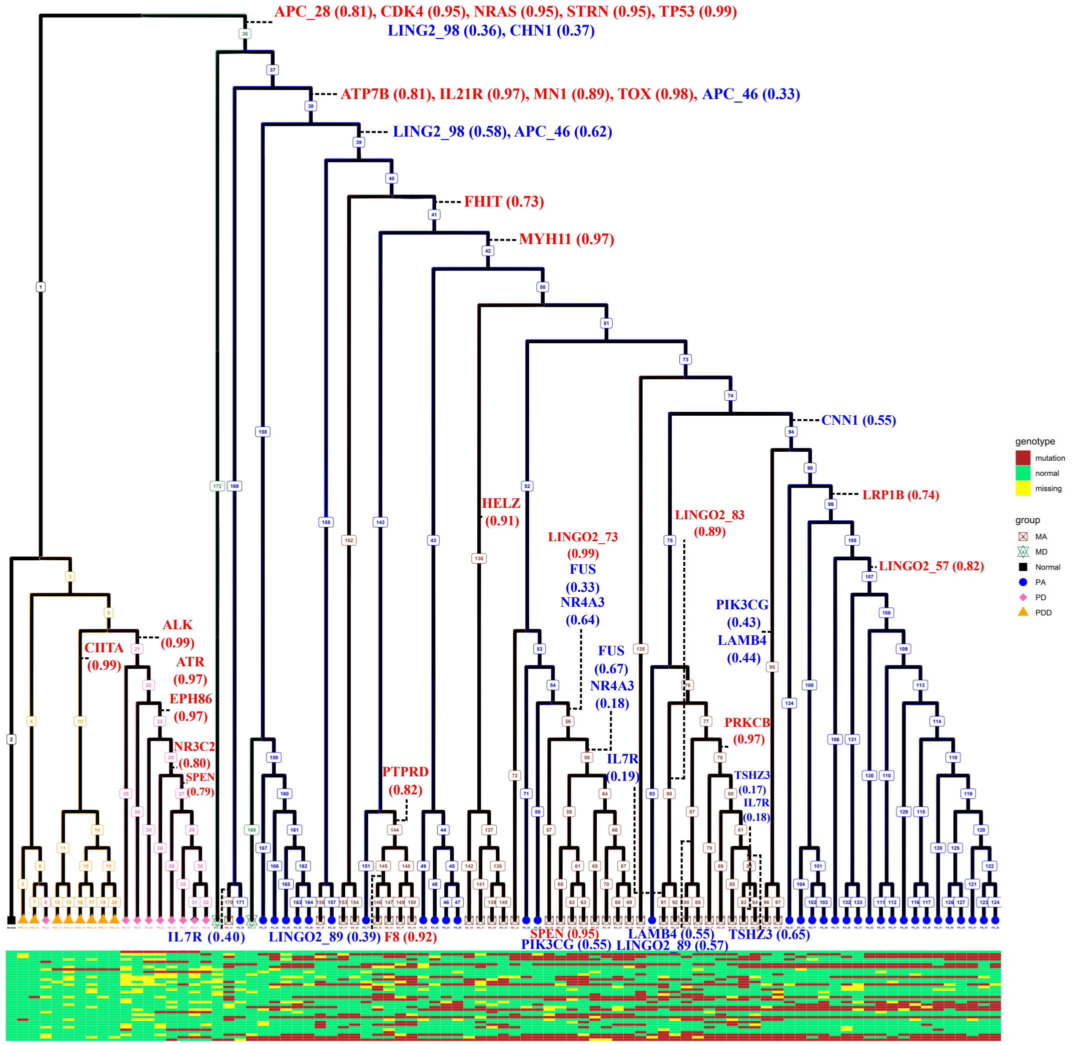

Fig Q. CRC2 tumor phylogenetic tree and inferred temporal order of the mutations. The color and tip shape represent the spatial locations of the samples (Normal - normal tissue; PA - primary aneuploid; PD - primary diploid; MA - metastatic aneuploid; MD - metastatic diploid; see [2]). The temporal order of the mutations is annotated on the branches of the tree. Mutations with very strong signals (probability of occurring on one branch greater than 0.7) are marked in red, while mutations with moderate signals (probabilities that sum to more than 0.7 on two or three branches) are marked in blue. The probability of a mutation on the indicated branch is annotated in the parentheses after each gene. The branch lengths are not scaled. Mutation data for the 36 genomic sites corresponding to each tip are shown in the heatmap matrix at the bottom.

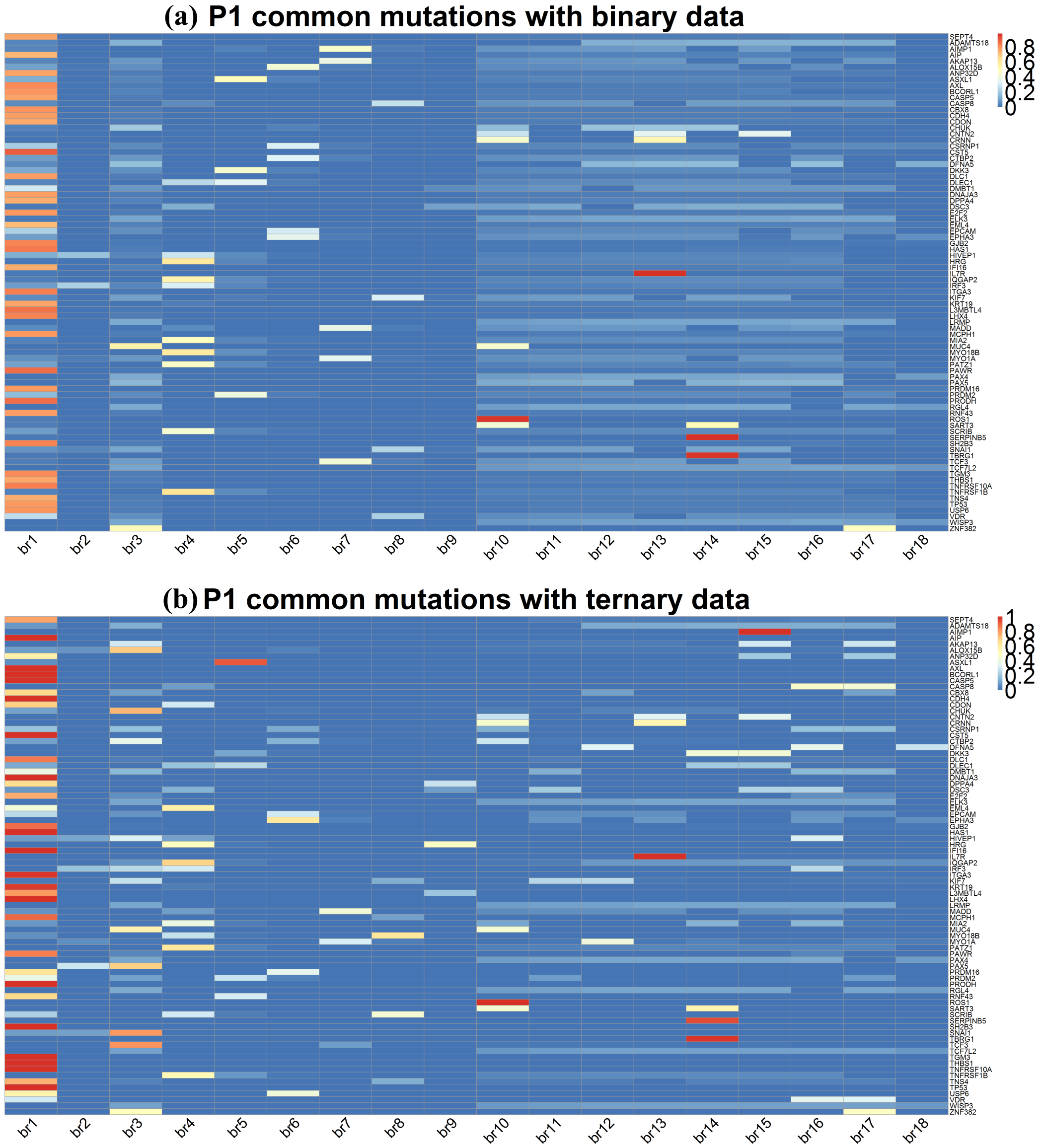

Fig R. Heatmap of posterior probabilities that each mutation occurs on each branch for common tumor suppressor genes or oncogenes for prostate cancer patient P1 with prior distributions that have larger variances. Color indicates the magnitude of the probability, with red indicating probability close to 1 and blue indicating probability close to 0. For P1, prior of α is set as $\alpha \sim Beta(0.29, 0.71)$ (larger variance). The prior of β is set as $\beta \sim Beta(0.02, 0.98)$ (larger variance). Distribution of transition rate λ_1 is set as $\lambda_1 \sim Gamma(2, 5.0 \times 10^{-8})$ (larger variance). Distribution of transition rate λ_2 is set as $\lambda_2 \sim Gamma(2, 5.0 \times 10^{-3})$ (larger variance).

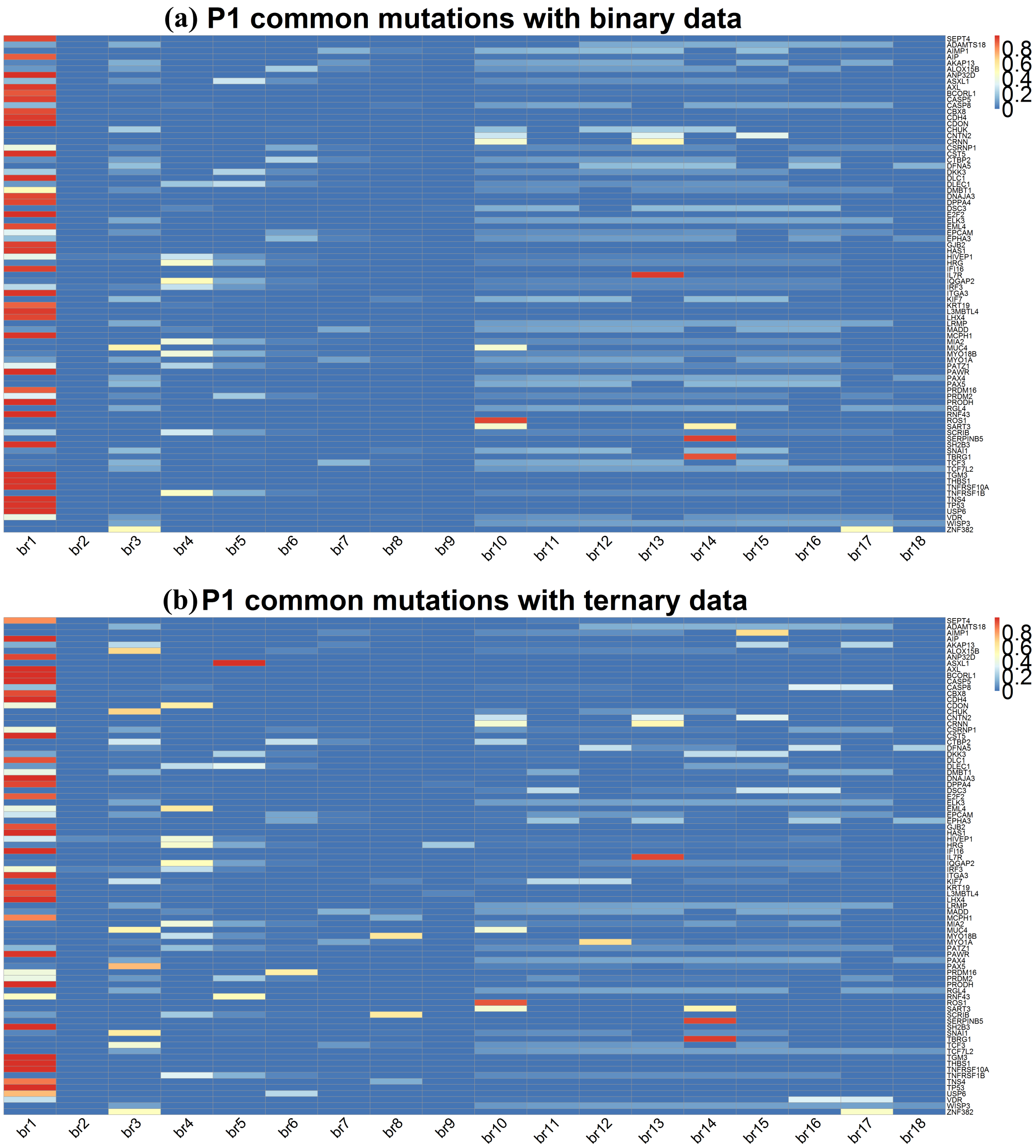

Fig S. Heatmap of posterior probabilities that each mutation occurs on each branch for common tumor suppressor genes or oncogenes for prostate cancer patient P1 with prior distributions that have smaller variances. Color indicates the magnitude of the probability, with red indicating probability close to 1 and blue indicating probability close to 0. For P1, prior of α is set as $\alpha \sim Beta(2.9, 7.1)$ (smaller variance). The prior of β is set as $\beta \sim Beta(0.2, 9.8)$ (smaller variance). Distribution of transition rate λ_1 is set as $\lambda_1 \sim Gamma(5, 2.0 \times 10^{-8})$ (smaller variance). Distribution of transition rate λ_2 is set as $\lambda_2 \sim Gamma(5, 2.0 \times 10^{-3})$ (smaller variance).

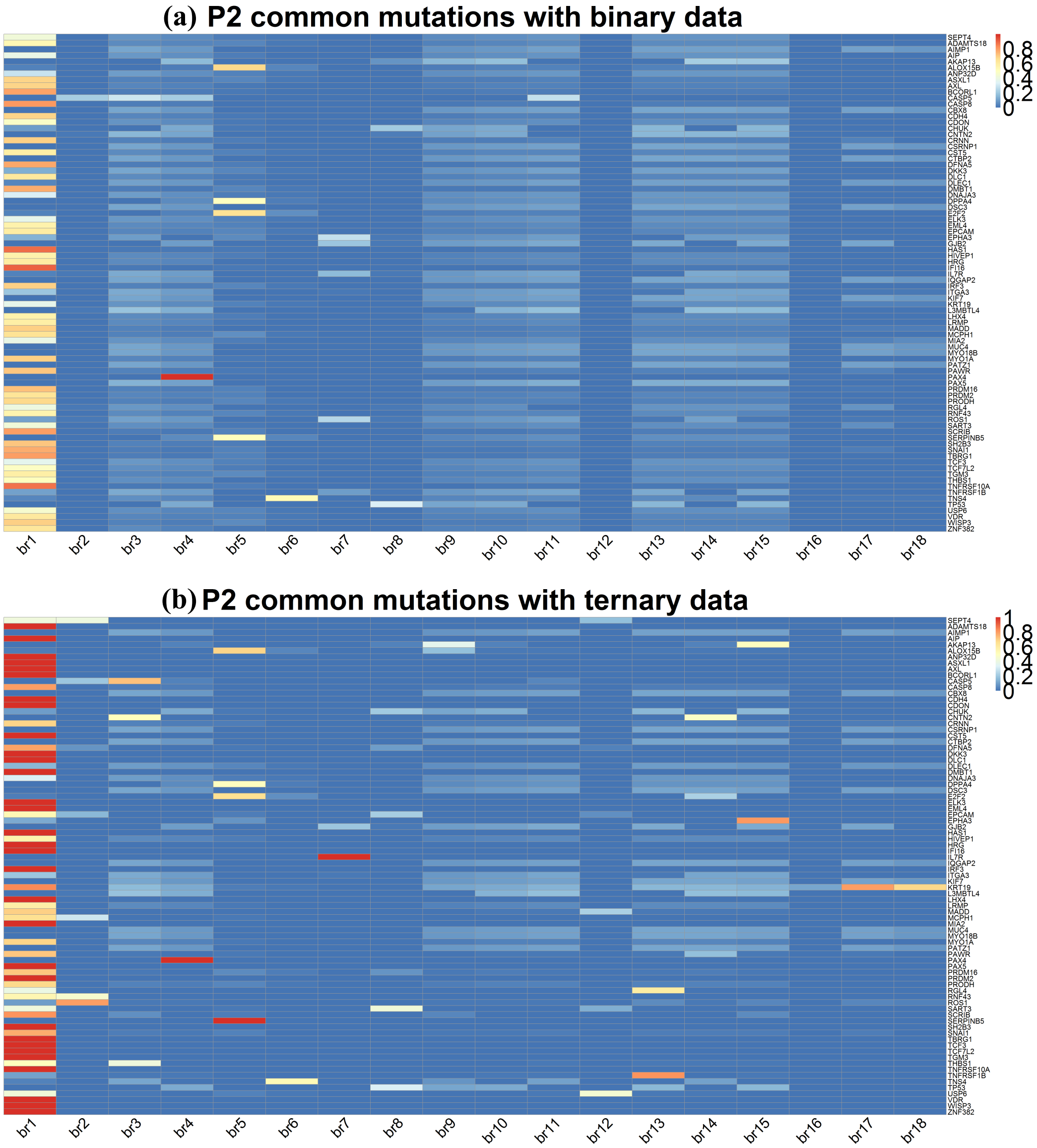

Fig T. Heatmap of posterior probabilities that each mutation occurs on each branch for common tumor suppressor genes or oncogenes for prostate cancer patient P2 with prior distributions that have larger variances. Color indicates the magnitude of the probability, with red indicating probability close to 1 and blue indicating probability close to 0. For P2, prior of α is set as $\alpha \sim Beta(0.31, 0.69)$ (larger variance). The prior of β is set as $\beta \sim Beta(0.02, 0.98)$ (larger variance). Prior distributions for the transition rate parameters are as in Fig R (larger variance).

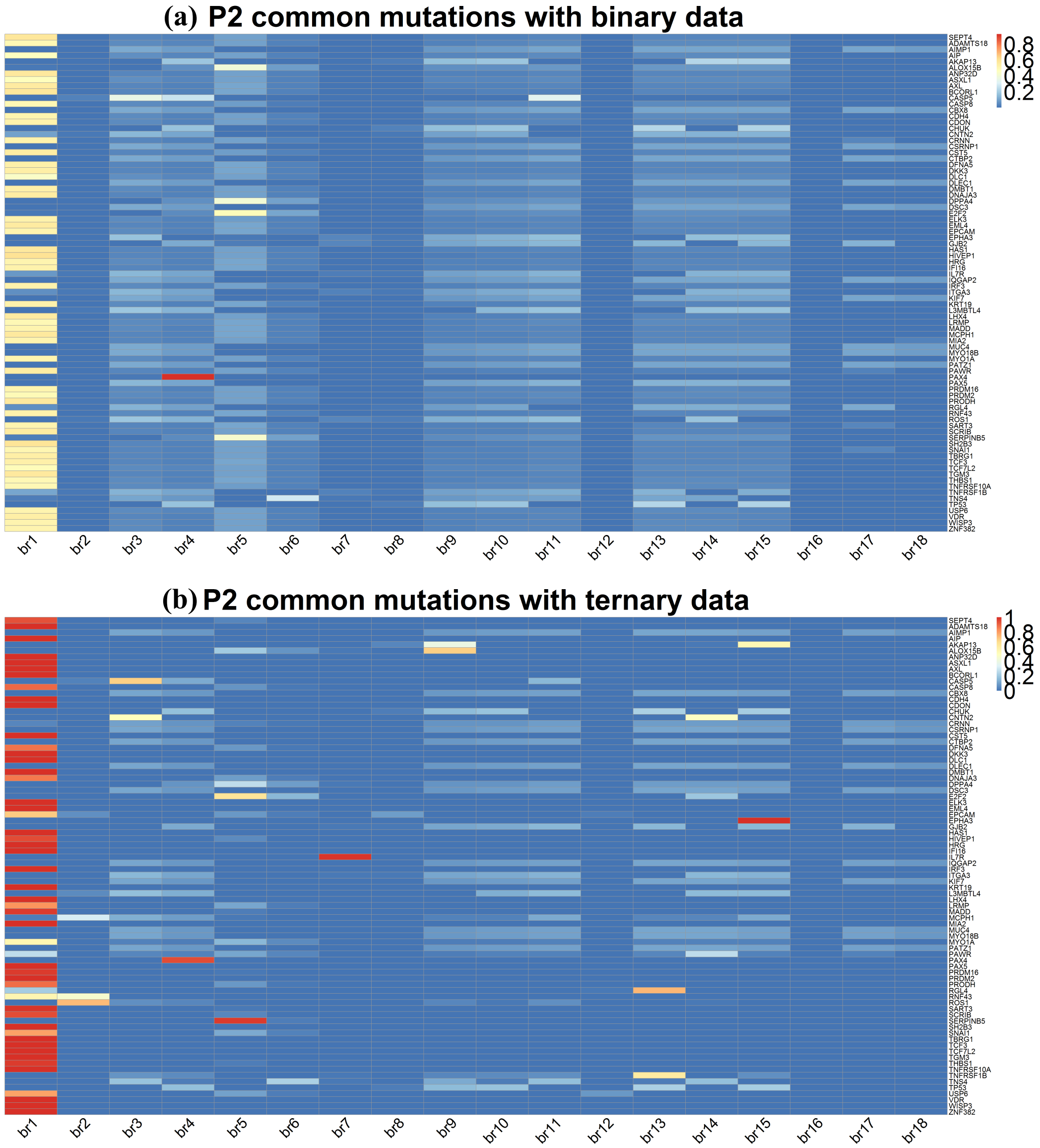

Fig U. Heatmap of posterior probabilities that each mutation occurs on each branch for common tumor suppressor genes or oncogenes for prostate cancer patient P2 with prior distributions that have smaller variances. Color indicates the magnitude of the probability, with red indicating probability close to 1 and blue indicating probability close to 0. For P2, prior of α is set as $\alpha \sim Beta(3.1, 6.9)$ (smaller variance). The prior of β is set as $\beta \sim Beta(0.2, 9.8)$ (smaller variance). Prior distributions for the transition rate parameters are as in Fig S (smaller variance).

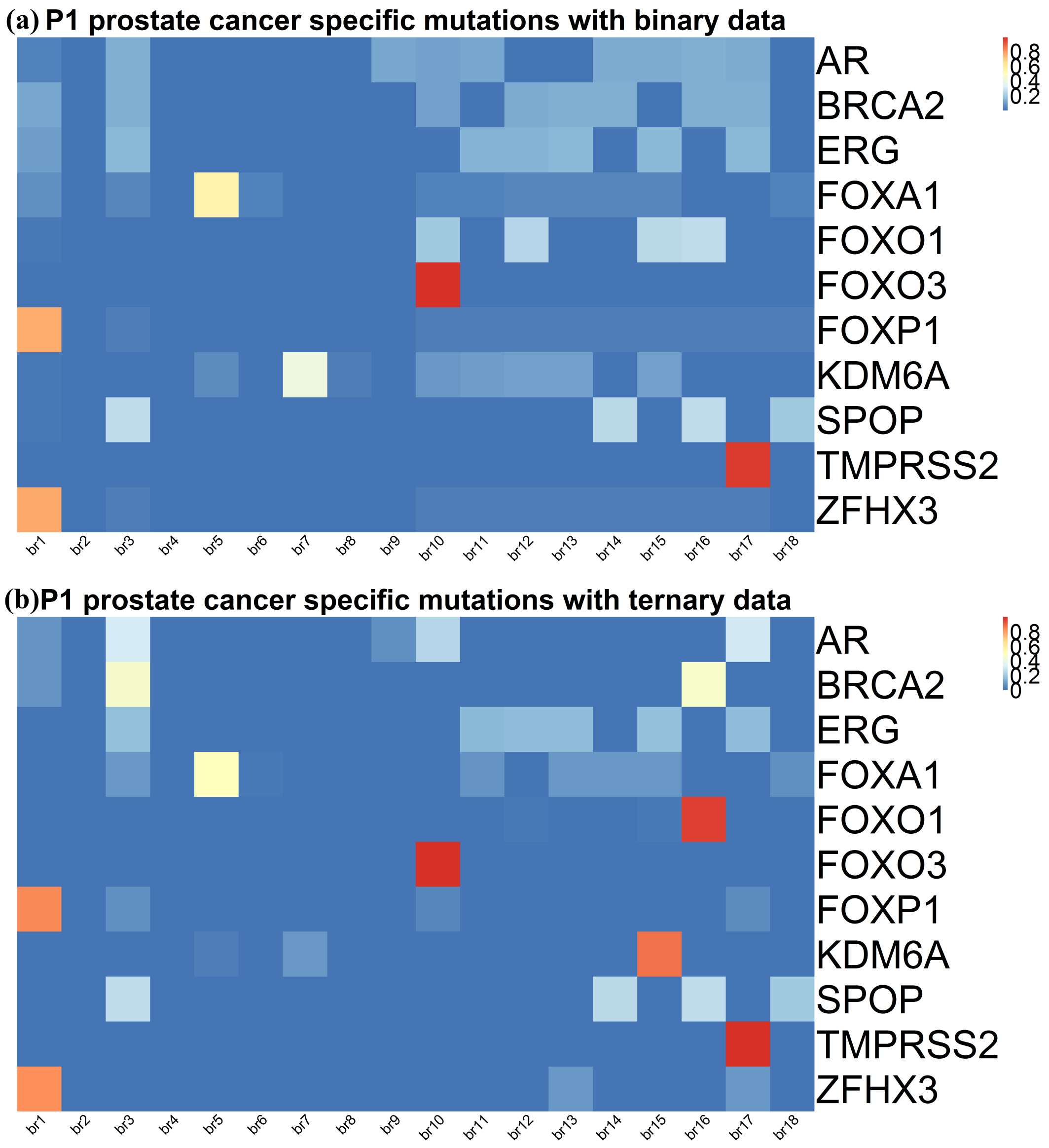

Fig V. Heatmap of posterior probabilities that each mutation occurs on each branch for prostate cancer-specific genes for prostate cancer patient P1 with prior distributions that have large variances. This heatmap is for the prostate cancer-specific genes. Color indicates the magnitude of the probability, with red indicating probability close to 1 and blue indicating probability close to 0. Prior distributions for the parameters are as in Fig R (larger variance).

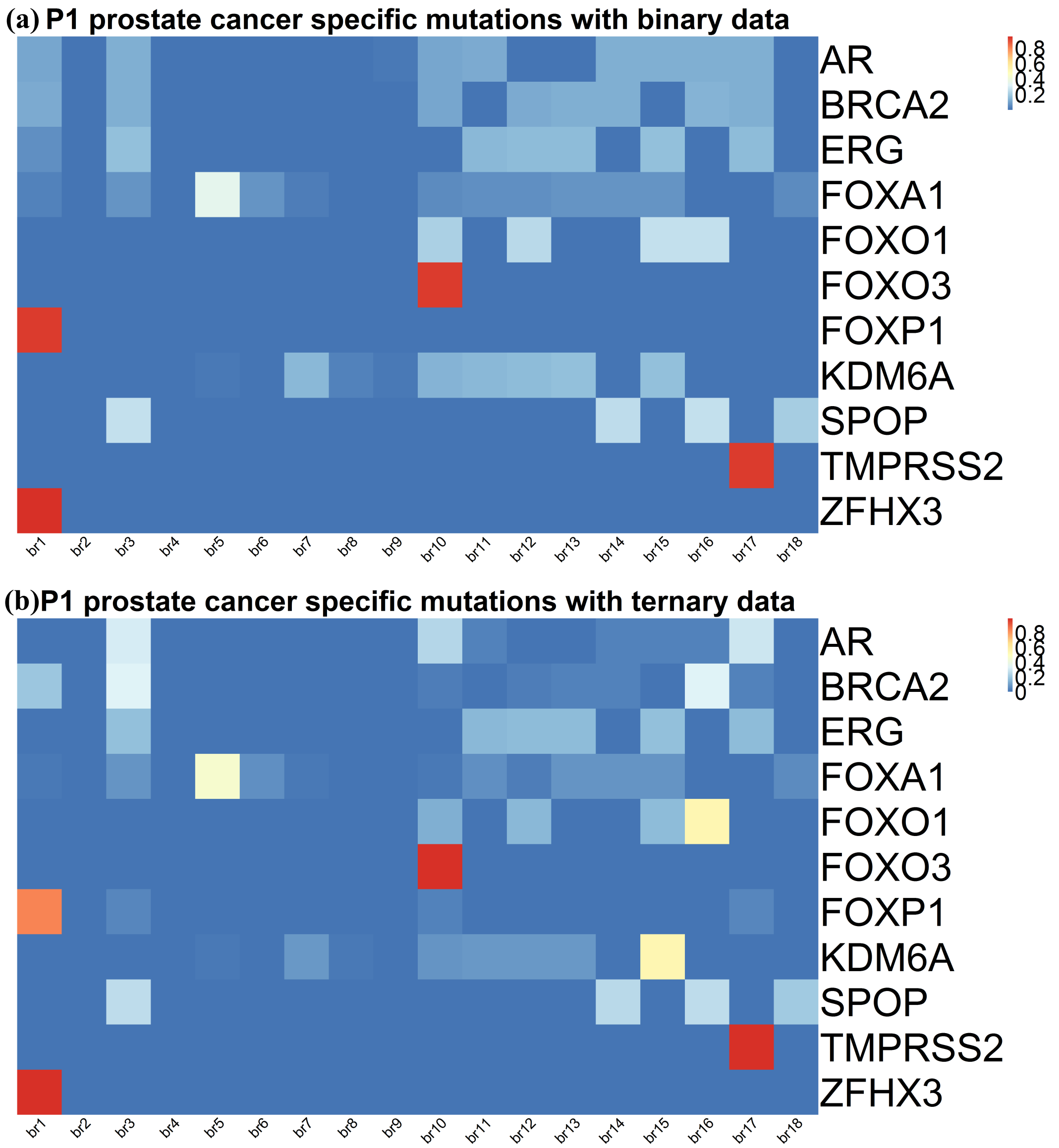

Fig W. Heatmap of posterior probabilities that each mutation occurs on each branch for prostate cancer-specific genes for prostate cancer patient P1 with prior distributions that have small variances. Color indicates the magnitude of the probability, with red indicating probability close to 1 and blue indicating probability close to 0. Prior distributions for the parameters are as in Fig S (smaller variance).

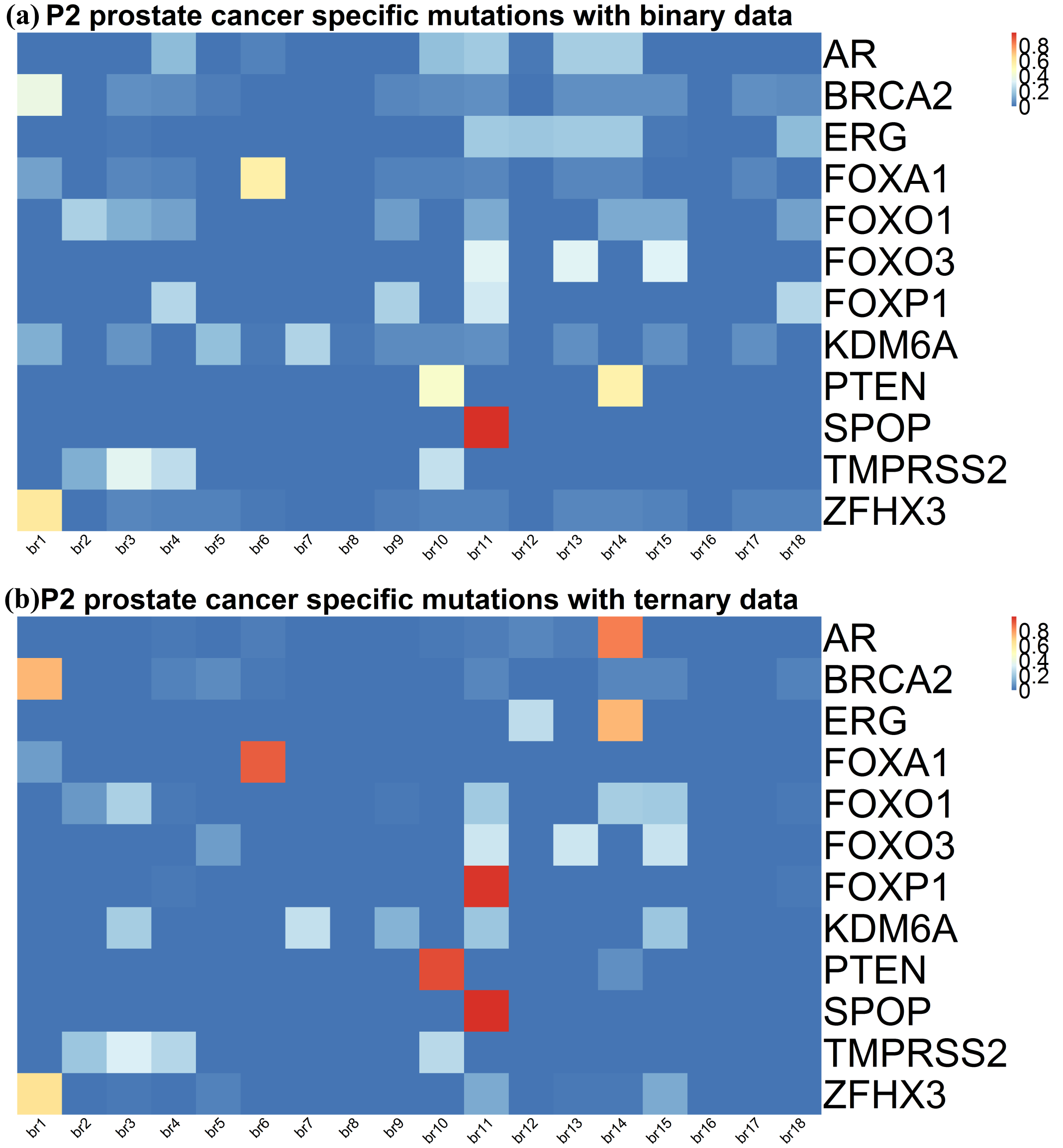

Fig X. Heatmap of posterior probabilities that each mutation occurs on each branch for prostate cancer-specific genes for prostate cancer patient P2 with prior distributions that have large variances. Color indicates the magnitude of the probability, with red indicating probability close to 1 and blue indicating probability close to 0. Prior distributions for the parameters are as in Fig T (larger variance).

(a) P2 prostate cancer specific mutations with binary data

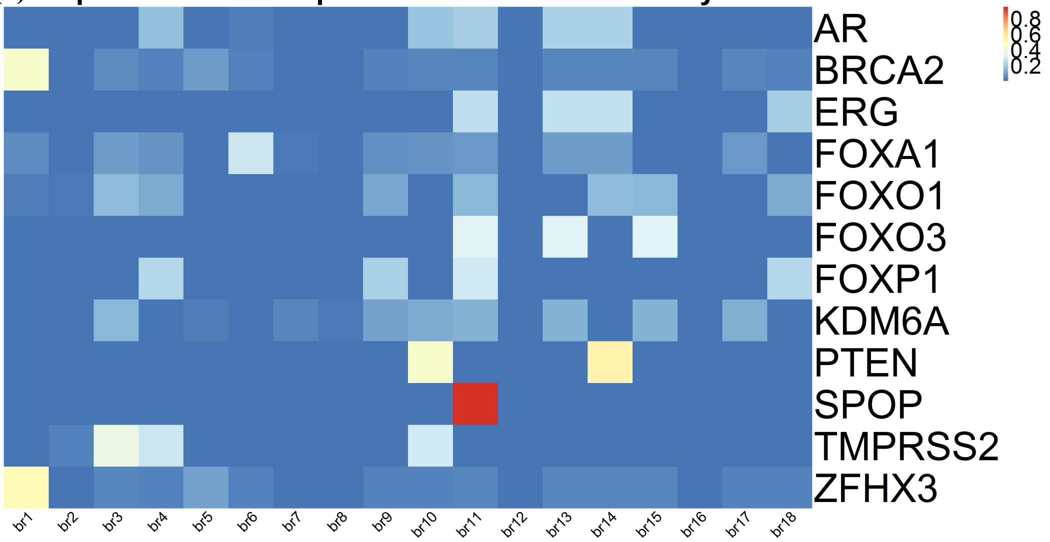

(b)P2 prostate cancer specific mutations with ternary data

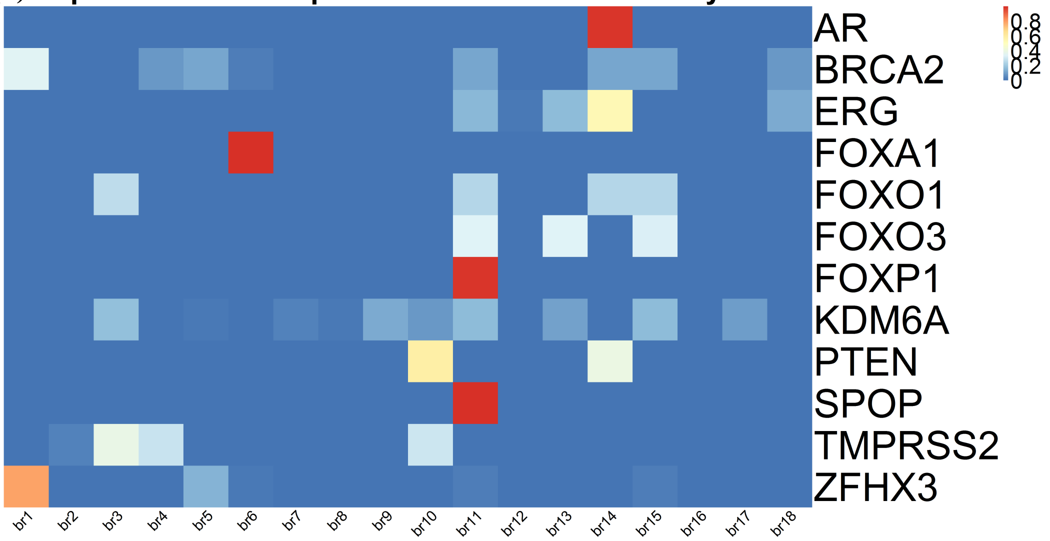

Fig Y. Heatmap of posterior probabilities that each mutation occurs on each branch for prostate cancer-specific genes for prostate cancer patient P2 with prior distributions that have small variances. Color indicates the magnitude of the probability, with red indicating probability close to 1 and blue indicating probability close to 0. Prior distributions for the parameters are as in Fig U (smaller variance).

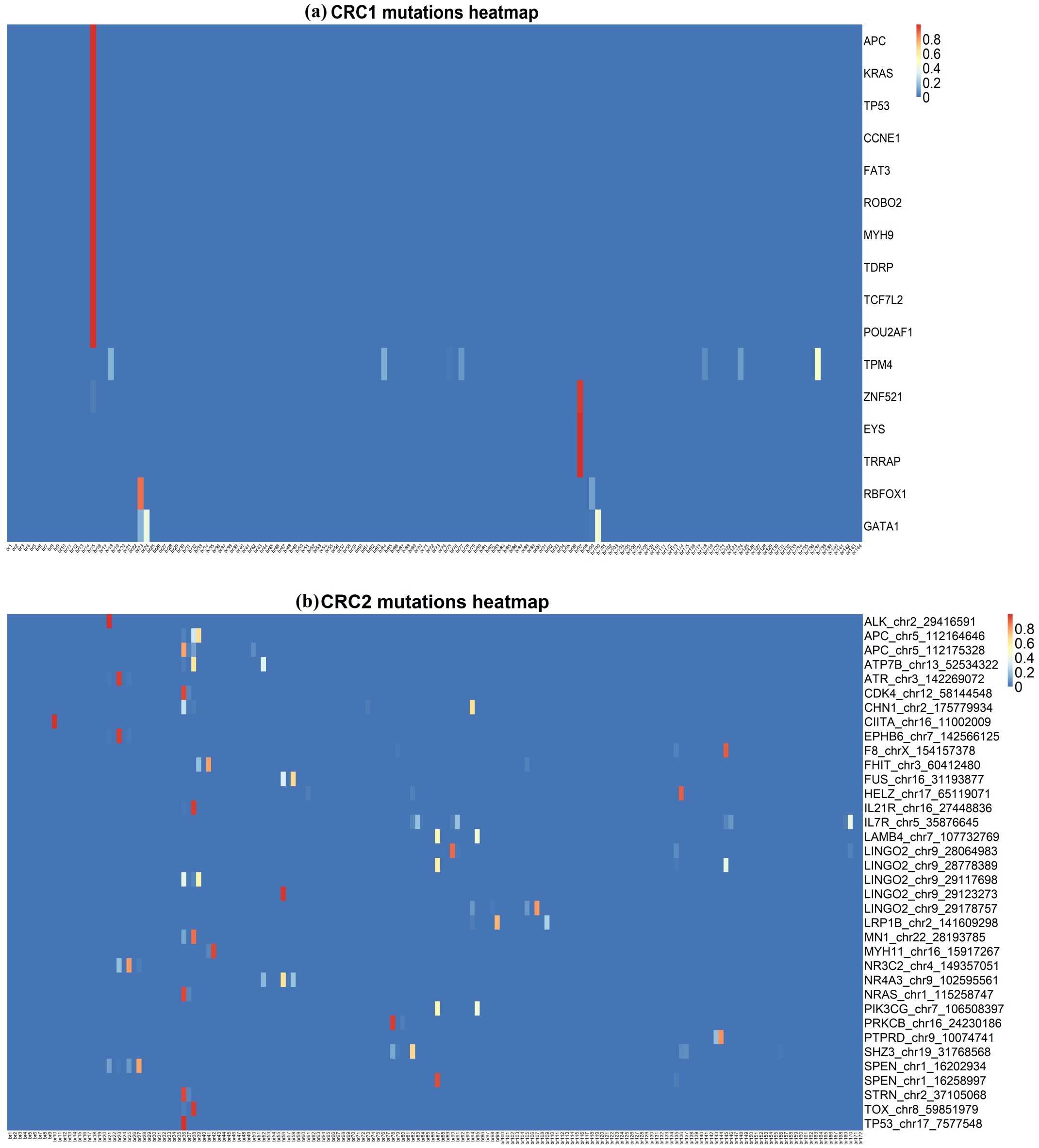

Fig Z. Heatmap of probabilities on each branch for mutations in patient CRC1 and CRC2 with prior distributions that have large variances. Color indicates the magnitude of the probability, with red indicating probability close to 1 and blue indicating probability close to 0. Prior distributions for the parameters are set with larger variance.

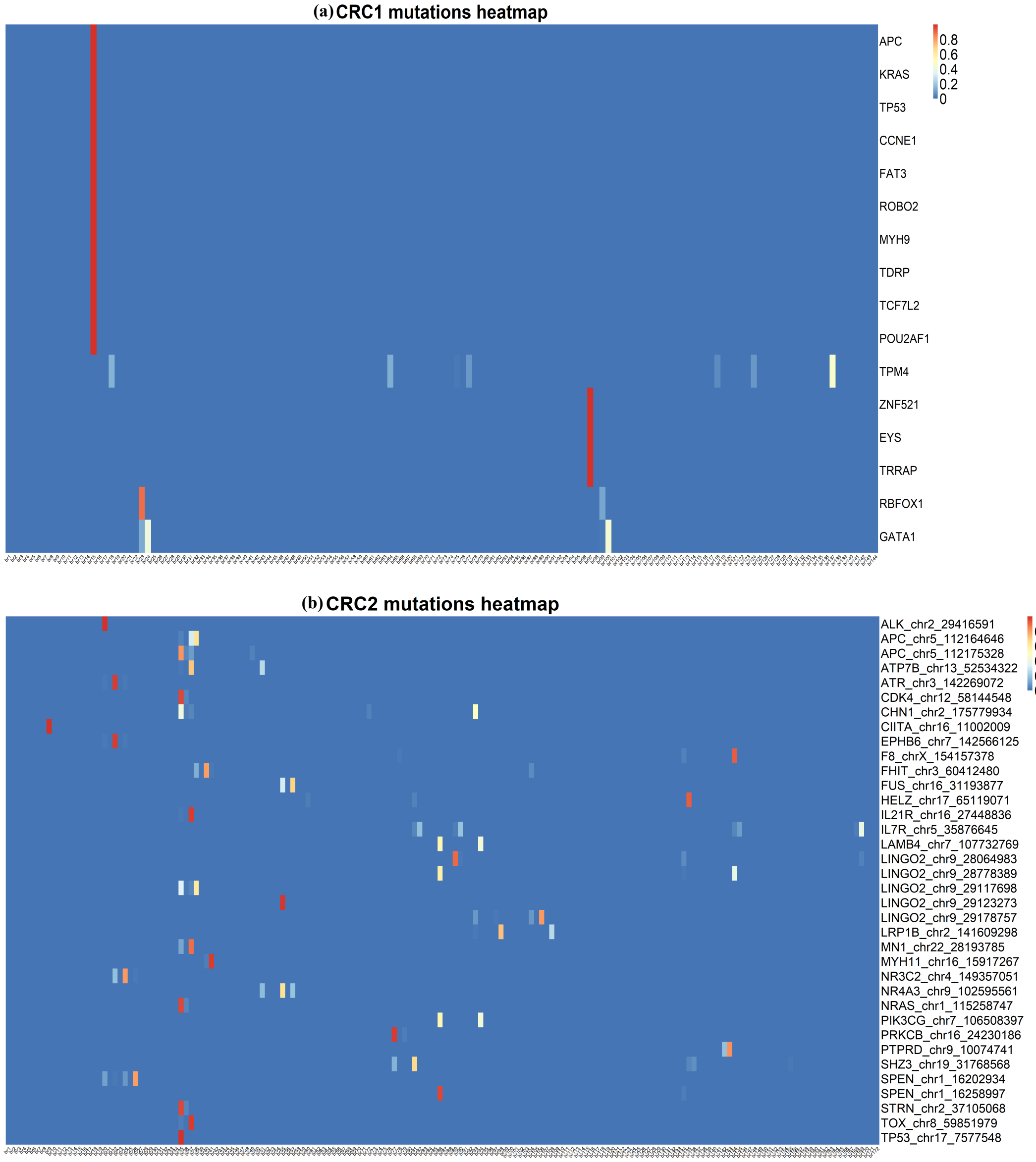

Fig AA. Heatmap of probabilities on each branch for mutations in patient CRC1 and CRC2 with prior distributions that have small variances. Color indicates the magnitude of the probability, with red indicating probability close to 1 and blue indicating probability close to 0. Prior distributions for the parameters are set with smaller variance.

Table A. Location accuracy of MO in scenario 1. Each cell corresponds to unique α, β, type of genotype and missing data percentage.

			**order accuracy**					
α	β	no. of mutations	ternary complete	ternary 10% missing	ternary 20% missing	binary complete	binary 10% missing	binary 20%missing
0	0	20	1.0000	0.9141	0.8288	1.0000	0.9178	0.8377
0.05	0	20	0.9970	0.8631	0.7537	0.9970	0.8681	0.7572
0.1	0	20	0.9945	0.8510	0.7475	0.9945	0.8605	0.7535
0	0.1	20	0.9696	0.9097	0.8530	0.9732	0.9190	0.8649
0.05	0.1	20	0.9647	0.8925	0.8156	0.9709	0.9023	0.8322
0.1	0.1	20	0.9684	0.8938	0.8192	0.9668	0.8984	0.8347
0	0.25	20	0.9553	0.9063	0.8643	0.9532	0.9074	0.8670
0.05	0.25	20	0.9424	0.8853	0.8321	0.9544	0.9023	0.8589
0.1	0.25	20	0.9446	0.8919	0.8371	0.9430	0.9013	0.8519
0	0.5	20	0.8881	0.8635	0.8401	0.8819	0.8616	0.8395
0.05	0.5	20	0.8849	0.8687	0.8444	0.8718	0.8594	0.8395
0.1	0.5	20	0.8585	0.8383	0.8111	0.8402	0.8231	0.8029
0	0	40	1.0000	0.9165	0.8116	1.0000	0.9212	0.8208
0.05	0	40	0.9988	0.8550	0.7245	0.9988	0.8600	0.7278
0.1	0	40	0.9968	0.8415	0.7148	0.9968	0.8445	0.7163
0	0.1	40	0.9721	0.9097	0.8450	0.9739	0.9169	0.8566
0.05	0.1	40	0.9707	0.9035	0.8285	0.9728	0.9091	0.8434
0.1	0.1	40	0.9682	0.8954	0.8202	0.9664	0.9011	0.8329
0	0.25	40	0.9409	0.8936	0.8421	0.9434	0.9031	0.8538
0.05	0.25	40	0.9383	0.8899	0.8350	0.9410	0.8989	0.8480
0.1	0.25	40	0.9289	0.8776	0.8187	0.9368	0.8933	0.8426
0	0.5	40	0.8984	0.8777	0.8554	0.8974	0.8795	0.8576
0.05	0.5	40	0.8740	0.8487	0.8256	0.8733	0.8515	0.8342
0.1	0.5	40	0.8564	0.8290	0.8044	0.8405	0.8184	0.8010
0	0	80	1.0000	0.9053	0.8104	1.0000	0.9100	0.8203
0.05	0	80	0.9983	0.8676	0.7455	0.9983	0.8718	0.7494
0.1	0	80	0.9970	0.8411	0.7180	0.9970	0.8460	0.7220
0	0.1	80	0.9768	0.9187	0.8561	0.9772	0.9242	0.8653
0.05	0.1	80	0.9679	0.9039	0.8287	0.9691	0.9136	0.8459
0.1	0.1	80	0.9616	0.8861	0.8029	0.9646	0.8970	0.8238
0	0.25	80	0.9413	0.8971	0.8497	0.9444	0.9038	0.8596
0.05	0.25	80	0.9405	0.8899	0.8336	0.9406	0.8955	0.8476
0.1	0.25	80	0.9266	0.8717	0.8107	0.9293	0.8844	0.8318
0	0.5	80	0.8922	0.8717	0.8502	0.8851	0.8660	0.8476
0.05	0.5	80	0.8889	0.8682	0.8396	0.8834	0.8654	0.8408
0.1	0.5	80	0.8456	0.8248	0.8016	0.8321	0.8158	0.7962

Table B. Location accuracy of MO in scenario 2. Each cell corresponds to unique α, β, type of genotype and missing data percentage.

			order accuracy					
α	β	no. of mutations	ternary complete	ternary 10% missing	ternary 20% missing	binary complete	binary 10% missing	binary 20%missing
0	0	20	0.9995	0.9076	0.8022	0.9995	0.9107	0.8094
0.05	0	20	0.9970	0.8173	0.6251	0.9970	0.8238	0.6355
0.1	0	20	0.9945	0.7905	0.5995	0.9945	0.8010	0.6090
0	0.1	20	0.9636	0.9146	0.8509	0.9646	0.9206	0.8615
0.05	0.1	20	0.9735	0.9143	0.8301	0.9730	0.9224	0.8520
0.1	0.1	20	0.9675	0.8877	0.7922	0.9619	0.8958	0.8201
0	0.25	20	0.9381	0.9069	0.8852	0.9381	0.9106	0.8895
0.05	0.25	20	0.9412	0.9070	0.8819	0.9391	0.9097	0.8888
0.1	0.25	20	0.9206	0.8940	0.8572	0.9089	0.8849	0.8578
0	0.5	20	0.8944	0.8938	0.8938	0.8961	0.8956	0.8956
0.05	0.5	20	0.8792	0.8792	0.8780	0.8620	0.8620	0.8608
0.1	0.5	20	0.8496	0.8490	0.8479	0.8022	0.8016	0.8011
0	0	40	1.0000	0.9015	0.7859	1.0000	0.9062	0.7986
0.05	0	40	0.9973	0.8267	0.6439	0.9973	0.8320	0.6552
0.1	0	40	0.9963	0.8025	0.6188	0.9963	0.8123	0.6263
0	0.1	40	0.9742	0.9143	0.8528	0.9727	0.9179	0.8597
0.05	0.1	40	0.9702	0.8980	0.8105	0.9707	0.9084	0.8368
0.1	0.1	40	0.9650	0.8902	0.7936	0.9655	0.9032	0.8220
0	0.25	40	0.9423	0.9159	0.8883	0.9426	0.9186	0.8943
0.05	0.25	40	0.9327	0.9057	0.8713	0.9317	0.9105	0.8835
0.1	0.25	40	0.9220	0.8932	0.8594	0.9084	0.8847	0.8600
0	0.5	40	0.9039	0.9033	0.9019	0.8990	0.8984	0.8970
0.05	0.5	40	0.8745	0.8737	0.8734	0.8716	0.8710	0.8707
0.1	0.5	40	0.8518	0.8512	0.8506	0.8138	0.8132	0.8130
0	0	80	0.9992	0.9043	0.7882	0.9992	0.9091	0.8003
0.05	0	80	0.9986	0.8223	0.6336	0.9986	0.8294	0.6450
0.1	0	80	0.9963	0.8073	0.6165	0.9963	0.8133	0.6256
0	0.1	80	0.9761	0.9229	0.8641	0.9761	0.9261	0.8721
0.05	0.1	80	0.9711	0.9018	0.8205	0.9726	0.9126	0.8430
0.1	0.1	80	0.9621	0.8911	0.8003	0.9643	0.9039	0.8268
0	0.25	80	0.9506	0.9265	0.8976	0.9498	0.9285	0.9023
0.05	0.25	80	0.9332	0.9092	0.8785	0.9354	0.9161	0.8890
0.1	0.25	80	0.9289	0.9033	0.8669	0.9225	0.9009	0.8738
0	0.5	80	0.8993	0.8989	0.8982	0.8966	0.8963	0.8956
0.05	0.5	80	0.8839	0.8824	0.8818	0.8740	0.8730	0.8724
0.1	0.5	80	0.8467	0.8460	0.8451	0.8127	0.8123	0.8116

Table C. Order accuracy of MO in scenario 1. Each cell corresponds to unique α, β, type of genotype and missing data percentage.

			**order accuracy**					
α	β	no. of mutations	ternary complete	ternary 10% missing	ternary 20% missing	binary complete	binary 10% missing	binary 20%missing
0	0	20	1.0000	0.8863	0.7811	1.0000	0.8917	0.7939
0.05	0	20	0.9959	0.7796	0.6221	0.9959	0.7899	0.6285
0.1	0	20	0.9910	0.7427	0.5929	0.9910	0.7684	0.6064
0	0.1	20	0.9708	0.9043	0.8423	0.9730	0.9155	0.8588
0.05	0.1	20	0.9453	0.8619	0.7659	0.9523	0.8753	0.7933
0.1	0.1	20	0.9508	0.8471	0.7455	0.9512	0.8641	0.7881
0	0.25	20	0.9467	0.8910	0.8363	0.9432	0.8945	0.8441
0.05	0.25	20	0.9149	0.8499	0.7737	0.9401	0.8818	0.8269
0.1	0.25	20	0.9201	0.8632	0.7829	0.9288	0.8889	0.8159
0	0.5	20	0.8695	0.8466	0.8171	0.8697	0.8503	0.8239
0.05	0.5	20	0.8351	0.8163	0.7874	0.8461	0.8335	0.8100
0.1	0.5	20	0.7887	0.7601	0.7198	0.8025	0.7797	0.7581
0	0	40	1.0000	0.8963	0.7784	1.0000	0.9032	0.7930
0.05	0	40	0.9966	0.7832	0.6007	0.9966	0.7937	0.6089
0.1	0	40	0.9936	0.7452	0.5733	0.9936	0.7549	0.5776
0	0.1	40	0.9635	0.8923	0.8250	0.9678	0.9041	0.8396
0.05	0.1	40	0.9585	0.8741	0.7762	0.9649	0.8874	0.8053
0.1	0.1	40	0.9522	0.8582	0.7598	0.9520	0.8727	0.7892
0	0.25	40	0.9280	0.8748	0.8190	0.9345	0.8873	0.8355
0.05	0.25	40	0.9163	0.8493	0.7751	0.9255	0.8704	0.8064
0.1	0.25	40	0.9038	0.8396	0.7596	0.9193	0.8687	0.8046
0	0.5	40	0.8613	0.8324	0.8074	0.8607	0.8377	0.8133
0.05	0.5	40	0.8226	0.7885	0.7490	0.8474	0.8180	0.7944
0.1	0.5	40	0.7865	0.7511	0.7143	0.7995	0.7698	0.7483
0	0	80	1.0000	0.8721	0.7561	1.0000	0.8807	0.7746
0.05	0	80	0.9978	0.7933	0.6156	0.9978	0.8028	0.6243
0.1	0	80	0.9964	0.7401	0.5651	0.9964	0.7513	0.5720
0	0.1	80	0.9720	0.9100	0.8436	0.9718	0.9175	0.8549
0.05	0.1	80	0.9539	0.8741	0.7748	0.9575	0.8917	0.8060
0.1	0.1	80	0.9398	0.8461	0.7372	0.9476	0.8629	0.7738
0	0.25	80	0.9262	0.8726	0.8175	0.9289	0.8815	0.8321
0.05	0.25	80	0.9193	0.8590	0.7884	0.9284	0.8739	0.8138
0.1	0.25	80	0.8919	0.8137	0.7264	0.9052	0.8453	0.7736
0	0.5	80	0.8598	0.8337	0.8074	0.8560	0.8327	0.8097
0.05	0.5	80	0.8383	0.8105	0.7751	0.8496	0.8272	0.7992
0.1	0.5	80	0.7774	0.7489	0.7178	0.7976	0.7756	0.7511

Table D. Order accuracy of MO in scenario 2. Each cell corresponds to unique α, β, type of genotype and missing data percentage.

			**order accuracy**					
α	β	no. of mutations	ternary complete	ternary 10% missing	ternary 20% missing	binary complete	binary 10% missing	binary 20%missing
0	0	20	1.0000	0.9402	0.8612	1.0000	0.9429	0.8685
0.05	0	20	0.9991	0.8048	0.5025	0.9991	0.8142	0.5435
0.1	0	20	0.9963	0.7510	0.4039	0.9963	0.7779	0.4226
0	0.1	20	0.9890	0.9399	0.8720	0.9863	0.9386	0.8744
0.05	0.1	20	0.9800	0.9269	0.8395	0.9793	0.9367	0.8662
0.1	0.1	20	0.9723	0.9019	0.8079	0.9714	0.9094	0.8459
0	0.25	20	0.9665	0.9280	0.9138	0.9669	0.9335	0.9201
0.05	0.25	20	0.9624	0.9320	0.9080	0.9555	0.9293	0.9114
0.1	0.25	20	0.9361	0.9101	0.8680	0.9252	0.9000	0.8764
0	0.5	20	0.9335	0.9315	0.9315	0.9356	0.9335	0.9335
0.05	0.5	20	0.9136	0.9136	0.9136	0.9035	0.9035	0.9035
0.1	0.5	20	0.8769	0.8769	0.8765	0.8527	0.8527	0.8523
0	0	40	1.0000	0.9135	0.8034	1.0000	0.9187	0.8177
0.05	0	40	0.9990	0.8303	0.5308	0.9990	0.8377	0.5678
0.1	0	40	0.9975	0.7635	0.4210	0.9975	0.7871	0.4438
0	0.1	40	0.9875	0.9390	0.8921	0.9869	0.9419	0.8969
0.05	0.1	40	0.9813	0.9193	0.8301	0.9797	0.9277	0.8581
0.1	0.1	40	0.9722	0.9041	0.7930	0.9719	0.9146	0.8303
0	0.25	40	0.9718	0.9505	0.9283	0.9691	0.9485	0.9292
0.05	0.25	40	0.9500	0.9273	0.8880	0.9511	0.9341	0.9045
0.1	0.25	40	0.9423	0.9162	0.8770	0.9265	0.9064	0.8807
0	0.5	40	0.9536	0.9527	0.9507	0.9547	0.9538	0.9519
0.05	0.5	40	0.9121	0.9119	0.9115	0.9067	0.9064	0.9063
0.1	0.5	40	0.8683	0.8678	0.8661	0.8518	0.8519	0.8519
0	0	80	1.0000	0.9193	0.8173	1.0000	0.9236	0.8315
0.05	0	80	0.9988	0.7997	0.5131	0.9988	0.8118	0.5447
0.1	0	80	0.9963	0.7682	0.4184	0.9963	0.7817	0.4469
0	0.1	80	0.9873	0.9443	0.8955	0.9874	0.9472	0.9015
0.05	0.1	80	0.9784	0.9214	0.8428	0.9792	0.9303	0.8644
0.1	0.1	80	0.9671	0.9033	0.8060	0.9686	0.9159	0.8372
0	0.25	80	0.9693	0.9482	0.9214	0.9695	0.9506	0.9258
0.05	0.25	80	0.9556	0.9358	0.9062	0.9611	0.9449	0.9206
0.1	0.25	80	0.9445	0.9211	0.8815	0.9425	0.9237	0.8977
0	0.5	80	0.9418	0.9416	0.9405	0.9417	0.9416	0.9407
0.05	0.5	80	0.9141	0.9125	0.9120	0.9109	0.9099	0.9091
0.1	0.5	80	0.8759	0.8748	0.8744	0.8537	0.8532	0.8525

Table E. Adjacent order accuracy of MO in scenario 1. Each cell corresponds to unique α, β, type of genotype and missing data percentage.

			**order accuracy**					
α	β	no. of mutations	ternary complete	ternary 10% missing	ternary 20% missing	binary complete	binary 10% missing	binary 20%missing
0	0	20	1.0000	0.8379	0.7179	1.0000	0.8424	0.7261
0.05	0	20	0.9932	0.7590	0.6357	0.9932	0.7641	0.6391
0.1	0	20	0.9861	0.7573	0.6443	0.9861	0.7719	0.6486
0	0.1	20	0.9564	0.8671	0.7955	0.9612	0.8832	0.8189
0.05	0.1	20	0.9301	0.8308	0.7312	0.9351	0.8439	0.7573
0.1	0.1	20	0.9395	0.8266	0.7308	0.9400	0.8427	0.7608
0	0.25	20	0.9209	0.8525	0.7903	0.9178	0.8582	0.8009
0.05	0.25	20	0.8832	0.8093	0.7407	0.9118	0.8477	0.7963
0.1	0.25	20	0.8958	0.8347	0.7560	0.9058	0.8609	0.7856
0	0.5	20	0.8118	0.7850	0.7554	0.8223	0.8032	0.7761
0.05	0.5	20	0.7991	0.7788	0.7494	0.8166	0.8034	0.7774
0.1	0.5	20	0.7643	0.7305	0.6865	0.7911	0.7636	0.7386
0	0	40	1.0000	0.8566	0.7121	1.0000	0.8649	0.7264
0.05	0	40	0.9947	0.7563	0.6007	0.9947	0.7623	0.6046
0.1	0	40	0.9903	0.7319	0.5886	0.9903	0.7348	0.5894
0	0.1	40	0.9451	0.8480	0.7610	0.9505	0.8641	0.7811
0.05	0.1	40	0.9397	0.8424	0.7354	0.9488	0.8569	0.7651
0.1	0.1	40	0.9370	0.8310	0.7339	0.9369	0.8477	0.7619
0	0.25	40	0.8873	0.8246	0.7591	0.8976	0.8446	0.7840
0.05	0.25	40	0.8867	0.8156	0.7447	0.8954	0.8366	0.7739
0.1	0.25	40	0.8765	0.8156	0.7341	0.9000	0.8526	0.7867
0	0.5	40	0.8123	0.7799	0.7495	0.8260	0.8007	0.7727
0.05	0.5	40	0.7925	0.7568	0.7220	0.8172	0.7871	0.7619
0.1	0.5	40	0.7727	0.7377	0.7035	0.7921	0.7645	0.7423
0	0	80	1.0000	0.8310	0.6939	1.0000	0.8398	0.7101
0.05	0	80	0.9965	0.7776	0.6275	0.9965	0.7824	0.6294
0.1	0	80	0.9940	0.7313	0.5863	0.9940	0.7369	0.5879
0	0.1	80	0.9559	0.8753	0.7946	0.9551	0.8857	0.8098
0.05	0.1	80	0.9322	0.8340	0.7270	0.9363	0.8553	0.7596
0.1	0.1	80	0.9163	0.8045	0.6928	0.9226	0.8223	0.7241
0	0.25	80	0.8886	0.8259	0.7586	0.8968	0.8423	0.7828
0.05	0.25	80	0.8952	0.8281	0.7553	0.9036	0.8421	0.7803
0.1	0.25	80	0.8684	0.7892	0.7103	0.8822	0.8186	0.7491
0	0.5	80	0.8170	0.7869	0.7587	0.8224	0.7968	0.7731
0.05	0.5	80	0.8006	0.7703	0.7324	0.8178	0.7938	0.7628
0.1	0.5	80	0.7583	0.7294	0.7004	0.7908	0.7685	0.7467

Table F. Adjacent order accuracy of MO in scenario 2. Each cell corresponds to unique α, β, type of genotype and missing data percentage.

			**order accuracy**					
α	β	no. of mutations	ternary complete	ternary 10% missing	ternary 20% missing	binary complete	binary 10% missing	binary 20%missing
0	0	20	1.0000	0.8623	0.7111	1.0000	0.8691	0.7255
0.05	0	20	0.9978	0.6703	0.4119	0.9978	0.6784	0.4229
0.1	0	20	0.9894	0.6088	0.3958	0.9894	0.6361	0.3995
0	0.1	20	0.9603	0.8816	0.7820	0.9603	0.8914	0.7993
0.05	0.1	20	0.9458	0.8617	0.7408	0.9427	0.8798	0.7895
0.1	0.1	20	0.9399	0.8273	0.7005	0.9459	0.8589	0.7583
0	0.25	20	0.9112	0.8529	0.8259	0.9199	0.8764	0.8512
0.05	0.25	20	0.9258	0.8738	0.8312	0.9182	0.8730	0.8406
0.1	0.25	20	0.8850	0.8487	0.8049	0.8947	0.8620	0.8338
0	0.5	20	0.8347	0.8319	0.8319	0.8626	0.8598	0.8598
0.05	0.5	20	0.8371	0.8371	0.8371	0.8562	0.8562	0.8562
0.1	0.5	20	0.7946	0.7946	0.7935	0.8129	0.8129	0.8118
0	0	40	1.0000	0.8432	0.6650	1.0000	0.8516	0.6851
0.05	0	40	0.9985	0.7160	0.4890	0.9985	0.7249	0.4995
0.1	0	40	0.9933	0.6578	0.4566	0.9933	0.6769	0.4608
0	0.1	40	0.9628	0.8744	0.8043	0.9634	0.8878	0.8227
0.05	0.1	40	0.9536	0.8373	0.7028	0.9519	0.8610	0.7586
0.1	0.1	40	0.9363	0.8424	0.6958	0.9471	0.8720	0.7579
0	0.25	40	0.9243	0.8886	0.8527	0.9298	0.8971	0.8676
0.05	0.25	40	0.8887	0.8534	0.7876	0.9029	0.8792	0.8317
0.1	0.25	40	0.8909	0.8521	0.8028	0.8960	0.8702	0.8358
0	0.5	40	0.8798	0.8798	0.8791	0.8968	0.8968	0.8962
0.05	0.5	40	0.8247	0.8240	0.8237	0.8548	0.8539	0.8539
0.1	0.5	40	0.7991	0.7984	0.7950	0.8293	0.8286	0.8281
0	0	80	0.9999	0.8499	0.6863	0.9999	0.8572	0.7062
0.05	0	80	0.9962	0.6665	0.4433	0.9962	0.6817	0.4545
0.1	0	80	0.9899	0.6605	0.4426	0.9899	0.6717	0.4494
0	0.1	80	0.9646	0.8990	0.8250	0.9651	0.9074	0.8419
0.05	0.1	80	0.9451	0.8462	0.7306	0.9476	0.8683	0.7743
0.1	0.1	80	0.9269	0.8299	0.6970	0.9347	0.8575	0.7443
0	0.25	80	0.9190	0.8866	0.8447	0.9236	0.8968	0.8598
0.05	0.25	80	0.8893	0.8609	0.8199	0.9135	0.8921	0.8587
0.1	0.25	80	0.9014	0.8717	0.8153	0.9128	0.8888	0.8569
0	0.5	80	0.8578	0.8572	0.8550	0.8711	0.8709	0.8688
0.05	0.5	80	0.8257	0.8221	0.8214	0.8494	0.8467	0.8458
0.1	0.5	80	0.8053	0.8044	0.8029	0.8269	0.8265	0.8259

Table G. Credible set accuracy of MO in scenario 1. Each cell corresponds to unique α, β, type of genotype and missing data percentage.

			**order accuracy**					
α	β	no. of mutations	ternary complete	ternary 10% missing	ternary 20% missing	binary complete	binary 10% missing	binary 20%missing
0	0	20	1.0000	0.9534	0.9042	1.0000	0.9545	0.9052
0.05	0	20	0.9990	0.9086	0.8257	0.9990	0.9136	0.8347
0.1	0	20	1.0000	0.9075	0.8195	1.0000	0.9115	0.8260
0	0.1	20	0.9871	0.9691	0.9464	0.9892	0.9783	0.9613
0.05	0.1	20	0.9964	0.9813	0.9590	0.9979	0.9891	0.9761
0.1	0.1	20	0.9959	0.9839	0.9684	0.9984	0.9907	0.9813
0	0.25	20	0.9860	0.9790	0.9639	0.9919	0.9882	0.9768
0.05	0.25	20	0.9945	0.9846	0.9748	0.9995	0.9945	0.9907
0.1	0.25	20	0.9945	0.9879	0.9770	0.9989	0.9951	0.9918
0	0.5	20	0.9809	0.9779	0.9736	0.9889	0.9871	0.9852
0.05	0.5	20	0.9944	0.9919	0.9876	0.9963	0.9944	0.9919
0.1	0.5	20	0.9924	0.9893	0.9842	0.9962	0.9949	0.9937
0	0	40	1.0000	0.9544	0.8888	1.0000	0.9555	0.8920
0.05	0	40	1.0000	0.8985	0.7955	1.0000	0.9055	0.8050
0.1	0	40	1.0000	0.8890	0.7818	1.0000	0.8943	0.7890
0	0.1	40	0.9881	0.9721	0.9549	0.9925	0.9817	0.9678
0.05	0.1	40	0.9938	0.9831	0.9638	0.9972	0.9926	0.9820
0.1	0.1	40	0.9946	0.9850	0.9657	0.9977	0.9923	0.9783
0	0.25	40	0.9861	0.9747	0.9630	0.9905	0.9831	0.9763
0.05	0.25	40	0.9929	0.9875	0.9742	0.9978	0.9946	0.9891
0.1	0.25	40	0.9943	0.9883	0.9769	0.9989	0.9957	0.9908
0	0.5	40	0.9824	0.9790	0.9756	0.9895	0.9870	0.9849
0.05	0.5	40	0.9901	0.9880	0.9840	0.9960	0.9941	0.9923
0.1	0.5	40	0.9907	0.9885	0.9854	0.9950	0.9935	0.9916
0	0	80	1.0000	0.9486	0.8909	1.0000	0.9497	0.8934
0.05	0	80	0.9998	0.9144	0.8243	0.9998	0.9221	0.8344
0.1	0	80	0.9998	0.8940	0.7938	0.9998	0.9005	0.8016
0	0.1	80	0.9934	0.9804	0.9591	0.9961	0.9865	0.9720
0.05	0.1	80	0.9942	0.9833	0.9634	0.9970	0.9893	0.9799
0.1	0.1	80	0.9948	0.9840	0.9661	0.9976	0.9916	0.9818
0	0.25	80	0.9883	0.9802	0.9678	0.9929	0.9885	0.9820
0.05	0.25	80	0.9942	0.9884	0.9784	0.9967	0.9937	0.9886
0.1	0.25	80	0.9949	0.9881	0.9749	0.9979	0.9955	0.9910
0	0.5	80	0.9782	0.9750	0.9709	0.9881	0.9864	0.9845
0.05	0.5	80	0.9898	0.9870	0.9825	0.9975	0.9957	0.9941
0.1	0.5	80	0.9910	0.9885	0.9863	0.9967	0.9961	0.9952

Table H. Credible set accuracy of MO in scenario 2. Each cell corresponds to unique α, β, type of genotype and missing data percentage.

			order accuracy					
α	β	no. of mutations	ternary complete	ternary 10% missing	ternary 20% missing	binary complete	binary 10% missing	binary 20%missing
0	0	20	1.0000	0.9476	0.8749	1.0000	0.9481	0.8780
0.05	0	20	0.9980	0.8707	0.7109	0.9980	0.8797	0.7304
0.1	0	20	0.9985	0.8485	0.6880	0.9985	0.8600	0.7035
0	0.1	20	0.9833	0.9712	0.9510	0.9863	0.9757	0.9636
0.05	0.1	20	0.9929	0.9847	0.9673	0.9964	0.9908	0.9827
0.1	0.1	20	0.9959	0.9853	0.9670	0.9980	0.9914	0.9812
0	0.25	20	0.9730	0.9662	0.9588	0.9783	0.9746	0.9693
0.05	0.25	20	0.9920	0.9904	0.9872	0.9957	0.9936	0.9931
0.1	0.25	20	0.9952	0.9931	0.9861	0.9989	0.9973	0.9947
0	0.5	20	0.9720	0.9720	0.9720	0.9743	0.9743	0.9743
0.05	0.5	20	0.9929	0.9929	0.9923	0.9952	0.9952	0.9952
0.1	0.5	20	0.9936	0.9936	0.9936	0.9994	0.9994	0.9994
0	0	40	1.0000	0.9402	0.8636	1.0000	0.9428	0.8678
0.05	0	40	0.9980	0.8792	0.7348	0.9980	0.8866	0.7483
0.1	0	40	0.9998	0.8588	0.7058	0.9998	0.8738	0.7198
0	0.1	40	0.9916	0.9773	0.9569	0.9929	0.9824	0.9689
0.05	0.1	40	0.9944	0.9829	0.9633	0.9962	0.9913	0.9804
0.1	0.1	40	0.9982	0.9888	0.9696	0.9990	0.9951	0.9893
0	0.25	40	0.9823	0.9765	0.9681	0.9874	0.9842	0.9808
0.05	0.25	40	0.9947	0.9913	0.9844	0.9960	0.9944	0.9931
0.1	0.25	40	0.9955	0.9923	0.9848	0.9987	0.9981	0.9963
0	0.5	40	0.9792	0.9792	0.9792	0.9855	0.9855	0.9855
0.05	0.5	40	0.9924	0.9924	0.9924	0.9965	0.9965	0.9965
0.1	0.5	40	0.9924	0.9924	0.9921	0.9985	0.9985	0.9985
0	0	80	0.9995	0.9450	0.8711	0.9995	0.9455	0.8750
0.05	0	80	0.9994	0.8752	0.7224	0.9994	0.8862	0.7398
0.1	0	80	0.9994	0.8643	0.7100	0.9994	0.8750	0.7253
0	0.1	80	0.9912	0.9766	0.9591	0.9934	0.9830	0.9703
0.05	0.1	80	0.9960	0.9864	0.9676	0.9974	0.9912	0.9813
0.1	0.1	80	0.9965	0.9872	0.9635	0.9981	0.9942	0.9815
0	0.25	80	0.9881	0.9833	0.9754	0.9911	0.9882	0.9836
0.05	0.25	80	0.9954	0.9918	0.9870	0.9971	0.9958	0.9939
0.1	0.25	80	0.9977	0.9946	0.9894	0.9989	0.9977	0.9958
0	0.5	80	0.9766	0.9766	0.9765	0.9861	0.9861	0.9861
0.05	0.5	80	0.9946	0.9946	0.9945	0.9984	0.9984	0.9983
0.1	0.5	80	0.9942	0.9940	0.9939	0.9987	0.9987	0.9987

Table I. Location accuracy of MO when the mutation process is modeled using the multinomial distribution in scenario 1 and 2 for binary data. Each cell corresponds to a unique α, β, number of mutations, missing data percentage and scenario setting.

			**location accuracy**					
α	β	no. of mutations	scenario 1 complete	scenario 1 10% missing	scenario 1 20% missing	scenario 2 complete	scenario 2 10% missing	scenario 2 20%missing
0	0	20	1.0000	0.9500	0.8800	1.0000	0.9500	0.9100
0.05	0	20	1.0000	0.9100	0.8400	1.0000	0.9200	0.8600
0.1	0	20	0.9800	0.8900	0.8200	1.0000	0.9500	0.9300
0	0.1	20	0.9700	0.9000	0.7600	0.9700	0.9300	0.9000
0.05	0.1	20	0.9900	0.9200	0.8300	0.9700	0.8900	0.8500
0.1	0.1	20	0.9900	0.9200	0.8300	0.9800	0.9400	0.8400
0	0.25	20	0.9900	0.9400	0.9000	0.9600	0.9100	0.9100
0.05	0.25	20	0.9500	0.8800	0.8400	0.9400	0.9200	0.9100
0.1	0.25	20	0.9400	0.8700	0.8300	0.8900	0.8400	0.8500
0	0.5	20	0.8100	0.8000	0.7600	0.8600	0.8500	0.8500
0.05	0.5	20	0.8500	0.8400	0.8100	0.8500	0.8500	0.8500
0.1	0.5	20	0.8600	0.8500	0.8200	0.7800	0.7800	0.7800
0	0	40	1.0000	0.9200	0.8000	1.0000	0.9600	0.9200
0.05	0	40	0.9900	0.9400	0.8100	1.0000	0.9600	0.9300
0.1	0	40	0.9800	0.9300	0.8000	1.0000	0.9700	0.9100
0	0.1	40	0.9600	0.9200	0.8100	0.9700	0.9500	0.9100
0.05	0.1	40	0.9900	0.9500	0.8700	0.9800	0.9100	0.8400
0.1	0.1	40	0.9700	0.9300	0.8600	0.9800	0.9600	0.8900
0	0.25	40	0.9300	0.8600	0.8000	0.9700	0.9600	0.9300
0.05	0.25	40	0.9200	0.8600	0.7800	0.9400	0.9200	0.8900
0.1	0.25	40	0.9000	0.8400	0.7600	0.9300	0.9100	0.8800
0	0.5	40	0.8600	0.8500	0.8200	0.9200	0.9200	0.9200
0.05	0.5	40	0.9000	0.8600	0.8500	0.8500	0.8500	0.8500
0.1	0.5	40	0.8800	0.8400	0.8300	0.8100	0.8100	0.8100
0	0	80	1.0000	0.9300	0.8200	1.0000	0.9500	0.8800
0.05	0	80	0.9900	0.9200	0.8800	0.9900	0.9300	0.9000
0.1	0	80	0.9800	0.9100	0.8700	0.9900	0.9300	0.9000
0	0.1	80	0.9600	0.8900	0.8100	0.9900	0.9700	0.8900
0.05	0.1	80	0.9700	0.9000	0.8200	0.9900	0.9600	0.9000
0.1	0.1	80	0.9600	0.8900	0.8100	0.9900	0.9500	0.9000
0	0.25	80	0.9600	0.8900	0.8300	0.9800	0.9500	0.9400
0.05	0.25	80	0.9300	0.8300	0.8000	0.9500	0.9200	0.9100
0.1	0.25	80	0.9200	0.8300	0.8000	0.9200	0.8900	0.8800
0	0.5	80	0.9500	0.9400	0.9400	0.8400	0.8400	0.8400
0.05	0.5	80	0.8800	0.8700	0.8700	0.8500	0.8500	0.8500
0.1	0.5	80	0.8400	0.8300	0.8400	0.7700	0.7700	0.7700

References

1. Su F, Zhang W, Zhang D, Zhang Y, Pang C, Huang Y, et al. Spatial Intratumor Genomic Heterogeneity within Localized Prostate Cancer Revealed by Single-nucleus Sequencing. European Urology. 2018;74(5):551–559.

2. Leung ML, Davis A, Gao R, Casasent A, Wang Y, Sei E, et al. Single-cell DNA sequencing reveals a late-dissemination model in metastatic colorectal cancer. Genome Research. 2017;27:1287–1299.
